# Chromatin modifiers KMT2D, cBAF, and p300 cooperatively facilitate de novo binding of transcription factors on enhancers

**DOI:** 10.1038/s41467-026-77757-w

**Published:** 2026-09-15

**Authors:** Hieu T. Van, Young-Kwon Park, Danyang Wan, Chengyu Liu, Shamima Islam, Stefania Dell’Orso, Weiqun Peng, Vittorio Sartorelli, Ji-Eun Lee, Kai Ge

**Affiliations:** 1 National Institute of Diabetes and Digestive and Kidney Diseases, National Institutes of Health (NIH), Bethesda, MD USA; 2 Transgenic Core, National Heart, Lung, and Blood Institute, NIH, Bethesda, MD USA; 3 Genome Technology Unit, National Institute of Arthritis and Musculoskeletal and Skin Diseases (NIAMS), NIH, Bethesda, MD USA; 4 Department of Physics and Department of Anatomy and Cell Biology, The George Washington University, Washington, DC USA; 5 Laboratory of Muscle Stem Cells and Gene Regulation, NIAMS, NIH, Bethesda, MD USA

**Keywords:** Epigenomics, Chromatin

## Abstract

Transcription factors (TFs) bind to enhancers and recruit H3K4me1 methyltransferase KMT2D, chromatin remodeler cBAF, and H3K27 acetyltransferase p300 to activate transcription. However, the role of chromatin modifiers in regulating de novo binding of TFs on enhancers remains unclear. Using a robust nuclear translocation system, we show that the muscle lineage-determining TF MyoD binds to chromatin pervasively within one hour, with half of induced MyoD binding sites co-occupied by KMT2D, cBAF, and p300. On the majority of these MyoD^+^ enhancers, acute depletion of KMT2D or short-term inhibition of cBAF or p300 enzymatic activity markedly reduces de novo binding of MyoD as well as that of KMT2D, cBAF, and p300. On enhancers with intact MyoD binding despite these perturbations, we observe cooperative recruitment among chromatin modifiers. Similar interdependent relationships are observed between the signal-dependent TF Glucocorticoid Receptor and KMT2D, cBAF, and p300. Together, our findings show that chromatin modifiers are not only downstream effectors but also required for de novo binding of TFs on enhancers, refining a model of enhancer establishment as a process governed by functional cooperation rather than a strict hierarchy.

## Abstract (web summary)

Here, the authors show that chromatin modifiers are required for transcription factors to bind new enhancers, revealing that enhancer landscapes are built through cooperation rather than a strict step-by-step order.

## Introduction

Cell differentiation, the process by which precursor cells give rise to specialized cell types, is crucial for the development of multicellular organisms. Cell differentiation is determined by cell-type-specific gene expression, which is precisely controlled by transcriptional enhancers. Enhancers are DNA elements enriched for binding motifs of lineage-determining transcription factors (LDTFs). Upon binding to enhancers, LDTFs recruit other TFs, coactivators, and chromatin modifiers, priming these regulatory regions for activation and facilitating RNA Polymerase II recruitment to drive the expression of cell-type specific genes^1^. Enhancers are often characterized by the presence of histone H3K4 mono-methylation (H3K4me1) in their primed states, or both H3K4me1 and H3K27 acetylation (H3K27ac) in their active states^1^.

Key chromatin modifiers, including KMT2C/D, CBP/p300, and the canonical BAF (cBAF) complex, play important roles in enhancer regulation. KMT2C/D (also known as MLL3/4) are major mammalian H3K4me1 methyltransferases enriched on enhancers^2^. KMT2C/D are critical for de novo enhancer activation during cell differentiation and cell fate transitions, with KMT2D holding a more prominent role^2,3^. The histone acetyltransferase p300 (also known as EP300/KAT3B) and its homolog CBP (also known as CREBBP/KAT3A) catalyze H3K27ac^4^ and mark cell-type specific enhancers in mammalian cells^5^. Besides acetylating histones, CBP/p300 contribute to transcription by acetylating non-histone proteins, scaffolding transcriptional machineries, and promoting enhancer-promoter communication^6^. The cBAF complex, one of the three SWI/SNF chromatin remodelers, facilitates chromatin opening by mobilizing and evicting nucleosomes through its enzymatic subunit BRG1 (also known as SMARCA4)^7^. cBAF colocalizes with LDTFs on enhancers and promotes enhancer activation during cell fate transitions^8^. Consistent with their critical roles in enhancer activation, KMT2C/D, CBP/p300, and cBAF are generally required for cell differentiation and animal development^9,10^. Mutations in the encoding genes of these chromatin modifiers are found to drive many types of cancers^11^.

Our previous studies, using various cell differentiation models including adipogenesis, myogenesis, and mouse embryonic stem cell (ESC) differentiation, have identified functional relationships among KMT2D, p300, and cBAF on enhancers. Specifically, loss of KMT2D disrupts p300 recruitment and impairs de novo enhancer activation^2,3,12^, supporting a functional relationship between KMT2D and p300. KMT2D also physically associates with cBAF, and loss of cBAF’s ATPase subunit BRG1 blocks activation of C/EBPβ-bound adipogenic enhancers. cBAF and KMT2D reciprocally regulate each other’s binding on enhancers^8^. Collectively, these findings have been interpreted as supporting a stepwise model of enhancer activation in which LDTFs bind to enhancer regions, followed by recruitment of cBAF and KMT2D, and subsequently p300 to promote enhancer activation^13^. However, observed interdependencies among these factors are also equally consistent with a cooperative model of enhancer assembly. These insights are largely derived from stable gene knockout approaches, which allow for compensatory or indirect effects that might obscure the primary functions of these chromatin modifiers. For example, BRG1 has been reported to act as both a repressor and an activator of transcription in knockout studies, whereas acute depletion of BRG1 proteins indicates that it primarily functions as a transcriptional activator^14^. Additionally, previous studies used models in which LDTFs are stably expressed, which results in a pre-established enhancer landscape and limits the analysis of de novo binding events.

MyoD is the founding member of the myogenic TF family^15^ and plays a crucial role in coordinating transcriptional programs and promoting myogenesis in most cell types^16^. Chromatin modifiers cBAF, KMT2D, and p300 are known to be important for MyoD-mediated enhancer activation, gene induction, and myogenesis^2,17–19^. To overcome limitations of previous studies and to investigate the early dynamics and functional relationships of LDTFs and chromatin modifiers on enhancers, we used a robust MyoD nuclear translocation system, coupled with short-term depletion of KMT2D or chemical inhibition of cBAF or p300 enzymatic activity. We found that within one hour (1h) of its nuclear translocation, MyoD colocalizes with cBAF, KMT2D, and p300 on approximately 50% of enhancers. Acute perturbation of any of these chromatin modifiers disrupts de novo binding of MyoD on most MyoD^+^ enhancers. On enhancers where MyoD binding remained intact, acute perturbation of one chromatin modifier markedly reduced the binding of the others, suggesting that KMT2D, cBAF, and p300 regulate one another on LDTF-bound enhancers. In addition, we used an endogenous nuclear translocation system of the signal-dependent TF (SDTF) Glucocorticoid Receptor (GR) and validated the dynamic and interdependent relationships between TFs and chromatin modifiers on enhancers.

## Results

### Dynamic binding of nuclear translocated MyoD during myogenesis

**Fig. 1:**
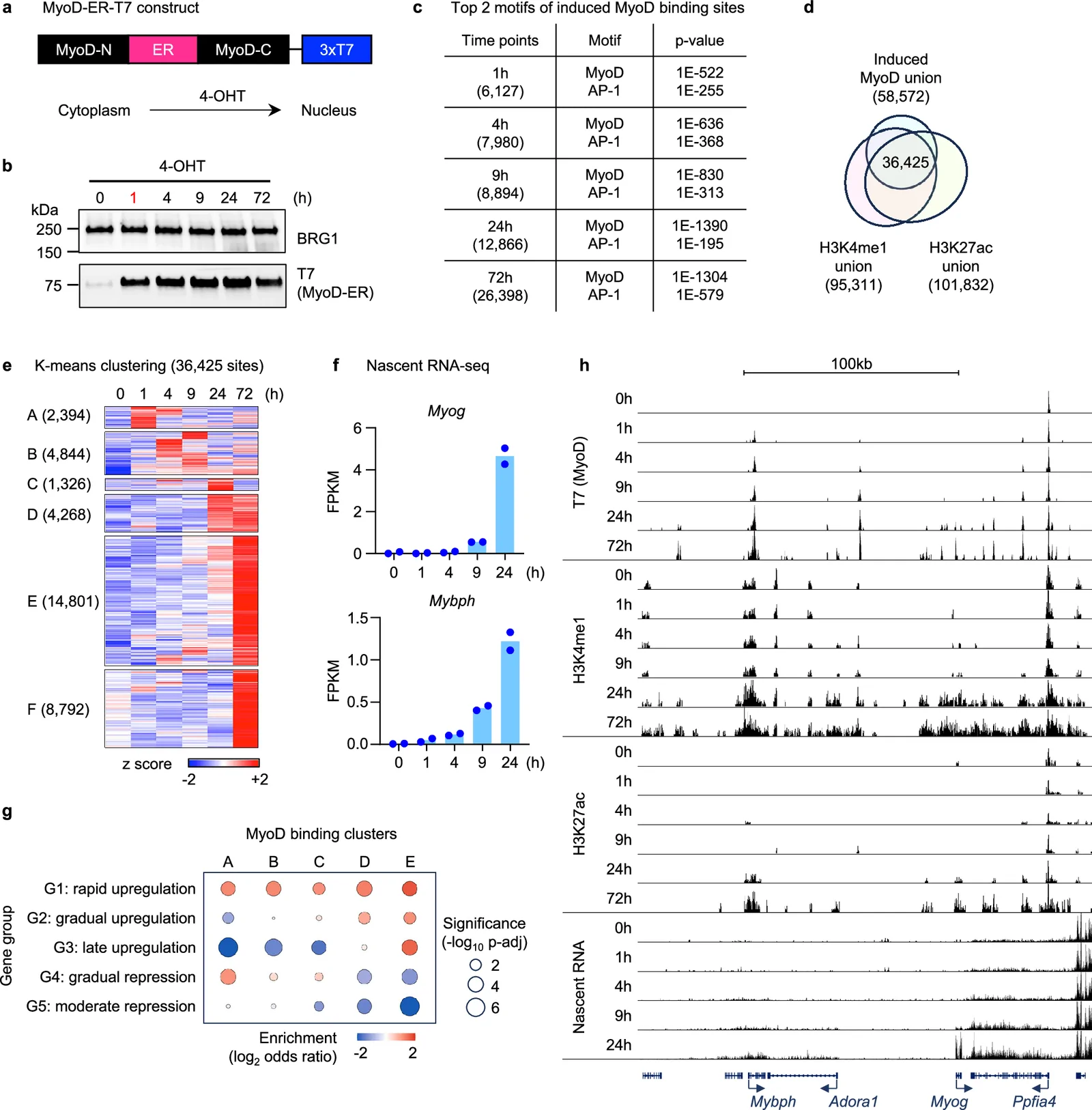
Dynamic binding of nuclear translocated MyoD during myogenesis. **a** Schematic of the 4-OHT-inducible MyoD-ER system. MyoD-ER-T7 expressing preadipocytes were treated with 4-OHT for indicated durations, followed by Western blot (WB), CUT&RUN, and nascent RNA-seq analysis. **b** WB analysis of nuclear extracts. Antibodies used were indicated on the right. BRG1 was used as a loading control. This experiment was repeated independently at least twice with similar results. **c** HOMER motif analysis of induced MyoD binding sites. All regions were used, and only the top 2 motifs are shown. *P* values were determined by a one-sided hypergeometric test without adjustment for multiple comparisons. **d** Venn diagrams depicting 36,425 4-OHT-induced MyoD binding sites used for k-means clustering. **e** Heat maps of k-means clustering depicting MyoD binding dynamics on 36,425 sites defined in (**d**). **f** Nascent RNA expression of myogenic markers *Myog* and *Mybph* (*n* = 2). FPKM values indicate gene expression levels. **g** Bubble plot showing the dynamics of nascent RNA expression for genes associated with MyoD binding clusters A-E defined in (**e**). **h** Genome browser view of MyoD binding, enrichment of enhancer histone marks H3K4me1 and H3K27ac, and nascent RNA induction around the *Myog* locus. Extended analyses of nascent RNA expression dynamics and associated enhancer histone marks (H3K4me1 and H3K27ac) are provided in Supplementary Figs. 2 and 3, respectively.

To overcome the limitations inherent to stably expressed LDTF models and to elucidate the early interplay among LDTFs and chromatin modifiers on enhancers, we utilized a 4-Hydroxytamoxifen (4-OHT)-inducible MyoD-ER nuclear translocation system^20^. We generated a retroviral vector carrying MyoD-ER with three tandem T7 tags at the C-terminus (Fig. 1a) and expressed MyoD-ER-T7 in SV40T-immortalized brown preadipocytes. Preadipocytes were selected as a non-myogenic mesenchymal progenitor model lacking endogenous MyoD and other myogenic factors (Supplementary Fig. 1a), providing a system to examine MyoD-dependent chromatin events during preadipocyte-to-myocyte *trans*-differentiation. Treatment with 4-OHT markedly increased nuclear MyoD levels within 1 h (Fig. 1b). Similar to constitutively expressed MyoD^2^, nuclear translocated MyoD-ER-T7 induced the *trans*-differentiation of preadipocytes into myocytes and the associated expression of myogenic marker genes *Myogenin (Myog)* and *Creatine kinase (Ckm)* (Supplementary Fig. 1b, c). We hereafter refer to nuclear translocated MyoD-ER-T7 as MyoD.

To characterize the binding dynamics of nuclear-translocated MyoD during myogenic *trans*-differentiation, we performed time-course analysis using Cleavage Under Targets and Release Using Nuclease (CUT&RUN) with a T7 antibody. We identified induced MyoD binding sites at each time point following 4-OHT treatment, where the signal intensities were >2-fold higher than those at 0 h. Motif analysis confirmed that MyoD was the most significantly enriched motif within induced MyoD binding sites across all time points (Fig. 1c). Concurrently, we performed CUT&RUN with H3K4me1 and H3K27ac antibodies and identified 36,425 induced MyoD binding sites that are marked with H3K4me1 and/or H3K27ac at any time point (Fig. 1d). Unbiased k-means clustering analysis revealed six distinct clusters A-F, each with a unique MyoD binding pattern (Fig. 1e). MyoD binding near muscle cell differentiation and development genes peaked at 24 h or later in clusters D and E. In contrast, early MyoD binding sites in clusters A, B, and C were associated with genes involved in more general cellular processes such as cell-substrate adhesion (Supplementary Fig. 1d). Motif analysis of each cluster revealed strong enrichment of the canonical MyoD motif across all clusters, whereas clusters D-F, which were associated with muscle differentiation genes, also exhibited significant enrichment of MEF2 motifs (Supplementary Fig. 1e).

### Transcriptional outcomes of dynamic MyoD binding

To link MyoD-bound enhancers to transcriptional outcomes, we performed nascent RNA-seq following MyoD nuclear translocation (0, 1, 4, 9, and 24 h). We identified 3,435 differentially expressed genes associated with MyoD-bound enhancers over time. Soft clustering of these differentially expressed genes revealed five dynamic transcriptional patterns: rapid, gradual, and late upregulation (G1-G3), as well as gradual and moderate repression (G4-G5) (Supplementary Fig. 2a). Functional enrichment of these gene groups mirrored dynamic MyoD binding. Rapidly and gradually upregulated gene groups (G1-G2) were enriched for general biological processes such as cell adhesion and actin cytoskeleton organization, whereas the late-upregulated genes (G3) were highly enriched for muscle-specific terms (Supplementary Fig. 2b). Meanwhile, repressed gene groups (G4-G5) were enriched for non-muscle, alternative lineage cell programs including angiogenesis, ureteric bud development, axon guidance, osteoblast differentiation, and immune response, suggesting that MyoD binding represses non-myogenic fate to enforce myogenic reprogramming. At the single gene level, transcription of myogenic marker gene *Myog* and its neighboring gene *Mybph* increased modestly at early time points and significantly at 24 h following MyoD induction (Fig. 1f). When we examined the correlation between MyoD binding and transcriptional dynamics, we observed that enhancers with early and transient MyoD binding (Fig. 1e, clusters A-C) were predominantly located in the genomic vicinity of rapidly upregulated genes but were also moderately associated with gradually repressed genes (Fig. 1g and Supplementary Fig. 2c). In contrast, enhancers where MyoD binding peaked at 24 h or later (Fig. 1e, clusters D-E) were enriched exclusively for upregulated gene groups (Fig. 1g).

We also investigated the temporal dynamics of enhancer histone marks H3K4me1 and H3K27ac on MyoD sites associated with differentially regulated genes. H3K4me1 and H3K27ac exhibited distinct enrichment patterns following MyoD nuclear translocation and binding (Supplementary Fig. 3a, b). Enhancers linked to upregulated genes exhibited features of early or late priming (marked by H3K4me1) as well as activation (marked by H3K27ac). On the other hand, repressed genes exhibited strong enrichment of decommissioned (loss of H3K4me1) and deactivated (loss of H3K27ac) chromatin states following MyoD translocation (Supplementary Fig. 3c).

On the *Myog* locus, MyoD binding was significant only after 24 h of 4-OHT treatment, whereas on the surrounding regions, MyoD binding appeared as early as at 1 h. These MyoD^+^ regions were heavily marked by H3K4me1 and/or H3K27ac, and increased transcriptional output was observed around 9 h after MyoD binding (Fig. 1h). These results suggest that MyoD targets distinct enhancers in a temporally regulated manner during myogenic reprogramming of preadipocytes.

### Acutely translocated MyoD colocalizes with chromatin modifiers on enhancers

**Fig. 2:**
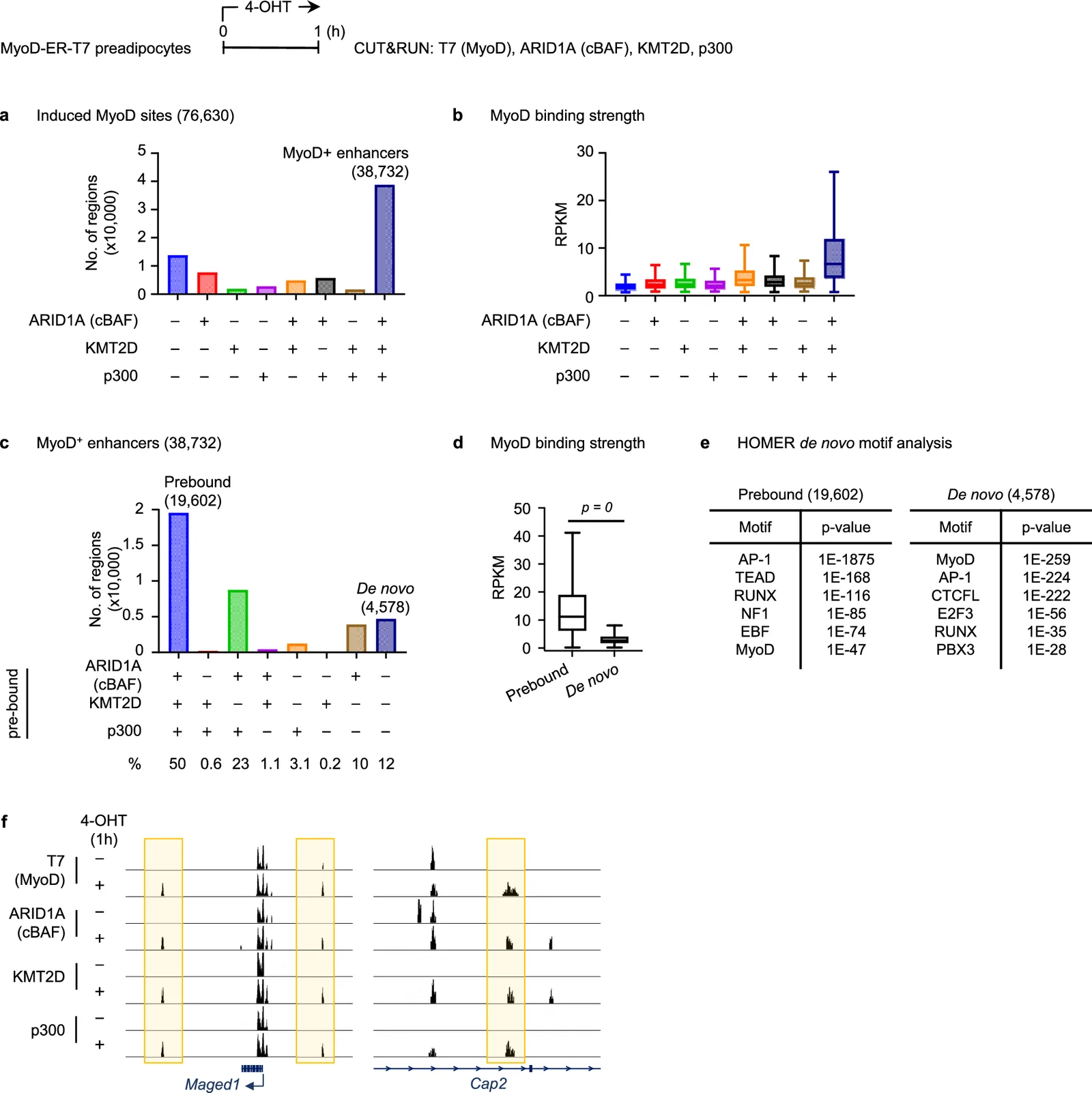
Acutely translocated MyoD colocalizes with chromatin modifiers cBAF, KMT2D, and p300 on enhancers. MyoD-ER-T7 expressing preadipocytes were treated with 4-OHT for 1 h, followed by CUT&RUN analysis. **a** Bar chart showing ARID1A (an exclusive subunit of cBAF), KMT2D, and p300 binding status on induced MyoD sites. **b** Box plots displaying the normalized MyoD read counts in subgroups defined in (**a**). The number of genomic regions (*n*) for each category from left to right is: 13,745, 7567, 1838, 2669, 4779, 5631, 1579, and 38,732. **c** Bar chart showing ARID1A (cBAF), KMT2D, and p300 binding on 38,732 MyoD^+^ enhancers defined in (**a**) prior to 4-OHT treatment. **d** Box plots showing the normalized MyoD read counts on cBAF-KMT2D-p300 prebound or de novo sites defined in (**c**). The number of genomic regions (*n*) is 19,602 for the prebound group and 4578 for the de novo group. Statistical significance was determined using a two-sided, unpaired Mann Whitney test. **e** HOMER de novo motif analysis on sites defined in (**c**). *P* values for motif enrichment were determined by a one-sided hypergeometric test without adjustment for multiple comparisons. **f** Genome browser view of MyoD binding sites around *Maged1* and *Cap2* loci. For all box plots, the boxes represent the first and third quartiles with the horizontal line showing the median, and whiskers indicating minimum and maximum. Source data are provided as a Source Data file.

Because the majority of MyoD-ER is translocated into the nucleus within 1 h of 4-OHT treatment (Fig. 1b), we focused on induced MyoD sites at 1 h to investigate the early binding of MyoD and chromatin modifiers. We examined the genomic enrichment of KMT2D, cBAF, and p300 on MyoD binding sites, using CUT&RUN with antibodies against T7 tag (for MyoD), ARID1A (a representative and exclusive subunit of cBAF), KMT2D, and p300. Among the 76,630 induced MyoD binding sites, ~51% (38,732 sites) were co-occupied by all three chromatin modifiers KMT2D, cBAF, and p300 (Fig. 2a). The strength of MyoD binding on these 38,732 sites was significantly higher than on any other subset of sites (Fig. 2b). Because cBAF, KMT2D, and p300 are well-established enhancer regulators, we assigned this group as MyoD⁺ enhancers. For subsequent analysis, we focused on these 38,732 MyoD^+^ enhancers.

Prior to MyoD recruitment, the majority of the 38,732 MyoD^+^ enhancers were prebound by at least one of the three chromatin modifiers, with 50% (19,602 sites) prebound by all three. In contrast, approximately 12% (4578 sites) showed de novo binding of cBAF, KMT2D, and p300 upon MyoD recruitment (Fig. 2c). MyoD binding was significantly stronger on cBAF-KMT2D-p300 prebound sites compared to the de novo sites (Fig. 2d). Although the MyoD motif was enriched in both groups of sites, it was the most significantly enriched motif on the de novo sites, whereas the AP-1 motif ranked highest on prebound sites (Fig. 2e-f).

### Acute KMT2D depletion disrupts de novo binding of MyoD, cBAF, and p300 on enhancers

**Fig. 3:**
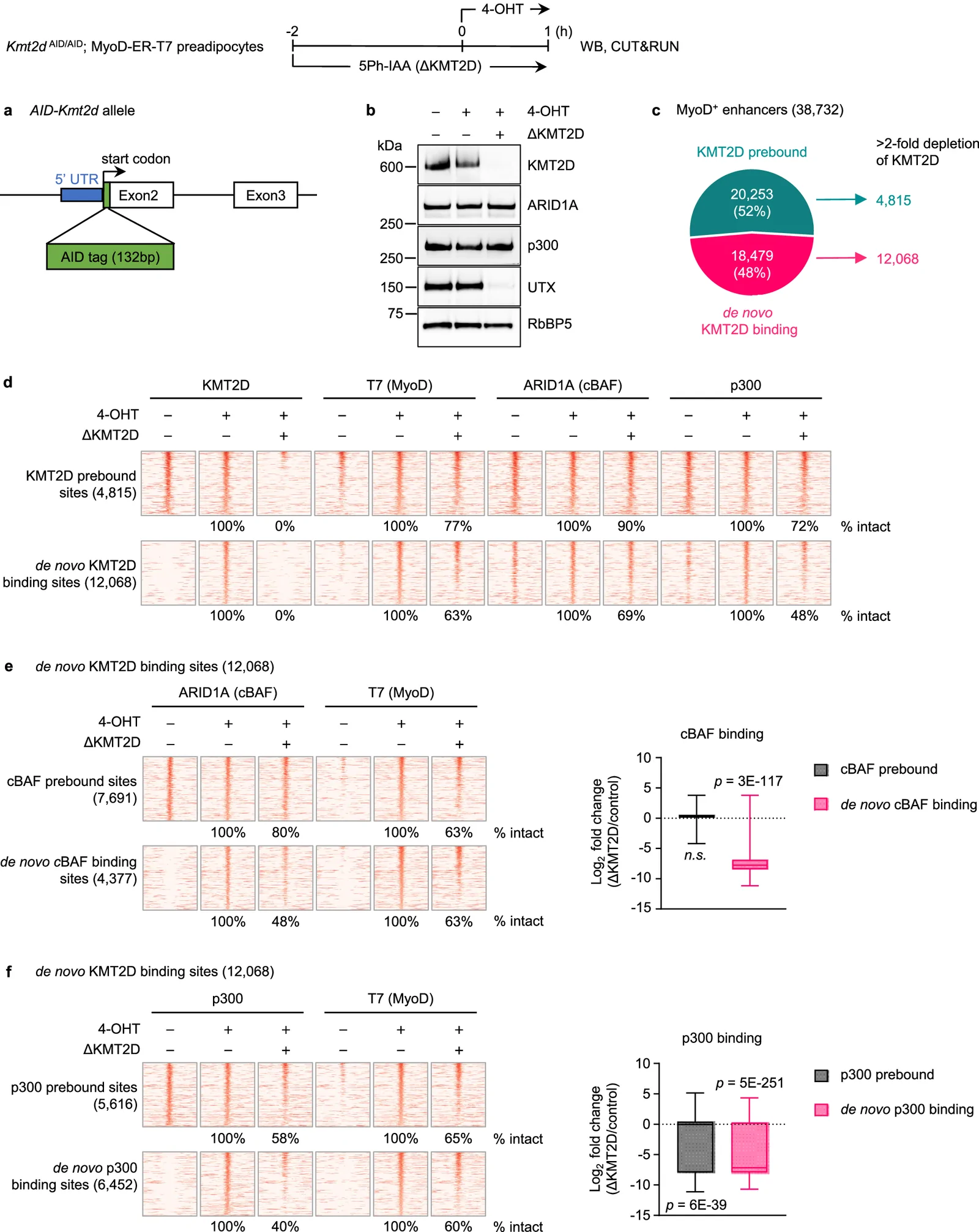
Acute KMT2D depletion disrupts de novo binding of MyoD, cBAF, and p300 on enhancers. **a** Schematic for generating the knockin allele encoding AID-tagged KMT2D. *Kmt2d* ^AID/AID^; MyoD-ER-T7 preadipocytes were pretreated with 5Ph-IAA (ΔKMT2D) for 2 h, and then 4-OHT was added for 1 h to induce MyoD nuclear translocation. Cells were harvested for WB and CUT&RUN analysis. **b** WB of nuclear extracts for KMT2D, ARID1A (cBAF), p300, and UTX. Antibodies used were indicated on the right. RbBP5 was used as a sample-processing control across all replicate membranes, and representative signals are shown. This experiment was repeated independently at least twice with similar results. **c** Pie chart illustrating KMT2D binding status on 38,732 MyoD^+^ enhancers. **d** Heat maps for CUT&RUN of KMT2D, T7 (MyoD), ARID1A (cBAF), and p300 on KMT2D prebound or de novo KMT2D binding sites with >2-fold depletion of KMT2D as defined in (**c**). **e**, **f** Heat maps (left panel) and box plots (right panel) for CUT&RUN data on 12,068 MyoD^+^ enhancers with de novo KMT2D binding. Enhancers are categorized based on cBAF binding (**e**) or p300 binding (**f**) before and after 4-OHT treatment. All heat maps span ± 3 kb around MyoD binding sites, and sites were ranked by the intensity of MyoD (T7) in the 4OHT-treated control. Box plots show fold changes of cBAF binding intensity (**e**) or p300 binding intensity (**f**) between KMT2D-depleted (ΔKMT2D) and control samples. The number of genomic regions (*n*) in (**e**) is 7691 for the cBAF-prebound group and 4377 for the de novo cBAF binding group. The number of genomic regions (*n*) in (**f**) is 5616 for the p300-prebound group and 6452 for the de novo p300 binding group. Statistical significance for box plots was determined using a one-sided Wilcoxon signed-rank test. For all box plots, the boxes represent the first and third quartiles, the horizontal line shows the median, and whiskers indicate minimum and maximum. Source data are provided as a Source Data file.

To investigate the roles of KMT2D in regulating MyoD, cBAF, and p300 binding on enhancers, we utilized the auxin-inducible degron 2 (AID2) system to acutely deplete KMT2D, the largest nuclear protein in mammalian cells^9,21^. For this purpose, *Kmt2d* ^AID/AID^ mice expressing AID-tagged KMT2D protein were generated using CRISPR-mediated insertion of an AID tag to the N-terminus of *Kmt2d* alleles (Fig. 3a, Supplementary Fig. 4a). We then crossed *Kmt2d* ^AID/AID^ mice with *Kmt2c* ^f/f^; *Cre-ER* mice^22^ to obtain primary *Kmt2c* ^f/f^; *Kmt2d* ^AID/AID^; *Cre-ER* preadipocytes (Supplementary Fig. 4b). After immortalization, cells were treated with 4-OHT to permanently delete the *Kmt2c* gene by Cre recombinase to prevent the potential functional compensation by KMT2C^2^. The resulting *Kmt2c* ^-/-^; *Kmt2d* ^AID/AID^ cells were infected with retroviruses expressing F74G-mutant *Oryza sativa* TIR1 (OsTIR1-F74G) and MyoD-ER-T7 (Supplementary Fig. 4c,d). Following 5Ph-IAA treatment, KMT2D was depleted within 2 h, which was also confirmed by the loss of UTX, whose stability depends on KMT2D (Supplementary Fig. 4e)^23^. We refer to these cells as *Kmt2d* ^AID/AID^; MyoD-ER-T7.

*Kmt2d* ^AID/AID^; MyoD-ER-T7 cells were pretreated with 5Ph-IAA for 2 h, and then 4-OHT was added for 1 h to induce MyoD nuclear translocation. As shown in Fig. 3b, 3 h of 5Ph-IAA treatment significantly depleted KMT2D and UTX without much effect on ARID1A and p300 protein levels. To examine how acute loss of KMT2D affects MyoD, cBAF, and p300 binding, we performed CUT&RUN. On the 38,732 MyoD^+^ enhancers (Fig. 2a), we observed that 52% (20,253 sites) were prebound by KMT2D, while the remaining 48% (18,479 sites) showed de novo KMT2D binding. We focused on 4815 KMT2D prebound sites and 12,068 de novo KMT2D binding sites where KMT2D signal intensity was reduced >2-fold upon depletion (Fig. 3c). On KMT2D prebound sites, the loss of KMT2D did not affect MyoD and p300 binding on >70% of the sites, and 90% of cBAF binding sites were also intact. However, KMT2D depletion had a more significant impact on de novo KMT2D binding sites. 37% of these sites lost MyoD binding, whereas 31% and 52% showed reduced cBAF and p300 binding, respectively (Fig. 3d).

The 12,068 de novo KMT2D binding sites were further categorized according to cBAF or p300 binding status prior to MyoD translocation, as we observed that many of these sites were pre-marked by either of these factors. On cBAF- or p300-prebound sites, KMT2D depletion reduced cBAF or p300 binding on 20% or 42% of them, respectively. On sites with de novo cBAF or p300 binding, however, the loss of KMT2D reduced cBAF or p300 binding on 52% or 60% of them, respectively (Fig. 3e-f). Together, these results indicate that KMT2D is important for de novo binding of MyoD, cBAF, and p300 on enhancers.

### cBAF activity is required for de novo binding of MyoD and chromatin modifiers on enhancers

**Fig. 4:**
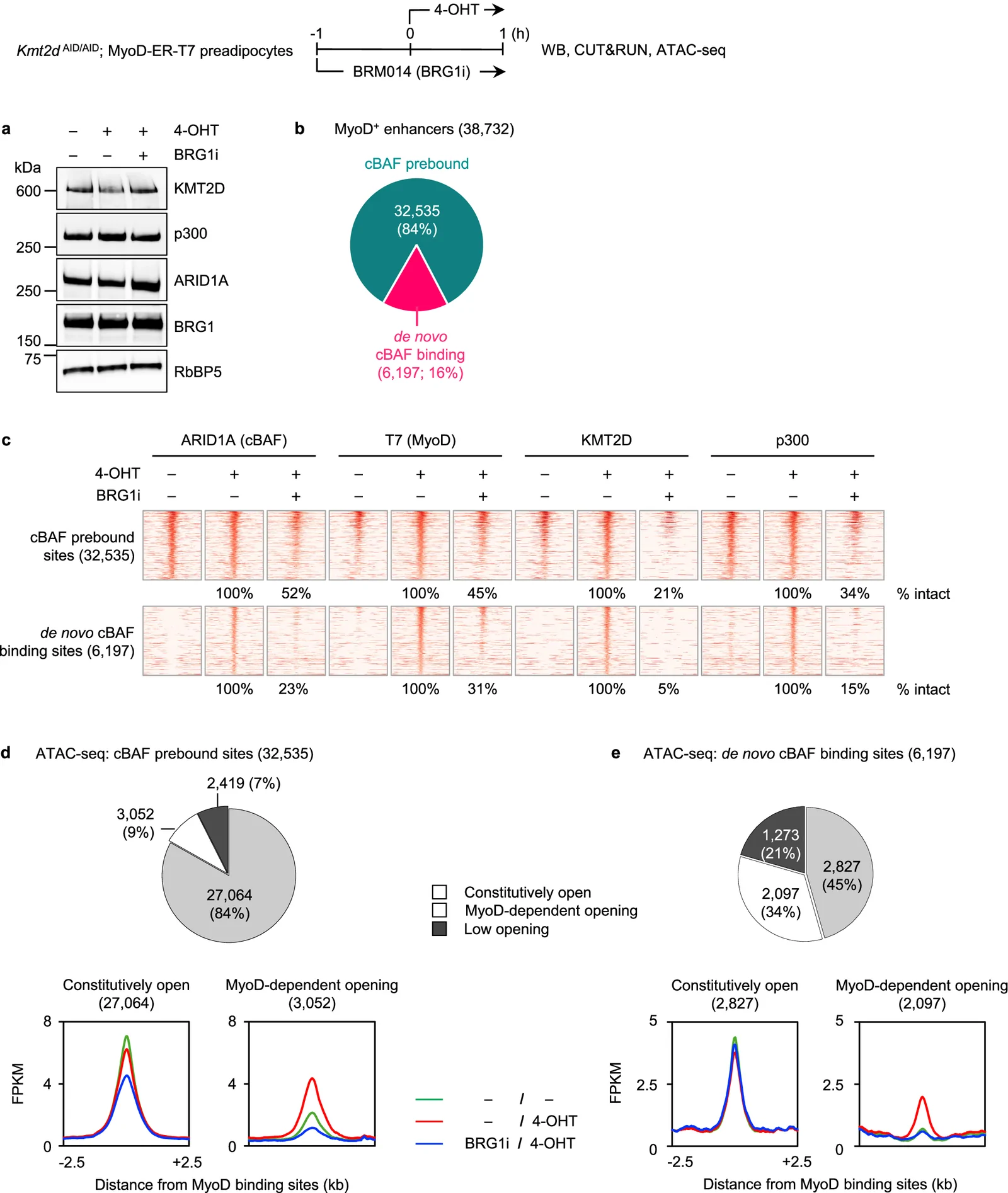
Inhibition of cBAF activity markedly reduces de novo binding of MyoD, KMT2D, cBAF, and p300 on enhancers. *Kmt2d* ^AID/AID^; MyoD-ER-T7 preadipocytes were pretreated with BRG1 inhibitor BRM014 (BRG1i) for 1 h, and then 4-OHT was added for 1 h to induce MyoD nuclear translocation. Cells were harvested for WB, CUT&RUN, and ATAC-seq. **a** WB of nuclear extracts for KMT2D, p300, and cBAF subunits BRG1 and ARID1A. RbBP5 was used as a sample-processing control across all replicate membranes, and representative signals are shown. This experiment was repeated independently at least twice with similar results. **b** Pie chart illustrating cBAF binding status on 38,732 MyoD^+^ enhancers. **c** Heat maps for CUT&RUN of ARID1A (cBAF), T7 (MyoD), KMT2D, and p300 on cBAF pre-bound and de novo cBAF binding sites. **d**, **e** Chromatin accessibility determined by ATAC-seq signals on MyoD^+^ enhancers. Chromatin accessibility status on cBAF prebound sites (**d**) or de novo cBAF binding sites (**e**) is shown in pie charts (*upper panels*). Average profiles of normalized ATAC-seq reads on constitutively open and MyoD-dependent opening sites are shown in *lower panels*.

Next, we addressed the role of cBAF in modulating the early binding of MyoD and chromatin modifiers on enhancers. We utilized BRM014 (BRG1i), a catalytic inhibitor of the cBAF enzymatic subunit BRG1^24,25^. We pretreated *Kmt2d* ^AID/AID^; MyoD-ER-T7 cells with BRG1i for 1 h and then induced MyoD nuclear translocation with 4-OHT for 1 h. BRG1i treatment did not alter the protein levels of BRG1, ARID1A, KMT2D, or p300 (Fig. 4a).

We performed CUT&RUN to assess the effects of BRG1 inhibition on MyoD, cBAF, KMT2D, and p300 chromatin binding. On the 38,732 MyoD^+^ enhancers (Fig. 2a), the vast majority (32,535) were prebound by cBAF, while 16% (6,197) showed de novo cBAF binding (Fig. 4b). On cBAF prebound sites, BRG1i treatment led to a >2-fold decrease in MyoD binding on 55% of them. Binding of all chromatin modifiers was also broadly reduced. cBAF, KMT2D, and p300 binding showed >2-fold reduction on 48%, 79%, and 66% of the prebound sites, respectively (Fig. 4c). The impact was even more pronounced on de novo cBAF binding sites, where the loss of BRG1 activity diminished MyoD and cBAF binding on 69% and 77% of sites and abolished KMT2D and p300 binding on 95% and 85% of them, respectively (Fig. 4c).

To investigate chromatin accessibility on MyoD^+^ enhancers, we performed ATAC-seq and compared the signals before and after MyoD nuclear translocation. We identified three groups with distinct characteristics: constitutively open, MyoD-dependent opening, and low opening. Among 32,535 cBAF prebound enhancers, the majority (84%; 27,064 sites) were constitutively open, while 9% (3052 sites) and 7% (2419 sites) belonged to the MyoD-dependent opening or low opening group, respectively (Fig. 4d, *upper panel*). In contrast, a significant number (34%; 2097 sites) of de novo cBAF binding sites showed MyoD-dependent opening, while 45% and 21% were constitutively or lowly open, respectively (Fig. 4e, *upper panel*). We then examined changes in chromatin accessibility upon BRG1i treatment. On cBAF prebound enhancers, inhibition of BRG1 activity led to a more drastic decrease in chromatin accessibility on 3052 MyoD-dependent opening sites, compared to that on the 27,064 constitutively open sites (Fig. 4d, *lower panel*). On de novo cBAF binding sites, BRG1 inhibition did not alter chromatin accessibility on constitutively open sites, but completely abolished MyoD-mediated chromatin opening (Fig. 4e, *lower panel*). These results suggest that MyoD-dependent chromatin opening depends on BRG1 enzymatic activity. Our data also indicate that BRG1 activity is required for de novo binding of MyoD, cBAF complex itself, and other chromatin modifiers KMT2D and p300 on enhancers.

### De novo binding of MyoD and chromatin modifiers depends on p300 acetyltransferase activity

**Fig. 5:**
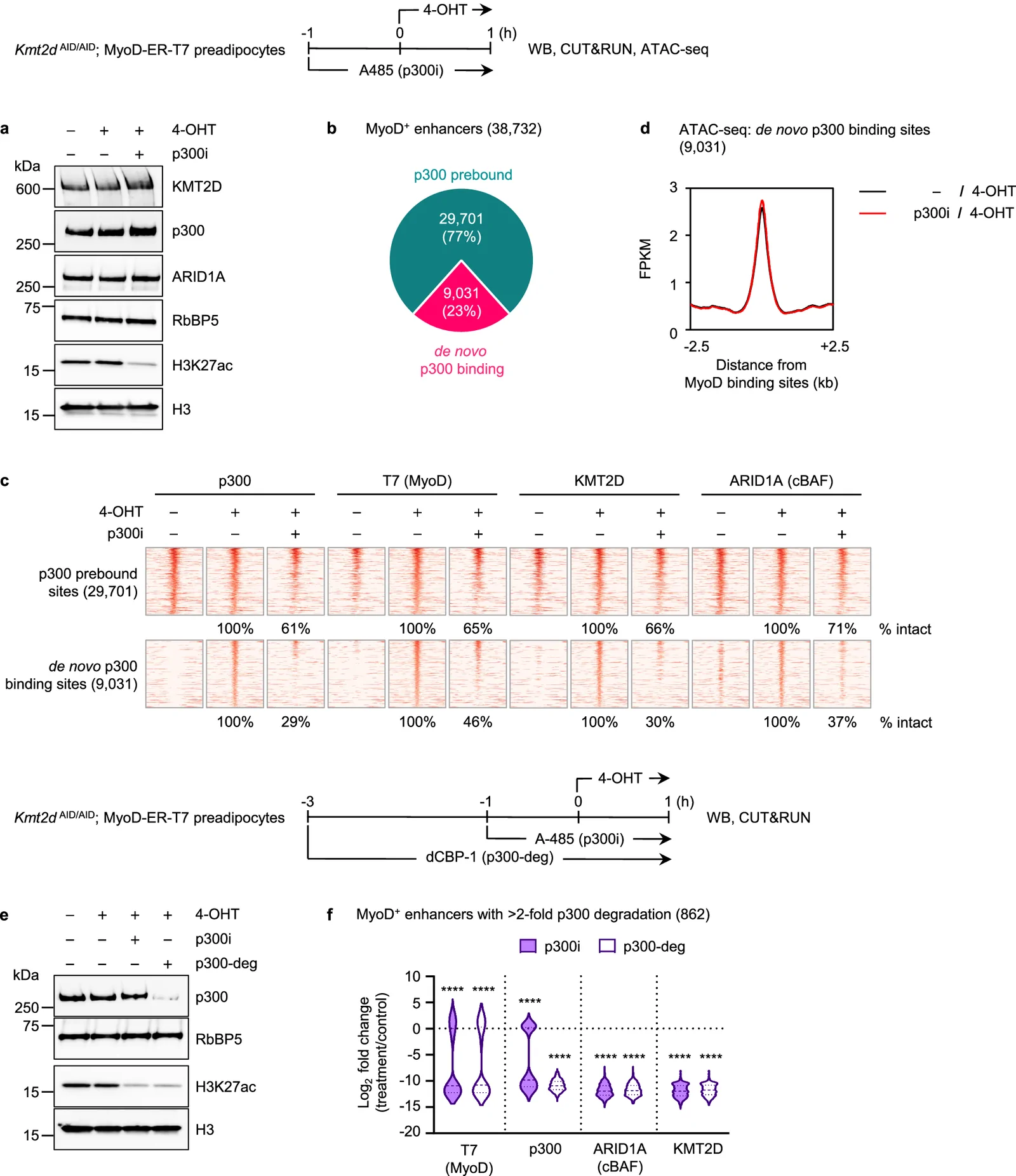
De novo binding of MyoD and chromatin modifiers on enhancers depends on p300 acetyltransferase activity. **a–d** *Kmt2d* ^AID/AID^; MyoD-ER-T7 preadipocytes were pretreated with p300/CBP inhibitor A-485 (p300i) for 1 h, and then 4-OHT was added for 1 h to induce MyoD nuclear translocation. Cells were collected for WB, CUT&RUN, and ATAC-seq. **a** WB of nuclear extracts for p300, KMT2D, ARID1A (cBAF) and histone extracts for H3K27ac. RbBP5 and H3 were used as sample-processing controls across all replicate membranes, and representative signals are shown. This experiment was repeated independently at least twice with similar results. **b** Pie chart illustrating p300 binding status on 38,732 MyoD^+^ enhancers. **c** Heat maps for CUT&RUN of p300, T7 (MyoD), ARID1A (cBAF), and KMT2D on p300 prebound and de novo p300 binding sites. Heat maps span ± 3 kb around MyoD binding sites, and sites are ranked by the intensity of T7 (MyoD) in the 4OHT-treated control. **d** Average profiles of normalized ATAC-seq reads on 9,031 de novo p300 binding sites with or without p300i treatment. **e**, **f** *Kmt2d* ^AID/AID^; MyoD-ER-T7 preadipocytes were pretreated with p300/CBP degrader dCBP-1 (p300-deg) for 3 h or p300i for 1 h. Then, 4-OHT was added for 1 h to induce MyoD nuclear translocation. **e** WB of nuclear extracts for p300 or histone extracts for H3K27ac. RbBP5 and H3 were used as sample-processing controls. This experiment was repeated independently at least twice with similar results. **f** Violin plot illustrating changes in binding of T7 (MyoD), p300, ARID1A (cBAF), and KMT2D upon p300i or p300-deg treatment. The analysis was performed on MyoD^+^ enhancers with >2-fold reduced p300 binding upon p300-deg. Statistical significance was determined using a one-sided Wilcoxon signed-rank test (****p < 0.0001). Exact P values for the comparisons shown, from left to right, are 3.24E-25, 2.16E-17, 4.24E-45, 3.25E-260, 9.86E-175, 1.08E-127, 8.58E-118, and 1.84E-160. Source data are provided as a Source Data file.

To understand how p300 acetyltransferase activity influences MyoD and chromatin modifier binding on enhancers, we used a p300 catalytic inhibitor A485 (p300i)^26^. *Kmt2d* ^AID/AID^; MyoD-ER-T7 cells were pretreated with p300i for 1 h, followed by 4-OHT treatment to induce MyoD nuclear translocation for 1 h. p300i treatment did not affect protein levels of p300, KMT2D, and ARID1A, but significantly reduced global H3K27ac levels (Fig. 5a).

We performed CUT&RUN to examine MyoD, cBAF, KMT2D, and p300 binding on enhancers upon p300i treatment. Among the 38,732 MyoD^+^ enhancers defined in Fig. 2a, 77% (29,701 sites) were prebound by p300, and 23% (9031 sites) showed de novo p300 binding (Fig. 5b). p300i treatment reduced MyoD, p300, KMT2D, and cBAF binding on approximately 30–40% of p300 prebound sites. In contrast, de novo p300 binding sites were more sensitive to p300 inhibition: MyoD enrichment was decreased on 54% of these sites, whereas p300, KMT2D, and cBAF binding was reduced on ~70% of de novo p300 binding sites (Fig. 5c). The loss of MyoD and chromatin modifier enrichment was not due to changes in chromatin accessibility upon p300 inhibition (Fig. 5d). These data suggest that p300 might play a more dominant role than previously reported in modulating the binding of LDTFs and chromatin modifiers on enhancers.

We further validated our observations using a heterobifunctional degrader, dCBP-1 (p300-deg), to acutely deplete p300 protein^27^. *Kmt2d* ^AID/AID^; MyoD-ER-T7 cells were pretreated with either p300-deg (3 h) or p300i (1 h), before MyoD nuclear translocation was induced by 4-OHT (1 h). While p300-deg treatment significantly reduced p300 protein levels, p300i treatment did not. However, global H3K27ac levels were decreased by both treatments (Fig. 5e). CUT&RUN analysis revealed that p300-deg and p300i treatment reduced MyoD, cBAF, and KMT2D binding on MyoD^+^ enhancers to a similar extent (Fig. 5f). Together, these results indicate that p300, through its acetyltransferase activity, is essential for de novo binding of MyoD, cBAF, KMT2D, and p300 on enhancers during early stages of myogenic reprogramming.

We also examined the effects of p300 inhibition on endogenous MyoD binding in C2C12 myoblasts, where de novo MyoD binding is induced upon differentiation^17^. C2C12 myoblasts were subjected to 2 h or 24 h of myogenesis, with p300i applied for 2 h prior to analysis (Supplementary Fig. 5a). As expected, a 2 h p300i treatment markedly reduced global H3K27ac levels (Supplementary Fig. 5b). After 2 h and 24 h of differentiation, we identified 718 and 3,075 de novo MyoD^+^ p300^+^ sites, respectively. MyoD motifs were ranked among the most significantly enriched ones (Supplementary Fig. 5c and e). At both time points, inhibition of p300 acetyltransferase activity significantly reduced de novo binding of MyoD and p300 on de novo MyoD^+^ p300^+^ sites (Supplementary Fig. 5d, f). These findings are consistent with our observations in preadipocytes and demonstrate that p300 enzymatic activity is essential for MyoD and p300 binding on enhancers.

### Acetylation of MyoD is required for chromatin modifier binding on MyoD^+^ enhancers

CBP/p300 has been implicated in the acetylation of MyoD in vitro^28^. To examine if the genomic binding of MyoD and chromatin modifiers relies on the acetylation of MyoD itself, we mutated three lysine residues (K99, K102, and K104) of the 4-OHT inducible MyoD-ER-T7 to arginines (R), rendering the protein non-acetylatable by acetyltransferases (Supplementary Fig. 6a)^29^. We refer to this mutant as MyoD-K/R, in contrast to the wild-type, MyoD-WT.

We infected *Kmt2c*^−/−^; *Kmt2d* ^AID/AID^ preadipocytes (Supplementary Fig. 4c) with retroviruses expressing either MyoD-WT or MyoD-K/R. MyoD-K/R could not drive myogenesis as efficiently as MyoD-WT, as assessed by both cell morphology and marker gene induction (Supplementary Fig. 6b, c). Consistent with a previous report^30^, MyoD-K/R was expressed at comparable levels and translocated to the nucleus upon 4-OHT treatment at similar rates as MyoD-WT (Supplementary Fig. 6d). Protein levels of KMT2D, p300, and ARID1A were also comparable between MyoD-WT and MyoD-K/R samples.

To test if K/R mutations affect MyoD chromatin binding, we induced nuclear translocation of MyoD-WT and MyoD-K/R with 4-OHT for 1 h and performed ChIP-seq analysis. Of the 16,090 MyoD^+^ enhancers identified in MyoD-WT expressing cells, 10,305 sites were prebound by all three chromatin modifiers KMT2D, cBAF, and p300, while 1938 sites were de novo for all three modifiers. K/R mutations did not impact MyoD binding on either prebound or de novo sites. However, loss of MyoD acetylation significantly reduced KMT2D and cBAF binding on prebound sites. Moreover, on de novo sites, non-acetylatable mutations significantly compromised the binding of all three modifiers, KMT2D, cBAF, and p300 (Supplementary Fig. 6e, f). These results suggest that while MyoD acetylation is dispensable for its own DNA binding, it promotes the recruitment and/or maintenance of chromatin modifiers on MyoD^+^ enhancers.

### Cooperative binding of KMT2D, cBAF, and p300 on MyoD-intact enhancers

**Fig. 6:**
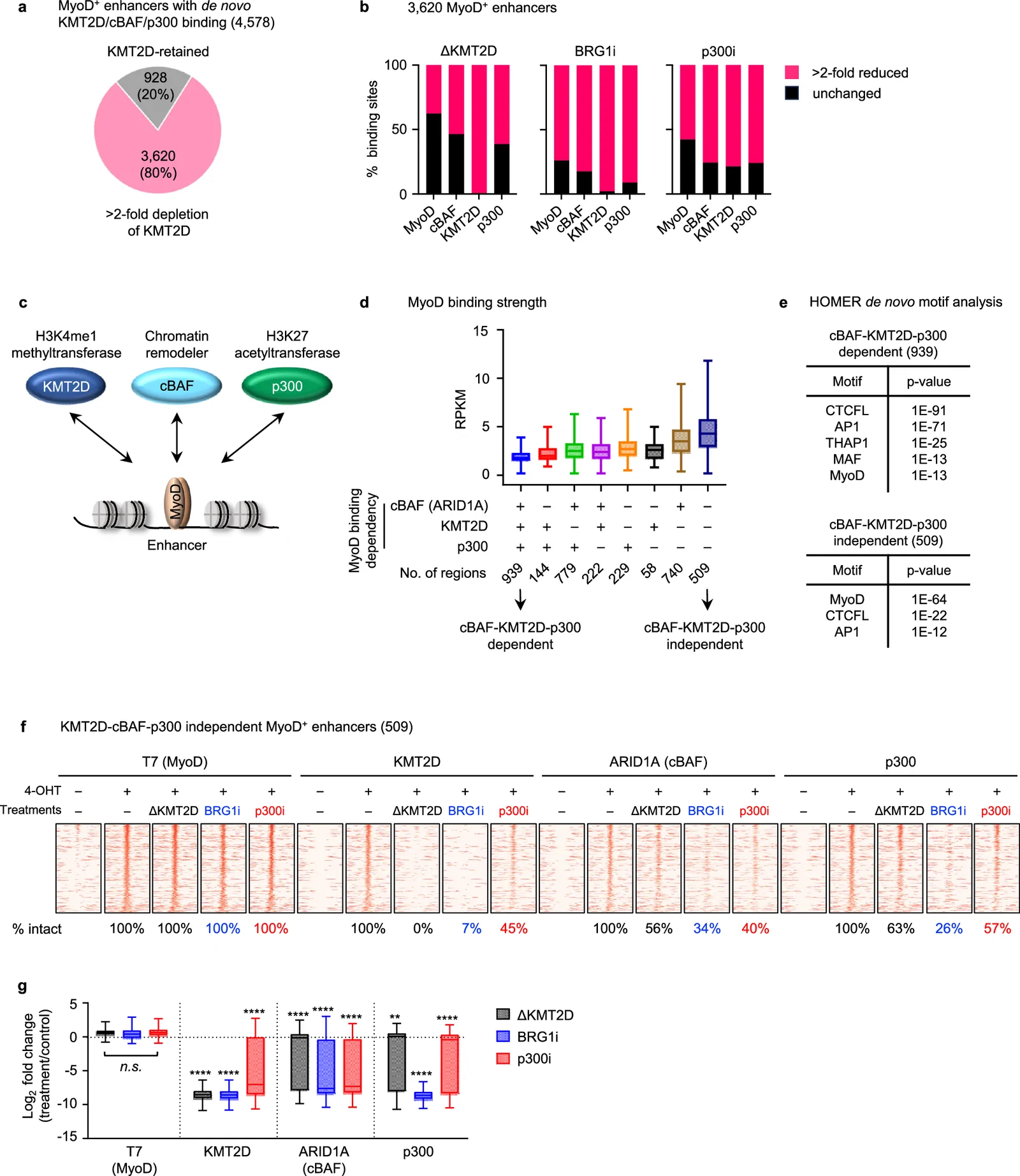
Cooperative binding of chromatin modifiers on MyoD-intact enhancers. **a** Pie chart illustrating KMT2D protein depletion on 4,578 MyoD^+^ enhancers with de novo binding of KMT2D, cBAF, and p300 defined Fig. 2c. **b** Bar graphs illustrating effects of ΔKMT2D, BRG1i, and p300i on MyoD^+^ enhancers with de novo binding of KMT2D, cBAF, and p300. **c** A model depicting the interdependent relationship between myogenic TF MyoD and chromatin modifiers KMT2D, cBAF, p300 on enhancers. **d** Box plots showing the normalized MyoD read counts on 3,620 MyoD-bound enhancers, grouped by the presence of MyoD binding decrease upon chromatin modifier interventions (MyoD binding dependency). The number of genomic regions (*n*) for each category from left to right is: 939, 144, 779, 222, 229, 58, 740, and 509. **e** HOMER de novo motif analysis on cBAF-KMT2D-p300 dependent and independent sites defined in (**a**). **f** Heat maps illustrating changes in binding of T7 (MyoD), KMT2D, ARID1A (cBAF), and p300 on 509 cBAF-KMT2D-p300 independent MyoD^+^ enhancers defined in (**d**). Heat maps span ±3 kb around MyoD binding sites, and sites are ranked by the intensity of T7 (MyoD) in the 4OHT control. **g** Box plots corresponding to the heat maps in (**f**), showing binding changes for each factor (*n* = 509 genomic regions). Statistical significance was determined using a one-sided Wilcoxon signed-rank test (*n.s*. not significant; **p < 0.01; ****p < 0.0001). For the significant comparisons shown, the exact P values from left to right are 5.97E-154, 3.01E-107, 7.51E-47, 4.68E-11, 6.79E-62, 3.79E-82, 1.75E-03, 7.76E-54, and 2.49E-13. For all box plots, the boxes represent the first and third quartiles, the horizontal line shows the median, and whiskers indicate the minimum and maximum. Source data are provided as a Source Data file.

Our results from individual acute perturbations suggest that KMT2D, cBAF, and p300 are important for de novo MyoD binding on enhancers. To directly compare the effects of KMT2D depletion, BRG1 or p300 inhibition, we focused on a common set of 3,620 MyoD^+^ enhancers that showed de novo binding of all three chromatin modifiers and exhibited >2-fold reduction of KMT2D signal upon its depletion (Fig. 6a). All three interventions markedly reduced de novo MyoD binding on enhancers, with BRG1 inhibition having the most pronounced impact, followed by p300 inhibition and KMT2D depletion. Acute depletion or inhibition of one chromatin modifier markedly reduced the binding of the other two on MyoD^+^ enhancers (Fig. 6b-c).

Based on the motif analysis of the broader set of 4,578 de novo sites (Fig. 2e), of which these 3,620 KMT2D-depleted MyoD^+^ enhancers are the major subset (Fig. 6a), MyoD is likely the driving force of chromatin modifier recruitment. Within this set, we further identified two subgroups of sites. One shows >2-fold reduced MyoD binding upon all three perturbations (939 cBAF-KMT2D-p300 dependent sites). The other shows intact MyoD binding under all conditions (509 cBAF-KMT2D-p300 independent sites) (Fig. 6d). MyoD binding was significantly stronger on independent sites. HOMER motif analysis showed that canonical MyoD motifs were most significantly enriched on independent sites. In contrast, dependent sites exhibited strong enrichment of CTCFL and AP1 motifs, while MyoD motifs ranked lower, suggesting indirect binding (Fig. 6e).

To distinguish direct effects on chromatin modifier enrichment from those secondary to reduced MyoD binding, we focused on 509 cBAF-KMT2D-p300 independent MyoD^+^ enhancers. On these 509 enhancers, KMT2D binding was diminished on 93% and 55% of them by BRG1i and p300i treatments, respectively. cBAF binding was reduced by KMT2D depletion, BRG1 or p300 inhibition on 44%, 66%, and 60% of sites, respectively. Similarly, p300 binding showed a > 2-fold decrease on 37%, 74%, and 43% of sites, upon the loss of KMT2D protein, BRG1 or p300 catalytic activity, respectively (Fig. 6f-g). Interdependent binding of chromatin modifiers was also observed in a broader set of MyoD^+^ enhancers where MyoD binding remained unaffected by individual treatments (Supplementary Fig. 7).

### GR displays interdependencies with chromatin modifiers on enhancers

**Fig. 7:**
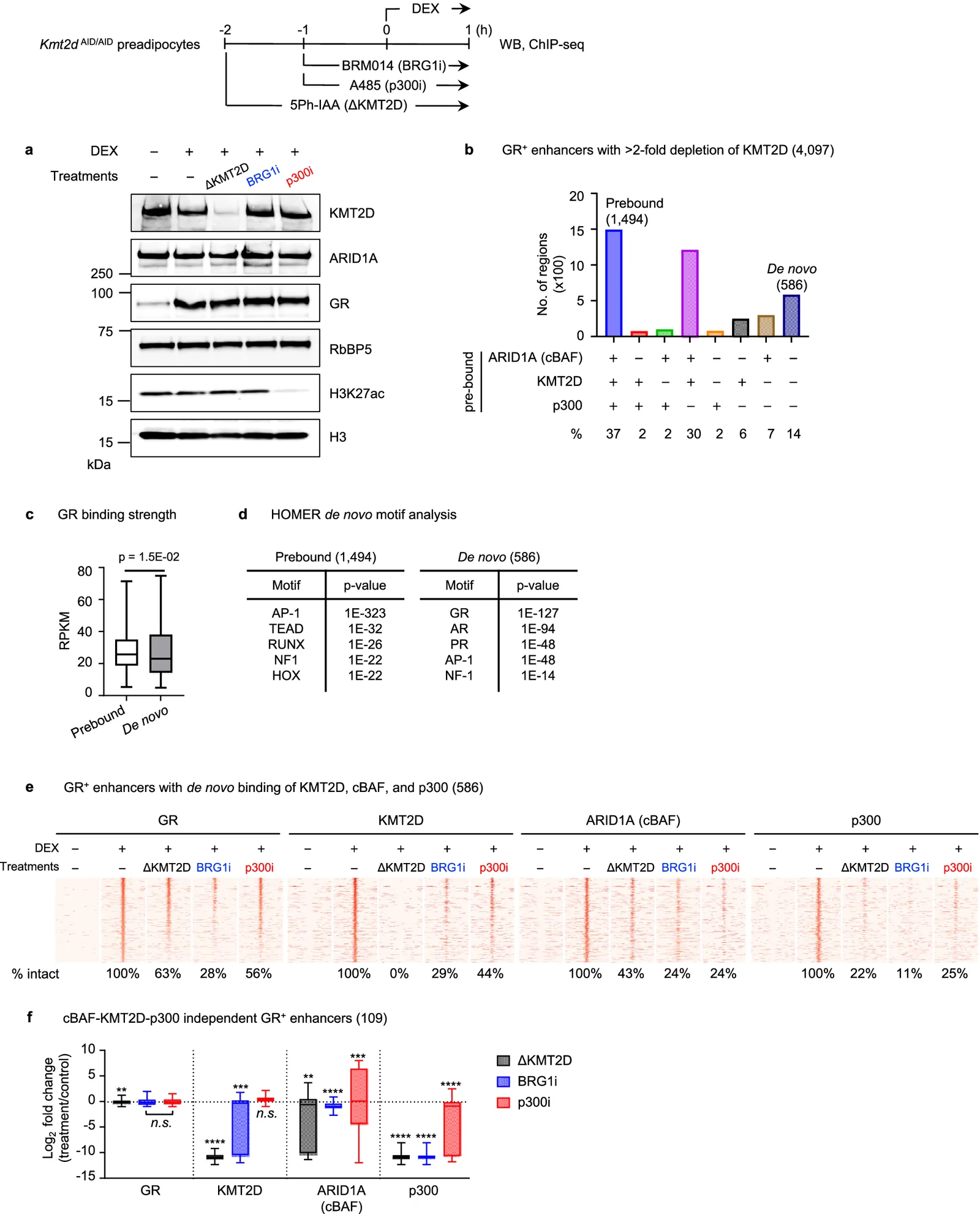
DEX-activated GR displays interdependencies with chromatin modifiers KMT2D, cBAF, and p300 on enhancers. *Kmt2d* ^AID/AID^ preadipocytes were pretreated with 5Ph-IAA (ΔKMT2D) for 2 h, BRG1i or p300i for 1 h, and then 100 nM DEX was added for 1 h to induce GR nuclear translocation. Cells were collected for WB and ChIP-seq. **a** WB of nuclear extracts for KMT2D, ARID1A (cBAF), and GR and histone extracts for H3K27ac. RbBP5 and H3 were used as sample-processing controls across all replicate membranes, and representative signals are shown. This experiment was repeated independently at least twice with similar results. **b** Bar chart showing ARID1A (cBAF), KMT2D, and p300 binding on 4097 GR^+^ enhancers with >2-fold KMT2D depletion, prior to DEX treatment. **c** Box plots showing the normalized GR read counts on GR^+^ enhancers with cBAF-KMT2D-p300 prebound or de novo sites defined in (**b**). The number of genomic regions (*n*) is 1494 for the prebound group and 586 for the de novo group. Statistical significance was determined using a two-sided, unpaired Mann-Whitney test. **d** HOMER de novo motif analysis on sites defined in (**b**). **e** Heat maps for ChIP-seq of GR, KMT2D, ARID1A (cBAF), and p300 on GR^+^ enhancers with de novo binding of cBAF, KMT2D, and p300. Heat maps span ±3 kb around GR binding sites, and sites are ranked by the intensity of GR in the DEX-treated control. **f** Box plots illustrating changes in binding of GR, KMT2D, ARID1A (cBAF), and p300 on cBAF-KMT2D-p300-independent GR^+^ enhancers (*n* = 109 genomic regions). Statistical significance was determined using a one-sided Wilcoxon signed-rank test (*n.s*. not significant; **p < 0.01; ***p < 0.001; ****p < 0.0001). For the significant comparisons shown, the exact P values from left to right are 5.69E-03, 1.54E-33, 3.78E-04, 2.83E-03, 1.21E-25, 4.31E-04, 6.11E-16, 2.51E-19, and 2.63E-14. For all box plots, the boxes represent the first and third quartiles, the horizontal line shows the median, and whiskers indicate minimum and maximum. Source data are provided as a Source Data file.

To determine whether the dynamics observed between MyoD and chromatin modifiers extend to other TFs, we examined the relationships among the SDTF GR and chromatin modifiers KMT2D, cBAF, and p300 following the treatment of synthetic glucocorticoid Dexamethasone (DEX). *Kmt2d* ^AID/AID^ cells were pretreated with 5Ph-IAA for 2 h, or with Brg1i or p300i for 1 h, and subsequently treated with DEX for 1 h to induce GR nuclear translocation. As expected, KMT2D depletion or p300 inhibition markedly reduced KMT2D or H3K27ac levels, respectively, whereas protein levels of p300, KMT2D, and ARID1A, as well as GR nuclear translocation, remained unchanged in all conditions (Fig. 7a).

We performed ChIP-seq to examine GR, cBAF, KMT2D, and p300 binding following DEX treatment and acute interventions. We identified 4097 GR^+^ enhancers for analysis, based on the following criteria: (1) GR binding is >2-fold induced upon DEX treatment; (2) cBAF, KMT2D, and p300 are enriched; and (3) KMT2D signal is >2-fold decreased upon depletion. Among 4097 GR^+^ enhancers, 1494 sites (37%) were prebound by cBAF, KMT2D, and p300, while 586 sites (14%) were de novo for all three modifiers (Fig. 7b). Unlike our observations with MyoD (Fig. 2d), GR binding strength was comparable between the prebound and de novo groups (Fig. 7c). However, GR motifs were only enriched on de novo sites, but not on prebound ones (Fig. 7d). We therefore focused our analysis on the 586 de novo sites to evaluate the interplay between GR and chromatin modifiers.

Among 586 GR^+^ enhancers with de novo binding of KMT2D, cBAF, and p300, acute loss of KMT2D protein or cBAF and p300 enzymatic activities reduced GR occupancy on 219 (37%), 420 (72%), or 260 (44%) of these sites, respectively (Fig. 7e). These results demonstrate the critical roles of KMT2D, cBAF, and p300 in de novo binding of GR on enhancers. On these 586 GR^+^ enhancers, KMT2D depletion reduced cBAF and p300 binding on 334 (57%) and 459 (78%), respectively. Loss of cBAF enzymatic activity also decreased KMT2D, cBAF, and p300 binding on 415 (71%), 448 (76%), and 521 (89%) of these sites, respectively. Meanwhile, inhibition of p300 catalytic activity diminished the binding of KMT2D, cBAF, and p300 on 328 (56%), 448 (76%), and 440 (75%) of these enhancers, respectively (Fig. 7e).

Among 586 GR^+^ enhancers with de novo binding of KMT2D, cBAF, and p300, we identified 109 sites that showed less than a 2-fold reduction in GR binding across all three perturbations. We refer to these regions as cBAF-KMT2D-p300-independent GR^+^ enhancers (Fig. 7f). On these sites, KMT2D depletion mildly reduced BAF binding but markedly diminished p300 enrichment. Inhibition of cBAF enzymatic activity disrupted the binding of all three factors—KMT2D, cBAF, and p300—with p300 being the most impacted. In contrast, p300 inhibition did not affect KMT2D binding and, although significant, caused only modest decreases in cBAF and p300 occupancy. Together, these data highlight the interdependent relationships of chromatin modifiers on GR^+^ enhancers (Fig. 7f).

**Fig. 8:**
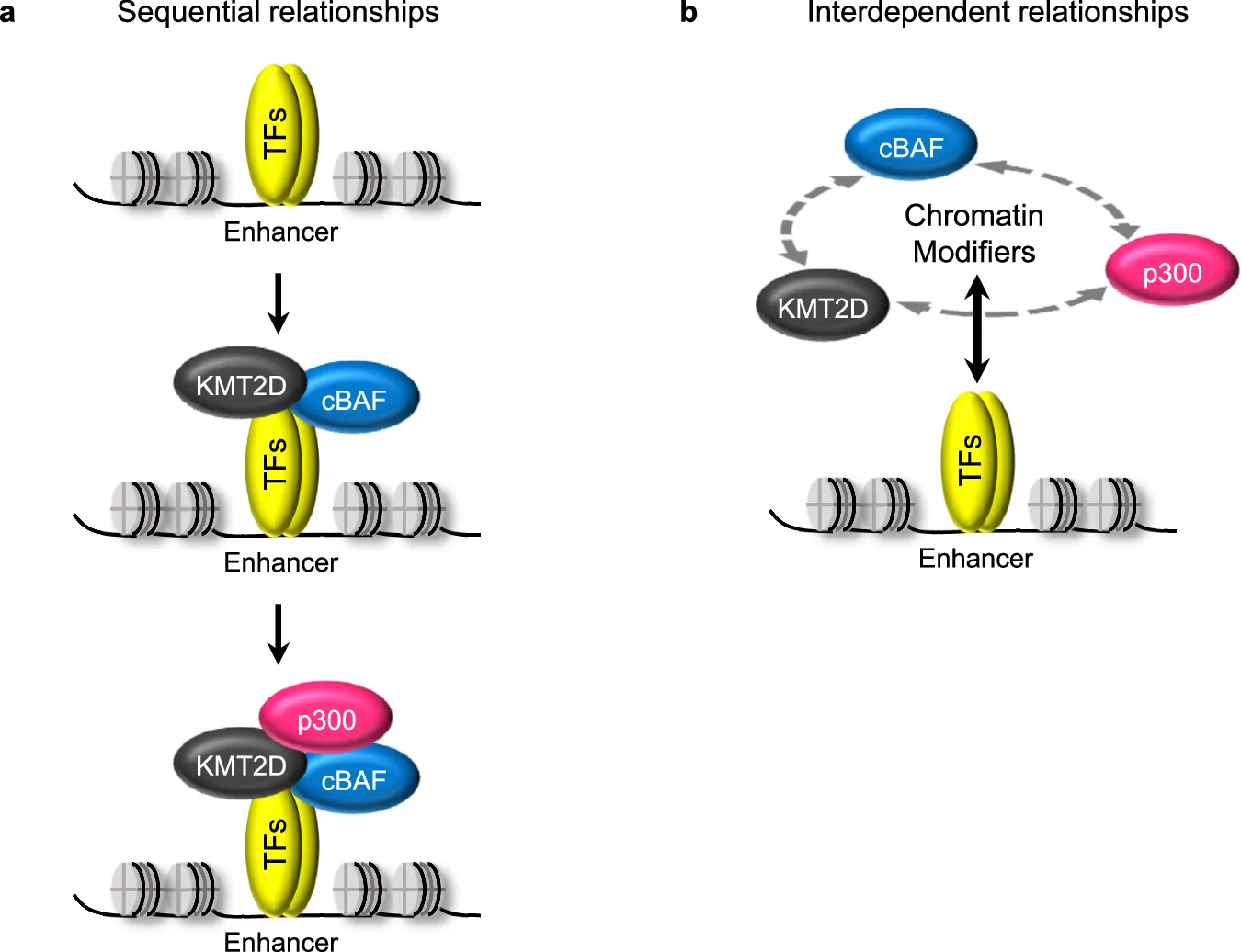
Acute interventions reveal interdependencies among TFs and chromatin modifiers KMT2D, cBAF and p300 on enhancers. **a** The reported sequential relationships of TF and chromatin modifier enrichment on enhancers established with stable knockout and continuous TF expression models. **b** The interdependent relationships between TF and chromatin modifiers KMT2D, cBAF, p300 and among chromatin modifiers on enhancers established with acute interventions of chromatin modifiers and inducible nuclear translocation of TFs.

In conclusion, using the LDTF MyoD and the SDTF GR as models and employing acute perturbation of chromatin modifiers, our approach reveals that TF and chromatin modifier binding on enhancers involves both sequential and interdependent relationships, with the degree of interdependency being particularly prominent at de novo sites (Fig. 8a-b).

## Discussion

In this study, using robust nuclear translocation systems of MyoD and GR combined with acute depletion of KMT2D protein or short-term inhibition of cBAF or p300 enzymatic activity, we illustrated that TFs and chromatin modifiers exhibit interdependent relationships on enhancers. Acute depletion or inhibition of these chromatin modifiers disrupts de novo binding of MyoD and GR as well as that of KMT2D, cBAF, and p300 on enhancers, with loss of cBAF activity having the most pronounced impact. Inhibiting acetyltransferase activity of p300—a chromatin factor frequently positioned hierarchically downstream of TFs, KMT2D, and cBAF—reveals its critical role in facilitating TF binding on enhancers. Our data demonstrate that TFs and chromatin modifiers shape the enhancer landscape not through a strict structural hierarchy, but through a spectrum of hierarchical and cooperative mechanisms.

### MyoD-dependent enhancer assembly

The enrichment and function of MyoD on enhancers have been widely investigated over the years. In C2C12 myoblasts and myotubes, where MyoD is constitutively expressed, MyoD binds to thousands of genomic regions^31^. ChIP-Seq analyses in these systems identified MyoD-associated muscle enhancers marked by H3K4me1, H3K27ac, p300, and RNA Pol II. Further targeted studies in *MyoD* knockout myoblasts showed loss of enhancer features and chromatin modifier binding. Ectopic expression of MyoD in *MyoD* knockout myoblasts partially rescues the enhancer features, suggesting that MyoD plays a direct role in orchestrating the assembly of enhancer machinery^32^. The relationship between MyoD and enhancer-associated factors has been proposed to be interdependent. During myogenesis, AKT-mediated phosphorylation of p300 is required for its interaction with MyoD, as well as for the recruitment of MyoD, p300, and the chromatin remodeling activity of BRG1 on *Myog* and *Ckm* promoters^33^. While these studies establish that MyoD recruits chromatin modifiers to shape the enhancer landscape, the reciprocal question, how chromatin modifiers enable MyoD binding, has remained less systematically addressed. Using acute perturbation of KMT2D, cBAF, and p300, combined with CUT&RUN and ChIP-seq analyses, we systematically assess and quantify the requirements of these chromatin modifiers across MyoD-bound enhancers during myogenic conversion of non-myogenic preadipocytes.

MyoD binding, in cooperation with CTCF binding, has recently been shown to directly rewire the 3D chromatin architecture during human fibroblast-to-myotube conversion^34^. In myoblasts, H3K27ac on MyoD-induced loops is MyoD-dependent. However, p300-mediated H3K27ac alone is not sufficient to drive chromatin looping in the absence of MyoD binding^35^. This finding complements our observation that p300 is required for MyoD binding, suggesting an interdependent relationship between p300 and MyoD. How chromatin modifiers contribute to MyoD-induced chromatin looping requires further investigation, and acute intervention approaches, such as those presented in this study, will provide improved resolution into these relationships.

### MyoD as a dual regulator of gene expression

Our analysis of nascent RNA-seq data supports a dual role for MyoD in transcriptional activation and repression during myogenic reprogramming. MyoD nuclear translocation promoted the upregulation of myogenic genes. In parallel, we observed repression of genes associated with non-myogenic developmental processes, consistent with a report that MyoD can act as a direct transcriptional repressor at non-E-box motifs^36^. Notably, repressed gene loci were enriched with early and transient MyoD binding. MyoD-associated gene repression was accompanied by enhancer decommissioning and deactivation, evidenced by the loss of H3K4me1 and H3K27ac. The mechanism by which MyoD triggers enhancer deactivation likely involves a functional interaction with chromatin modifiers. Although our perturbation analyses primarily focused on cooperative recruitment of KMT2D, cBAF, and p300 at MyoD^**+**^ enhancers, our results do not exclude the possibility that these chromatin modifiers may also contribute, directly or indirectly, to MyoD-associated repression. Defining how KMT2D, cBAF, p300, and other chromatin modifiers participate in MyoD-mediated gene repression will be an important direction for future studies.

### Hierarchical and cooperative mechanisms shaping enhancer establishment

Studies have documented the complexity of TF-chromatin factor relationships, supporting both hierarchical and cooperative aspects^37^. Even pioneer TFs, which can bind closed chromatin, often leverage preexisting chromatin landscapes and exhibit reciprocal interactions with other factors. These aspects have been elegantly illustrated by a study using reconstituted nucleosome arrays. PU.1, a pioneer TF, recruits cBAF chromatin remodeler (a hierarchical mechanism), and cBAF in turn stabilizes weak PU.1-chromatin interactions (a cooperative mechanism)^38^. Thus, TF binding directs recruitment of chromatin remodeler, while remodeler activity reciprocally reinforces TF binding. Consistent with this framework, our data from MyoD and GR models support both hierarchical and cooperative mechanisms underlying TF-chromatin remodeler interactions. Cooperativity is also observed among different TFs. For instance, Foxa2 preferentially binds accessible chromatin pre-marked with active modifications and requires cooperative binding with Gata4 for robust chromatin opening during ESC-to-endoderm differentiation^39^. Similarly, GATA6 binds pre-accessible chromatin to repress pluripotency while binding closed chromatin to activate primitive endoderm differentiation programs. SOX17, activated downstream of GATA6, subsequently reinforces GATA6 binding and enhances chromatin opening, showing both hierarchical and cooperative relationships among TFs^40^. Together, these studies, along with our data, support that TF-chromatin modifier relationships involve both hierarchical and cooperative mechanisms with varying magnitudes of contributions.

### Refining KMT2D’s role in TF and chromatin modifier binding with acute protein depletion

Degron-based protein depletion systems offer a powerful approach to study protein functions in living cells and animals. The key advantage lies in their ability to reveal the primary protein functions before the interference of secondary effects or cellular adaptation. Previously, we found that KMT2D knockout reduces C/EBPβ binding on approximately 26% of enhancers^2,8^. On C/EBPβ-activated enhancers, KMT2D knockout impairs cBAF binding, regardless of whether BAF binding is de novo or pre-marked^8^. In the current study, results from acute KMT2D depletion experiments not only validated prior observations on TF binding but also distinguished KMT2D’s role on pre-existing and newly established chromatin factor binding: prebound cBAF remained stable, while de novo cBAF binding to MyoD^+^ enhancers was significantly reduced upon the loss of KMT2D (Fig. 3e). This refines and specifies KMT2D’s role in facilitating de novo binding of chromatin modifiers alongside modulating de novo binding of TFs.

### cBAF activity in modulating de novo binding of TF and chromatin modifiers on enhancers

BRG1, through regulating chromatin accessibility, is important for maintaining binding of TFs OCT4 and REST in undifferentiated ESCs^24,41^. We show that BRG1 activity is required for de novo binding of MyoD during myogenic reprogramming of preadipocytes, as well as for DEX-induced GR binding (Figs. 4 and 7). Reduced MyoD binding upon BRG1 inhibition extends beyond a simple decrease in chromatin opening. On MyoD^+^ enhancers with de novo cBAF binding, loss of BRG1 activity disrupts de novo MyoD binding on ~65% of the sites, while chromatin accessibility is decreased on only 34% of these sites (Fig. 4c and e). BRG1 inhibition may alter nucleosome positioning without affecting overall chromatin accessibility, supported by a study reporting that BRG1 depletion disrupts nucleosome organization around key TF motifs in T cells^14^. We observed that BRG1 inhibition reduced ARID1A binding, even when MyoD binding persisted (Figs. 4c and 6f). It is plausible that BRG1 activity is required for maintaining the integrity and/or stability of the cBAF complex itself, which in turn reinforces and stabilizes TF binding on these de novo enhancers.

Our observations of reduced ARID1A binding upon BRG1 inhibition align with CUT&RUN analysis showing a moderate decrease in BRG1 occupancy upon BRG1i treatment in ESCs^42^, but contrasts with ChIP-seq data indicating a slight increase^43^. Acute BRG1 protein depletion has been shown to reduce p300 binding in T cells^14^, and KMT2D binding on C/EBPβ-bound enhancers^8^. Consistently, upon BRG1 inhibition, both KMT2D and p300 binding are reduced on MyoD^+^ enhancers (Fig. 4c). Together, these findings suggest that cBAF enzymatic activity facilitates de novo TF binding and the enrichment of cBAF complex and other chromatin modifiers on enhancers, partially through promoting chromatin accessibility.

Although our analysis focuses on ARID1A-associated cBAF throughout this study, mammalian SWI/SNF complexes in muscle are heterogeneous. Previous studies have shown that BRG1- and BRM-containing complexes can have distinct functions during myogenesis, with BRG1 contributing to early muscle gene activation and BRM contributing to cell-cycle arrest and late muscle gene expression^44,45^. In addition, muscle-specific SWI/SNF functions can involve other subunits. Thus, profiling additional components such as BRM, BAF47, and BAF60c would further define the diversity of SWI/SNF assemblies during muscle enhancer regulation.

### p300 activity in facilitating de novo TF and chromatin modifier binding on enhancers

The potent CBP/p300 inhibitor A-485 enables extensive assessment of the role of CBP/p300 acetyltransferase activity in transcriptional regulation. In ESCs, A-485 treatment results in a significant reduction in global transcription within 30 min, without affecting the chromatin binding of pluripotency TFs NANOG or OCT4^46^. This suggests that CBP/p300 acetyltransferase activity is dispensable for maintaining pre-established TF occupancy. In contrast, we found that p300 inhibition disrupts the de novo binding of MyoD on enhancers, an observation also supported by acute p300 degradation in preadipocytes and further confirmed in physiologically relevant C2C12 myoblasts (Figs. 5c, 5f, and Supplementary Fig. 5). Loss of p300 activity also impacts the binding of GR on enhancers (Fig. 7). Our results align more with imaging studies at the *Pou5f1* enhancer, where Sox2 recruits CBP/p300, and CBP/p300 acetyltransferase activity reinforces Sox2 occupancy^47^. In addition, while p300 inhibition does not impact steady-state p300 chromatin binding in ESCs or leukemic MM1.S cells^46,48^, we show that p300 activity is required for de novo p300 binding on newly established MyoD^+^ or GR^+^ enhancers.

Despite a reduced enrichment of cBAF on enhancers upon p300 inhibition, we found that chromatin accessibility remains intact (Fig. 5d). This is consistent with other reports showing that CBP/p300 inhibitor does not affect chromatin opening^46,48^. This also indicates that impaired TF binding upon the loss of CBP/p300 enzymatic activity is not due to loss of accessible chromatin. While CBP/p300 are known to acetylate many TFs and enhancer-associated chromatin factors^49^ including multiple lysine residues on MyoD itself^29,30^, our analysis of the non-acetylatable MyoD-K/R mutant reveals that loss of MyoD acetylation does not impair its own chromatin binding. Thus, disrupted MyoD binding observed upon p300 inhibition is not driven by MyoD deacetylation. It is possible that acetylation on histones or other chromatin modifiers is necessary for stabilizing MyoD binding on enhancers. Nevertheless, we demonstrate that MyoD acetylation is required for the optimal recruitment and/or stabilization of KMT2D, cBAF, and p300 on these MyoD-bound enhancers.

In summary, using robust nuclear translocation models of MyoD and GR, combined with acute perturbations of chromatin modifiers, our study demonstrates that KMT2D, cBAF, and p300 are active, interdependent facilitators of de novo TF binding on enhancers. These chromatin modifiers also modulate each other’s binding on TF-bound enhancers. Future studies using higher temporal resolution techniques, such as live-cell imaging or optogenetics, may help define the precise order, kinetics, and relationships of chromatin modifier recruitment during the initiation of enhancer activation.

## Methods

### DNA constructs/plasmids

MyoD-ER(T) cDNA was PCR amplified from pLv-CMV-MyoD-ER(T) plasmid (#26809, Addgene)^20^ and subcloned into pCW57.1puro-MCS-3xT7^50^ to generate MyoD-ER(T)−3xT7. MyoD-ER(T)−3xT7 cDNA was further subcloned into pWZLhygro (#18750, Addgene) to generate pWZLhygro-MyoD-ER(T)−3xT7. pWZLhygro-MyoD-K/R-ER(T)−3xT7 where lysines K99, K102, and K104 of MyoD were mutated to arginines (R) was generated from pWZLhygro-MyoD-ER(T)−3xT7 with Q5^®^ Site-Directed Mutagenesis Kit (E0554S, NEB). pBabepuro-OsTIR1(F74G)−9xMyc was generated from pBabepuro-OsTIR1-9xMyc (#80074, Addgene) by site-directed mutagenesis. All plasmids were confirmed by Sanger DNA sequencing.

### Antibodies and chemicals

Anti-T7 (D9E1X, #13246; 1:1000 for WB, 1 µL per CUT&RUN)), anti-ARID1A/BAF250A (D2A8U, #12354; 1:1000 for WB, 1 µL per CUT&RUN), and anti-p300 (D8Z4E, #86377; 1:1000 for WB, 1 µL per CUT&RUN) were from Cell Signaling Technology. Rabbit control IgG (#13-0042 K) and anti-H3K4me1 (#13-0040; 1:2000 for WB, 0.5 µg per CUT&RUN) were from EpiCypher. Anti-H3K27ac (ab4729; 1:1000 for WB, 0.5 µg per CUT&RUN), anti-BRG1 (ab110641; 1:5000 for WB), and anti-H3 (ab1791; 1:8000 for WB) were from Abcam. Anti-p300 (C-20, sc-585; 1:1000 for WB), anti-MyoD (5.8 A, sc-32758; 1:1000 for WB, 4 µg per ChIP), anti-GR (M-20, sc-1004; 1:1000 for WB, 4 µg per ChIP), and anti-Myc (9E10, sc-40; 1:5000 for WB) were from Santa Cruz. Anti-RbBP5 (A300-109A; 1:5000 for WB) was from Bethyl Laboratories. Anti-mAID Tag (NBP2-89163; 1:1000 for WB) was from Novus Biologicals. The homemade anti-KMT2D, anti-KMT2C and anti-UTX have been previously described^51^. Secondary antibodies used were anti-rabbit IgG, HRP-linked (#7074S; 1:2000 for WB) and anti-mouse IgG, HRP-linked (#7076S; 1:2000 for WB) from Cell Signaling Technology.

BRG1 Inhibitor BRM014 (#HY-119374) and 5Ph-IAA (#HY-134653) were from MedChemExpress (MCE) and used at 1 µM. PROTAC p300/CBP degrader dCBP-1 (#HY-134582) from MCE was used at 250 nM. p300/CBP inhibitor A-485 (#6887) was from Tocris Bioscience and used at 3 µM. (Z)−4-Hydroxytamoxifen (4-OHT) (#H7904) and Dexamethasone (DEX) (#D4902) were from Millipore-Sigma and used at 400 nM and 100 nM, respectively.

### Mouse strains and housing

All animal experiments were conducted in compliance with relevant ethical regulations and were approved by the Animal Care and Use Committee of the National Institute of Diabetes and Digestive and Kidney Diseases (NIDDK), National Institutes of Health (NIH). Mice were housed in a room with a controlled temperature of 22 ^o^C and humidity of 45–65% under a 12 h light and 12 h dark cycle.

The newly generated *Kmt2d* ^AID/AID^ mice, on a C57BL/6J background, were created by inserting a 132 bp DNA fragment encoding AID tag (sequence details provided in Supplementary Fig. 4a) after the start codon located on exon 2 of *Kmt2d* by CRISPR/Cas9-mediated homology-directed repair using the gRNA: 5’-TGTCCATCCCTCTCTCCGAC-3’. Successful integration of AID tag was confirmed with genotyping and Sanger DNA sequencing. This mouse line has been deposited at The Jackson Laboratory (JAX Stock #041109). *Kmt2d* ^AID/AID^ mice were crossed with *Kmt2c* ^f/f^; *Cre-ER* mice^22^ to obtain *Kmt2c* ^f/f^; *Kmt2d* ^AID/AID^; *Cre-ER* mice. Sex was not considered as a biological variable for the in vitro experiments.

### Preadipocyte cultures and myogenesis assay

The *Kmt2c*^−/−^; *Kmt2d* ^AID/AID^ preadipocyte cell line was generated for this study as described in the Supplementary Fig. 4. Briefly, primary brown preadipocytes were isolated from interscapular brown adipose tissue of male and female newborn pups and immortalized by infection with retroviruses carrying pBABE-neo large T cDNA expressing SV40T^52^. Immortalized preadipocytes were sequentially infected with retroviruses expressing pBabepuro-OsTir1(F74G)−9xMyc and pWZLhygro-MyoD-ER(T)−3xT7. The immortalized preadipocytes were routinely cultured in growth media: DMEM (#10-013-CV, Corning) supplemented with 10% Foundation FBS (#SH30910, Cytiva). C2C12 myoblasts (ATCC, CRL-1772) were cultured in DMEM supplemented with 20% Foundation FBS.

For myogenesis assay, preadipocytes and C2C12 were grown in growth media for 2 days. On the day of experiments, the growth medium was replaced with differentiation medium: DMEM supplemented with 2% horse serum (#H1270, Millipore-Sigma). For the differentiation assay that lasts more than 48 h, the media was changed after 48 h and every two days thereafter.

To induce MyoD-ER-T7 and GR translocation, 4-OHT and DEX were used at the final concentration of 400 nM and 100 nM, respectively. BRM014 and 5Ph-IAA were used at the final concentration of 1 µM to inhibit BRG1 chromatin remodeling activity and deplete KMT2D, respectively, while A-485 was used at 3 µM to inhibit CBP/p300 acetyltransferase activity.

### Western blot

Nuclear proteins were extracted from preadipocytes with a high-salt method. Briefly, cells were washed with cold PBS, resuspended in buffer A (10 mM HEPES, pH 7.9, 1.5 mM MgCl_2_, 10 mM KCl, and 0.1% NP-40) supplemented with protease inhibitors (PIs, #04693159001, Roche), and incubated on ice for 10 min. Nuclei were harvested by 10-min centrifugation at 2000 × *g*. Nuclear proteins were extracted in buffer C (20 mM HEPES pH 7.9, 1.5 mM MgCl_2_, 420 mM NaCl, 0.2 mM EDTA, and 25% glycerol, supplemented with PIs) for 20 min on ice. Nuclear extracts were separated using 4–15% Tris-Glycine Gradient Gels (#4561085, Bio-Rad) or 3–8% NuPAGE™ Tris-Acetate Mini Protein Gel (#EA0375BOX, ThermoFisher). Proteins were transferred to PVDF membranes, using the iBlot 2 Gel Transfer Device (Invitrogen) or wet transfer method. The membranes were probed with specific antibodies.

Histones were extracted with the following protocol. Nuclei were first isolated from preadipocytes with Triton Extraction Buffer (PBS containing 0.5% Triton X-100 and PIs), on ice for 10 min. Histones were extracted with 0.2 N HCl on ice for >2 h. The histone-containing supernatants were then neutralized with 10% 2 M NaOH (v/v). Histones were separated using 4–15% Tris-Glycine Gradient Gels and transferred to PVDF membranes using the Trans-Blot Turbo Transfer System (Bio-Rad). The membranes were probed with specific antibodies.

### RNA isolation and qRT-PCR analysis

Total RNA was extracted using TRIzol (#15596018, Invitrogen) and reverse transcribed using ProtoScript II first-strand cDNA synthesis kit (#E6560, NEB), following manufacturer’s instructions. Quantitative RT-PCR (qRT-PCR) was performed in triplicate with the Luna® Universal qPCR Master Mix (#M3003, NEB) using QuantStudio 5 Real-Time PCR System (Applied Biosystems™). PCR amplification parameters were 95 °C (3 min) and 40 cycles of 95 °C (15 s), 60 °C (60 s), and 72 °C (30 s). Statistical significance was calculated using the two-tailed unpaired *t*-test on two experimental conditions. qRT-PCR of *Myog* and *Ckm* was done using the following primers: *Myog*: forward 5’-CCCTGCCCCACAGGGGCTGTG-3’ and reverse 5’-ACGCCACAGAAACCTGAGCCC-3’; *Ckm*: forward 5’-CGCCAGCTAGACTCAGCACT-3’ and reverse 5’-CCCTGCGAGCAGATGAGCTT-3’.

### CUT&RUN

CUT&RUN was performed using CUTANA™ ChIC/CUT&RUN Kit (#14-1048, Epicypher, version 4), following the manufacturer’s instructions with modifications. 0.5 × 10^6^ preadipocytes were used for each CUT&RUN reaction. Cells were gently fixed with 0.1% formaldehyde for 1 min at room temperature (RT). After two PBS washes, cells were scraped out in PBS and harvested via centrifugation at 600 *g* for 3 min. Cells were resuspended in 100 µL cold Nuclei Extract Buffer (20 mM HEPES pH 7.9, 10 mM KCl, 0.1% Triton X-100, 20% Glycerol, 1 mM MgCl_2_ supplemented with PIs and 0.5 mM Spermidine) and lysed on ice for 10 min. Nuclei were then harvested via centrifugation, resuspended in cold Nuclei Extract Buffer, and incubated with activated 10 µL Concanavalin A beads for 10 min at RT. The cell-beads were captured by a magnetic stand and were incubated with the desired primary antibody in 50 µL of cold Antibody buffer (wash buffer plus 0.01% Digitonin and 2 mM EDTA) overnight at 4^o^C. Any unbound antibody was washed twice with 200 µL cold Cell Permeabilization buffer (wash buffer plus 0.01% Digitonin). The supernatant was discarded via bead liquid separation using a magnetic stand. 2.5 µL pAG-MNase was added, and the complex was incubated for 10 min at RT. The bound complexes were then washed twice with 200 µL cold Cell Permeabilization buffer. To activate pAG-MNase, 100 mM CaCl_2_ was added to the bound complexes. The digestion reaction was carried out at 4 °C for 2 h and neutralized by the addition of STOP Buffer Master Mix including 1 ng *E. coli* Spike-in DNA, followed by an incubation at 37 °C for 10 min to release cleaved DNA fragments. The released DNA was separated out using a magnetic stand. The supernatant containing CUT&RUN-enriched DNA was purified via a spin column.

CUT&RUN enriched DNA was used for library construction. Library construction was performed using the NEBNext Ultra™ II DNA Library Prep Kit for Illumina (#E7645L, NEB) and NEBNext Multiplex Oligos for Illumina (#E6440L, NEB). All reaction volumes were reduced to half of the recommended reaction volume. Adapter ligation was performed using the NEB Adapter stock diluted by 10-fold. Post adapter ligation, 1 µL NEB USER enzyme was added to the reaction and incubated at 37 °C for 15 min. After adapter ligation and hairpin cleavage, 1X AMPure XP Beads (#A63881, Beckman Coulter) were added to the reaction to recover DNA fragments. Purified DNA was amplified with the NEBNext Multiplex Oligos. To avoid amplification of longer DNA fragments, the following PCR cycling parameters were used: 98 °C (45 s); 14 cycles at 98 °C (15 s) and 60 °C (10 s); and 98 °C (60 s). The amplified libraries were purified with 1X AMPure XP Beads. The libraries were paired-end sequenced on the Illumina NovaSeqX or NextSeq2000 System.

### Nascent RNA-seq library construction

Cells were incubated with 0.5 mM 5-ethynyl uridine (EU) for 30 min prior to harvest. Total RNA was isolated, and 1 µg was used for ribosomal RNA depletion using the NEBNext rRNA Depletion Kit (NEB, E7405L). Newly synthesized RNA was then selectively labeled and enriched via biotin conjugation using the Click-iT Nascent RNA Capture Kit (Thermo Fisher, C10365) following the manufacturer’s guidelines. The enriched RNA fraction was reverse transcribed into cDNA using the SuperScript VILO cDNA Synthesis Kit (Thermo Fisher, 11754050). cDNA libraries were subsequently prepared with the NEBNext Ultra II RNA Library Prep Kit for Illumina (NEB, E7770) according to standard protocols. Final libraries were quantified using Qubit, pooled, and sequenced on a NovaSeq X platform.

### ChIP-seq

Chromatin immunoprecipitation (ChIP) was performed as previously described^8^. In summary, cells were cross-linked with 1–2% formaldehyde for 10 min and then quenched using 125 mM glycine at RT. The cells were washed with cold PBS twice, and nuclei were harvested in Farnham lysis buffer (5 mM PIPES (pH 7.5), 85 mM KCl, 1% NP-40, supplemented with PIs). Nuclei were then resuspended in TE (10 mM Tris-Cl pH7.6 and 1 mM EDTA) and sonicated for 17 min (30 s on/off cycle). Lysates were supplemented with detergents to make 1X RIPA buffer (10 mM Tris-Cl pH 7.6, 1 mM EDTA, 0.1% SDS, 0.1% sodium deoxycholate, 1% Triton X-100, PIs) and centrifuged at 13,000 *g* for 10 min at 4 °C to remove debris. The supernatant containing sheared DNA was transferred to a new tube for ChIP. Each ChIP reaction included 4–10 µg of target antibodies, 20 ng of spike-in chromatin (#53083, Active Motif), and 2 µg of spike-in antibody (anti-H2Av, #61686, Active Motif), incubated on a rotator at 4 °C overnight. ChIP samples were then mixed with 50 μL of prewashed Protein A Dynabeads (#10002D, ThermoFisher) and incubated for 3 h at 4 °C. The beads were collected using a magnetic rack and washed twice with RIPA buffer, RIPA buffer containing 300 mM NaCl, cold LiCl buffer, and PBS. ChIP and input samples were added to 100 μL of ChIP elution buffer containing 0.1 M NaHCO3, 1% SDS, and 20 μg of Proteinase K, and incubated at 65 °C overnight. ChIP DNA was purified using the QIAquick PCR purification kit (#28104, Qiagen). Entire ChIP DNA or 300 ng of the input DNA were used to construct libraries with NEBNext Ultra™ II DNA Library Prep Kit for Illumina, NEBNext Multiplex Oligos for Illumina, and AMPure XP Beads. The libraries were paired-end sequenced on the Illumina NovaSeqX System.

### ATAC-seq

The Assay for Transposase-Accessible Chromatin with high-throughput sequencing (ATAC-seq) was performed using ATAC-seq Kit (#53150, Active Motif), following the manufacturer’s protocols. In short, 100,000 cells were freshly collected for each ATAC reaction. After one PBS wash, cells were resuspended in 100 µL ice-cold ATAC lysis buffer and immediately spun down at 500 *g* for 10 min at 4 °C. Cell pellet was then incubated in 50 µL Tagmentation Mix at 37 °C for 30 min in a thermomixer set at 800 rpm. Tagmented DNA was then purified via a spin column and amplified using i7- and i5-index primers provided in the kit. The amplified library was purified with 1.2X SPRI bead solution, following the kit instruction. Samples were paired-end sequenced on the Illumina NextSeq2000 System.

### Computational analysis

#### CUT&RUN, ChIP-seq, ATAC-seq data processing

Raw sequencing data were aligned to the mouse mm10 genome (CUT&RUN, ChIP-seq, and ATAC-seq), the *E. coli* genome eck12 (CUT&RUN), and *Drosophila* genome dm6 (ChIP-seq) using bowtie2 (v.2.3.4.1).

All datasets were normalized using spike-in read counts prior to downstream analyses. For CUT&RUN, normalization factors were determined in two steps. First, the ratio of *E. coli* eck12 reads to mouse mm10 reads was calculated for each sample. Second, the normalization factors were obtained by dividing the lowest ratio of eck12/mm10 by the ratio of each corresponding sample. For ChIP-seq data, normalization factors were determined by dividing the lowest *Drosophila* dm6 reads by the dm6 reads of each corresponding sample. Normalized read counts for all datasets were adjusted by multiplying the read counts by their respective normalization factors.

#### CUT&RUN and ChIP-seq peak calling

Following alignment, peaks were identified using the SICER2 algorithm [https://zanglab.github.io/SICER2/]^53^. For peak calling of H3K4me1 and H3K27ac, we used the window size of 200 bp, the gap size of 200 bp, and the false discovery rate (FDR) of 1E-3. For peak calling of non-histone factors, we used the window size of 50 bp, the gap size of 50 bp, and the FDR of 1E-10. An effective genome fraction of 0.8 was applied to all datasets. All other parameters were set to default values.

#### Motif analysis

To identify TF binding motifs enriched within the genomic regions of interest, we used the HOMER motif discovery algorithm (v4.7.2) [http://homer.ucsd.edu/homer/]^54^, executing the findMotifsGenome.pl command with default parameters. For both the known and de novo results output, we selected and presented representative TFs from each family that share the same consensus sequence to avoid redundancy. For graphical representation, -log_10_(*p*-values) were visualized using bubble plots. Values were capped at 100 for dot size scaling to improve readability while preserving relative enrichment patterns.

#### Clustering of MyoD-ER binding regions

To investigate the dynamics of MyoD-ER genomic binding across time points after 4-OHT treatment, read counts were calculated on the union of MyoD-ER sites (Fig. 1). Normalization was applied using two methods: (1) scaling by total reads on peaks and (2) applying spike-in normalization factors derived from the ratio of *E. coli* Eck12 reads to mouse mm10 reads. Z-scores were calculated for each genomic region by subtracting the mean read count across the time points and dividing by the standard deviation. These Z-scores were used for k-means clustering and visualized as heatmaps using the gplots package (v3.2.0) in R.

#### GREAT GO analysis

To associate biological functions with genomic regions of interest, we used the Genomic Regions Enrichment of Annotations Tool (GREAT) (https://great.stanford.edu/great/public/html/index.php)^55^. GREAT uses proximity as well as curated regulatory domains to associate non-coding genomic regions to nearby genes, and tests for enrichment of functional annotations such as Gene Ontology (GO) terms.

#### Nascent RNA-seq data processing and differential expression analysis

Raw sequencing reads were aligned to the mouse mm10 genome using STAR aligner (v2.7.11b)^56^. Reads mapped to ribosomal RNA loci were removed before calculating read counts across gene bodies (transcription start site to transcription end site). Differential expression analysis across the time-course (0 h, 1 h, 4 h, 9 h, and 24 h following MyoD induction) was done with biological duplicates using DESeq2 package (v1.44.0)^57^ in R. To identify transcripts with significant dynamic expression at any time point during 24 h period, a Likelihood Ratio Test (LRT) was used comparing a full model (~Time) to a reduced model (~1). Differentially expressed genes were defined using a threshold of adjusted *p* value < 0.01 and an absolute log_2_ fold change > 0.58 (~1.5-fold).

#### Clustering of differentially expressed genes and DAVID GO analysis

To identify temporal expression patterns of differentially expressed genes, we performed soft clustering using Mfuzz (v2.64.0)^58^ in R. Variance-stabilized transformed counts were extracted from duplicates, averaged, and standardized to z-scores. The fuzzifier parameter (m) was estimated empirically, and gene clustering was done with *c* = 5 clusters. To ensure high confidence of gene assignment, only core genes with membership probability of >0.60 were retained for downstream analysis. GO analysis of five clusters was done using DAVID tool (https://davidbioinformatics.nih.gov/)^59^. The resulting enriched GO terms were manually curated to remove overlapping terms.

#### Clustering of temporal histone marks on MyoD binding regions

H3K4me1 and H3K27ac ChIP-seq read counts were calculated on MyoD binding regions associated with the differentially expressed nascent RNA-seq gene clusters (G1-G5). Normalized RPKM values were transformed to z-scores for comparative analysis. Temporal clustering of each histone mark was performed independently using the Fuzzy c-means (FCM) algorithm implemented in the scikit-fuzzy Python package. Clustering parameters were set to 4 clusters (*c* = 4) with a fuzzifier value of *m* = 2.0. Each genomic region was assigned to the histone cluster for which it showed the highest membership score. Temporal chromatin profiles were visualized as z-score-normalized heatmaps using the seaborn Python library.

#### Integrated MyoD binding, enhancer histone marks, and transcription analysis

To define links between regulatory elements and transcription during MyoD-induced differentiation, MyoD binding clusters (A-E) and histone clusters (H3K4me1 and H3K27ac clusters 1–4) were integrated with the nascent RNA-seq gene clusters (G1–G5). Genomic coordinates of MyoD binding sites and histone-marked regions were annotated using ChIPseeker^60^ with the TxDb.Mmusculus.UCSC.mm10.knownGene database. Peaks were assigned to the nearest transcription start site within 100 kb and linked to genes identified in the Mfuzz-defined nascent RNA-seq clusters. Statistical enrichment between regulatory clusters (MyoD, H3K4me1, and H3K27ac) and transcription clusters was assessed using pairwise Fisher’s exact tests. For MyoD, *p*-values were adjusted using the Benjamini-Hochberg method, and enrichments were visualized as bubble plots using ggplot2^61^ in R. For integrations of histone marks, enrichment strength was quantified as the log_2_ odds ratio and visualized using matplotlib and seaborn in Python. In these plots, bubble size reflects the number of overlapping genes, and color intensity represents the log_2_ odds ratio, scaled between −2 (depletion) and +2 (enrichment) to facilitate direct visual comparison across different histone marks.

#### Heat maps, box plots, violin plots and average profiles

Heat maps were generated using custom Python scripts that processed matrix files with 50 bp resolution and visualized with Seaborn, with customizable color palettes for data representation. To enable comparative analysis across experimental groups, enhancer regions were ordered by signal intensity of MyoD-ER at the center of a 400 bp window under control conditions (4OHT) and log-transformed using the log_2_(x + 1) scale. Box plots were used to display the normalized MyoD-ER or GR read counts (Figs. 2, 6, and 7). Box plots were also used to illustrate the ratios of normalized ARID1A (cBAF) and p300 read counts between KMT2D-depleted and control cells (Fig. 3) or the ratios of normalized T7 (MyoD), KMT2D, ARID1A (cBAF), and p300 read counts between KMT2D-deleted, BRG1 or p300 inhibition and control cells (Fig. 6 and 7). Each box plot shows the first and third quartiles, with a horizontal line indicating the median and whiskers representing the minimum and maximum values. Violin plots were used to visualize the ratios of normalized read counts between p300i- or p300-degrader-treated and control cells, also showing the first and third quartiles and the median (Fig. 5). Outliers were not included. Statistical significance was assessed using a one-sided Wilcoxon signed-rank test or a two-sided, unpaired Mann Whitney test, as indicated in each figure legend. Average profiles were generated using normalized read counts binned at 5 bp intervals, spanning from the center of MyoD-ER binding sites to 2.5 kb upstream and downstream (Figs. 4 and 5).

#### Chromatin accessibility on MyoD^+^ enhancers

Chromatin accessibility on MyoD^+^ enhancers was quantified by calculating ATAC-seq read counts before and after MyoD nuclear translocation (Fig. 4). Three groups of enhancers were defined based on accessibility changes and normalized read counts (RPKM) in MyoD-translocated condition. Constitutively open enhancers were defined as regions showing less than a 2-fold increase in ATAC-seq signal after MyoD translocation and exhibiting accessibility of RPKM > 1. MyoD-dependent opening enhancers were identified as regions showing more than a 2-fold increase in ATAC-seq signal upon MyoD translocation, also with RPKM > 1. Regions with low overall accessibility, defined by RPKM < 1, were designated as low-opening enhancers.

### Statistics and reproducibility

No statistical method was used to predetermine sample size. No data were excluded from the analyses. The experiments were not randomized, as this was an in vitro study utilizing defined cell lines and tightly controlled pharmacological/genetic interventions. The investigators were not blinded to allocation during experiments and outcome assessment, as the distinct genotypes and treatments were known prior to analysis. CUT&RUN and ChIP-seq were independently performed at least twice, and nascent RNA-seq was performed using two independent biological replicates in one experiment, whereas ATAC-seq was performed once. Representative western blots were repeated independently at least twice with similar results. Mann-Whitney tests and Wilcoxon signed-rank tests were performed to compute statistical significance in R, Python, and GraphPad Prism 10 (v10.4.2).

### Reporting summary

Further information on research design is available in the Nature Portfolio Reporting Summary linked to this article.

## Supplementary information

Supplementary Information

Reporting Summary

Transparent Peer Review file

## Source data

Source Data

## Data Availability

The ChIP-seq, CUT&RUN, nascent RNA-seq, and ATAC-seq data generated in this study have been deposited in the NCBI Gene Expression Omnibus (GEO) under accession number GSE290849 and are available under open public access. Source data are provided with this paper.
