# Supplementary material for: Chromatin modifiers KMT2D, cBAF, and p300 cooperatively facilitate de novo binding of transcription factors on enhancers
Source: Nat Commun. 2026 Sep 15;17:10816. doi: 10.1038/s41467-026-77757-w (PMC13669397; doi:10.1038/s41467-026-77757-w)
Supplement: Supplementary file 1 [file 41467_2026_77757_MOESM1_ESM.pdf]

Supplementary Information

Chromatin modifiers KMT2D, cBAF, and p300 cooperatively facilitate *de novo* binding of transcription factors on enhancers

Hieu T. Van[1], Young-Kwon Park[1], Danyang Wan[1], Chengyu Liu[2], Shamima Islam[3], Stefania Dell'Orso[3], Weiqun Peng[4], Vittorio Sartorelli[5], Ji-Eun Lee[1,*], and Kai Ge[1,*]

[1] National Institute of Diabetes and Digestive and Kidney Diseases, National Institutes of Health (NIH), Bethesda, MD 20892

[2] Transgenic Core, National Heart, Lung, and Blood Institute, NIH, Bethesda, MD 20892

[3] Genome Technology Unit, National Institute of Arthritis and Musculoskeletal and Skin Diseases (NIAMS), NIH, Bethesda, MD 20892

[4] Department of Physics and Department of Anatomy and Cell Biology, The George Washington University, Washington, DC 20052

[5] Laboratory of Muscle Stem Cells and Gene Regulation, NIAMS, NIH, Bethesda, MD 20892

*To whom correspondence should be addressed. (Emails: leejieun@niddk.nih.gov, KaiGeLab@outlook.com)

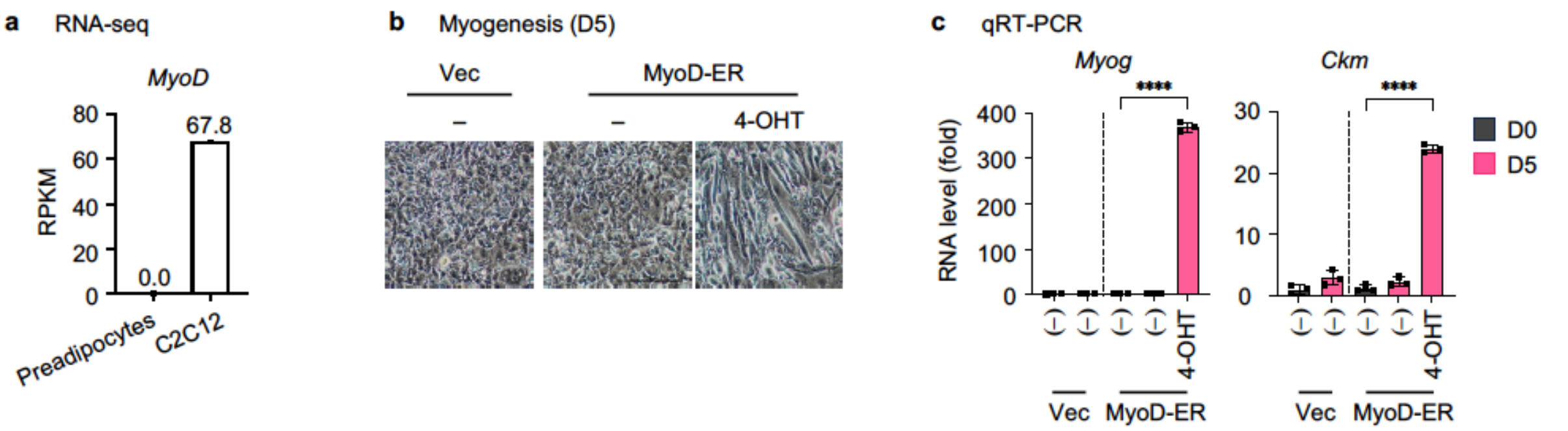

d GO analysis

	GO terms	p-value
A	Cell-substrate adhesion<br>Actin-cytoskeleton organization<br>Regulation of hemopoiesis	1.7E-20<br>1.6E-18<br>1.3E-17
B	Regulation of leukocyte differentiation<br>Cell-substrate adhesion<br>Myeloid cell differentiation	3.0E-31<br>2.7E-27<br>2.9E-23
C	Negative regulation of cytokine production<br>Regulation of cysteine-type endopeptidase activity<br>Regulation of angiogenesis<br>Cell-substrate adhesion	7.8E-09<br>1.1E-08<br>3.5E-08<br>1.1E-07
D	Actin cytoskeleton organization<br>Post-transcriptional regulation of gene expression<br>Regulation of cellular amide metabolic process<br>Regulation of small GTPase mediated signal transduction<br>Striated muscle cell differentiation<br>Muscle cell development	4.0E-39<br>3.8E-25<br>3.0E-24<br>5.6E-24<br>4.5E-18<br>1.0E-16
E	Response to insulin<br>Mitochondrial transport<br>Muscle cell development<br>Regulation of intrinsic apoptotic signaling pathway<br>Striated muscle cell development	1.5E-43<br>3.6E-41<br>5.9E-41<br>2.3E-40<br>5.2E-39
F	Regulation of intrinsic apoptotic signaling pathway<br>Mitochondrial transport<br>Regulation of mitochondrial membrane permeability<br>Positive regulation of transcription	3.2E-34<br>7.2E-34<br>6.4E-29<br>7.2E-25

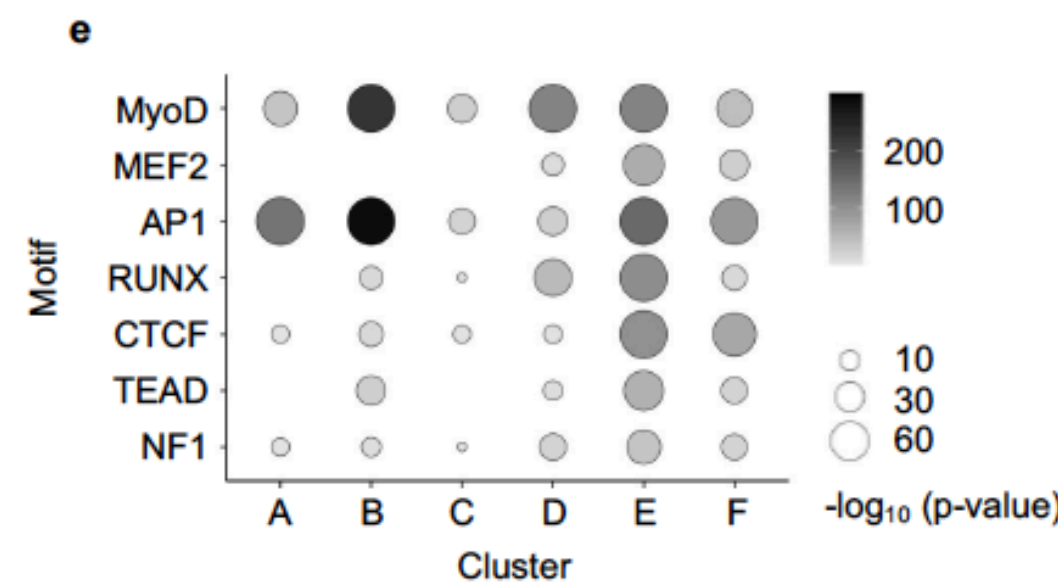

Supplementary Fig. 1. Nuclear-translocated MyoD mediates myogenesis in preadipocytes.
(a) *MyoD* expression in preadipocytes and C2C12 myoblasts was determined using RNA-Seq (n = 1). RPKM values indicate gene expression levels. **(b)** Cell morphology under microscope at day 5 (D5) of myogenesis. **(c)** qRT-PCR analysis of myogenic markers *Myog* and *Ckm* at D0 and D5 of myogenesis (n = 3 biological replicates). Data represent mean ± SD. Statistical significance was determined by ordinary one-way ANOVA followed by Dunnett's multiple comparisons test comparing to the D0 MyoD-ER (-) sample (**** adjusted p-value ≤ 0.0001). **(d)** GREAT GO analysis of genes associated with MyoD binding sites in each cluster defined in Fig. 1e. **(e)** HOMER motif analysis of MyoD binding sites in clusters A-F defined in Fig. 1e. Dot size and color intensity represent $-\log_{10}$(p-value). The maximum dot size was capped at 100 to improve visualization while preserving statistical interpretation. Source data are provided as a Source Data file.

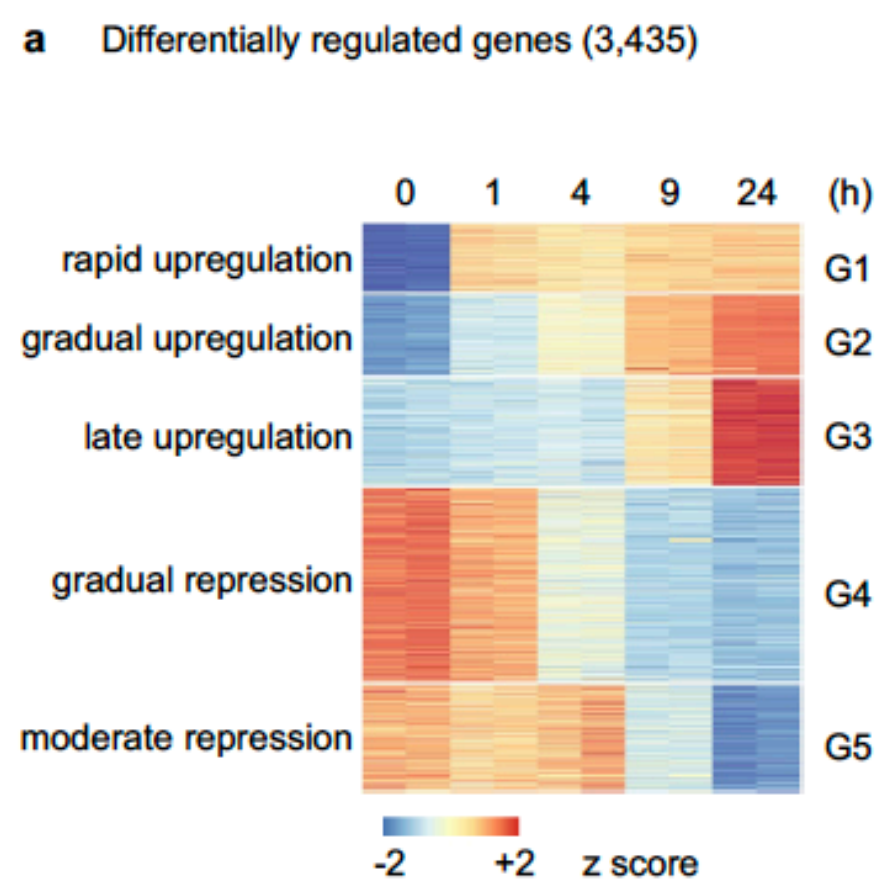

b GO analysis

Group	GO term	Count	P value
G1	cell adhesion	12	7E-03
	Wnt signaling pathway	7	8E-03
	actin cytoskeleton organization	7	9E-03
G2	nervous system development	14	3E-04
	DNA damage response	14	1E-03
	actin cytoskeleton organization	9	1E-03
G3	muscle contraction	11	1E-12
	monoatomic ion transport	20	6E-08
	sarcomere organization	9	7E-08
	muscle fiber development	7	2E-06
G4	cell adhesion	37	1E-07
	angiogenesis	23	4E-07
	ureteric bud development	10	6E-07
	axon guidance	18	8E-07
	osteoblast differentiation	13	7E-06
G5	cellular response to interferon	27	2E-32
	innate immune response	51	3E-27
	defense response to virus	28	3E-18
	immune system process	40	2E-12

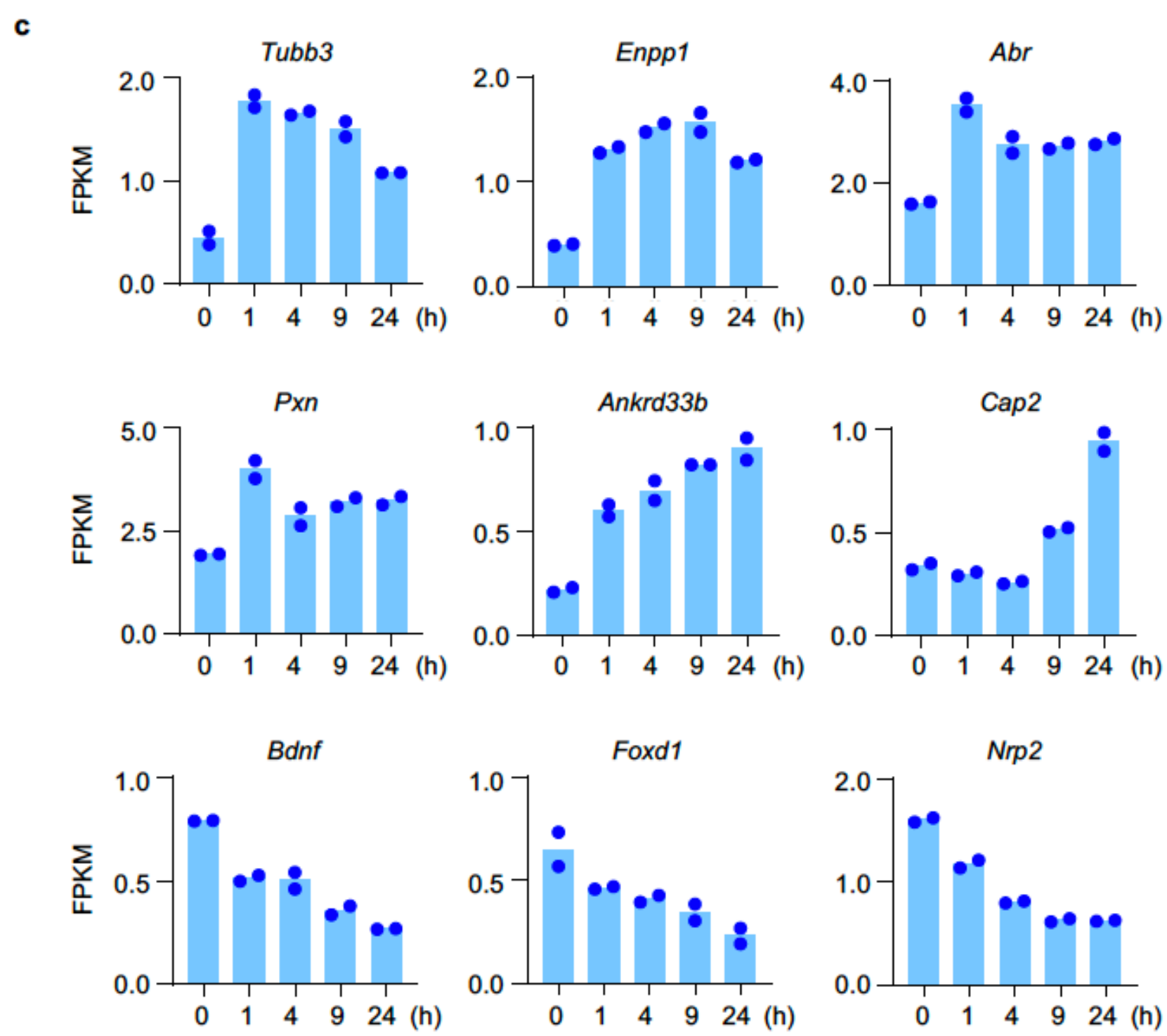

Supplementary Fig. 2. Nascent RNA synthesis upon translocation of MyoD during myogenesis. MyoD-ER-T7 expressing preadipocytes were treated with 4-OHT for 0, 1, 4, 9, and 24 hours, followed by nascent RNA-seq analysis.

(a) Heat maps of soft clustering depicting temporal expression dynamics of differentially expressed genes during MyoD-mediated myogenesis. **(b)** DAVID GO analysis of differentially regulated genes in each cluster defined in (a). **(c)** Nascent RNA expression profiles of representative genes associated with MyoD binding sites peaked at 1h of 4-OHT treatment (n = 2). FPKM values indicate gene expression levels. Please also see the associated main Fig. 1f-g.

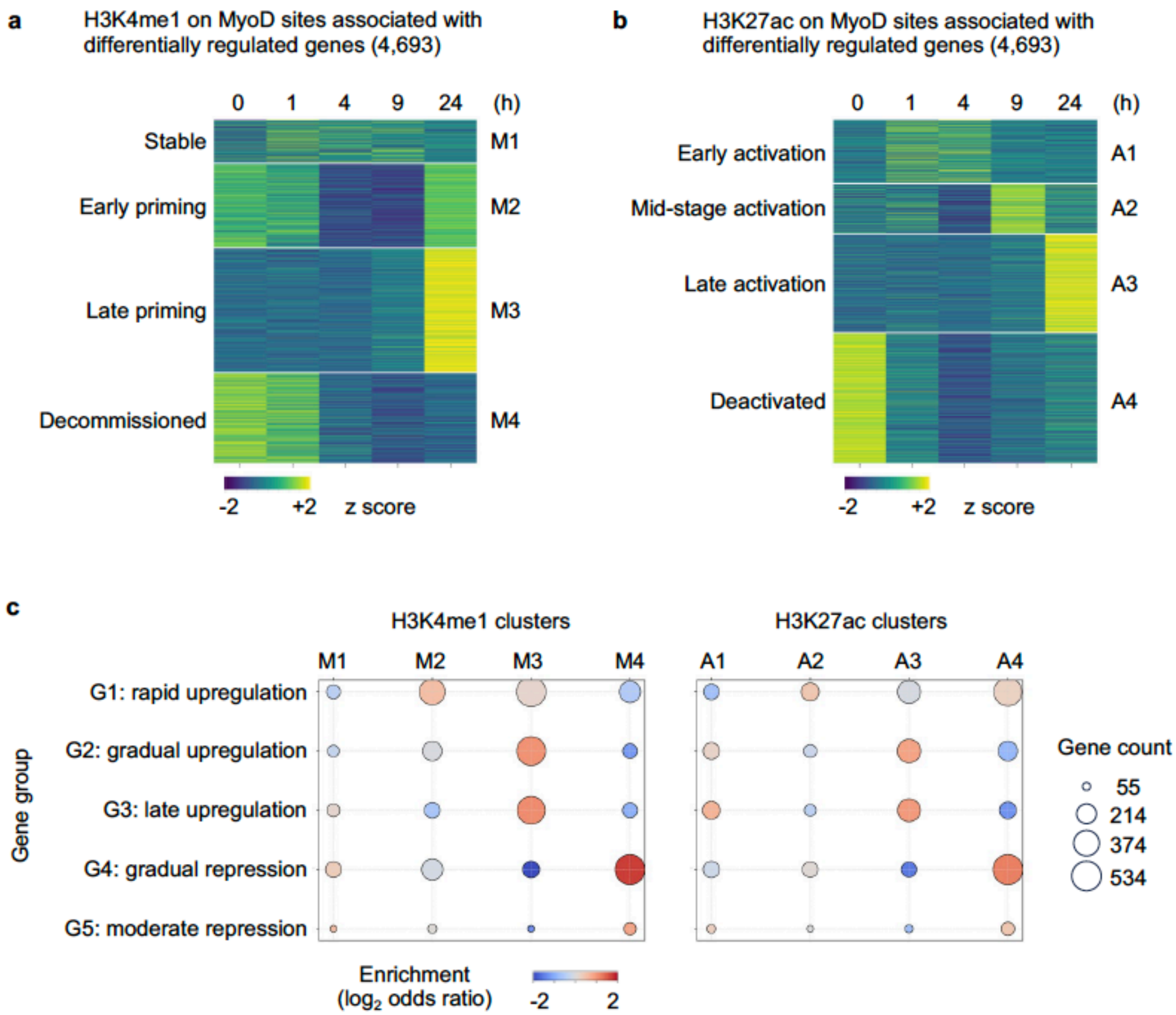

Supplementary Fig. 3. Dynamics of enhancer histone marks upon MyoD translocation during myogenesis.

MyoD-ER-T7 expressing preadipocytes were treated with 4-OHT for 0, 1, 4, 9, and 24 hours, followed by CUT&RUN analysis for MyoD binding and enhancer histone marks H3K4me1 and H3K27ac and nascent RNA-seq analysis. MyoD binding sites were then associated with differentially regulated genes identified from nascent RNA-seq analysis.

(a) Heat maps of clustering depicting dynamics of H3K4me1 on MyoD sites associated with differentially regulated genes. **(b)** Heat maps of clustering depicting dynamics of H3K27ac on MyoD sites associated with differentially regulated genes. **(c)** Dot plots showing the enrichment of genes with different expression dynamics defined in Supplementary Fig. 2a in H3K4me1 clusters (left) and in H3K27ac clusters (right). Dot size and color intensity represent $-\log_{10}$(p-value). These enhancer chromatin dynamics correspond to the MyoD binding sites (Fig. 1) and nascent RNA expression clusters (Supplementary Fig. 2).

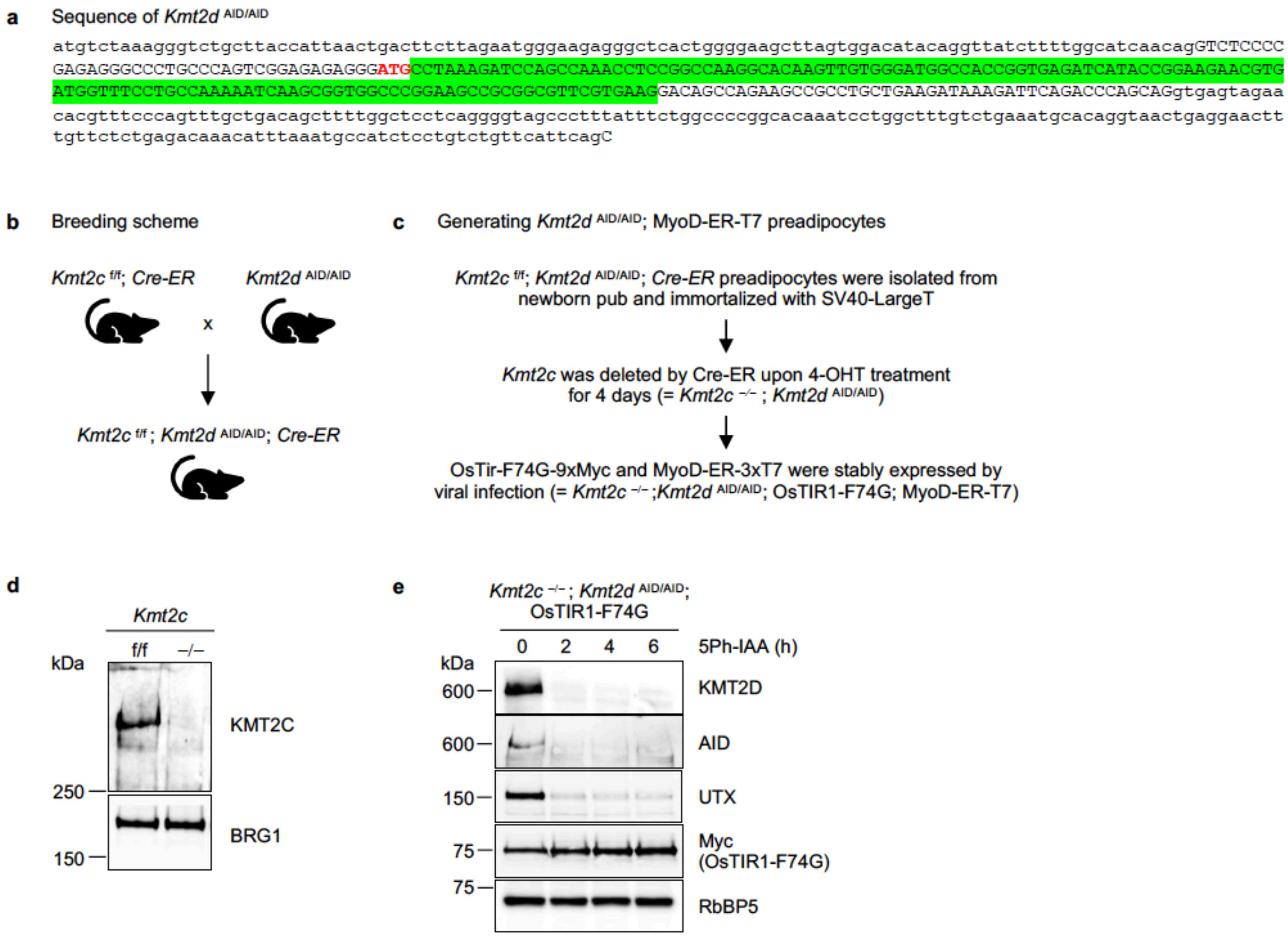

Supplementary Fig. 4. Generation of *Kmt2c* $^{-/-}$; *Kmt2d* $^{AID/AID}$ preadipocytes expressing OsTIR1-F74G-Myc and MyoD-ER-T7.

(a) Sanger sequencing results verifying the integration of 132-bp AID sequence after the start codon (ATG) of *Kmt2d*. **(b)** Breeding scheme for generating *Kmt2c* $^{f/f}$; *Kmt2d* $^{AID/AID}$; *Cre-ER* mice. **(c)** Diagram illustrating the workflow to generate *Kmt2c* $^{-/-}$; *Kmt2d* $^{AID/AID}$ preadipocytes expressing OsTIR1-F74G-Myc and MyoD-ER-T7. **(d)** WB of nuclear extracts confirming KMT2C knockout. Antibodies used were indicated on the right. BRG1 was a loading control. This experiment was repeated independently at least twice with similar results. **(e)** WB of nuclear extracts confirming the depletion of KMT2D in *Kmt2d* $^{AID/AID}$; MyoD-ER-T7 preadipocytes upon 5Ph-IAA treatment for various time durations. Antibodies used were indicated on the right. RbBP5 was used as a sample-processing control across all replicate membranes, and representative signals are shown. This experiment was repeated independently at least twice with similar results.

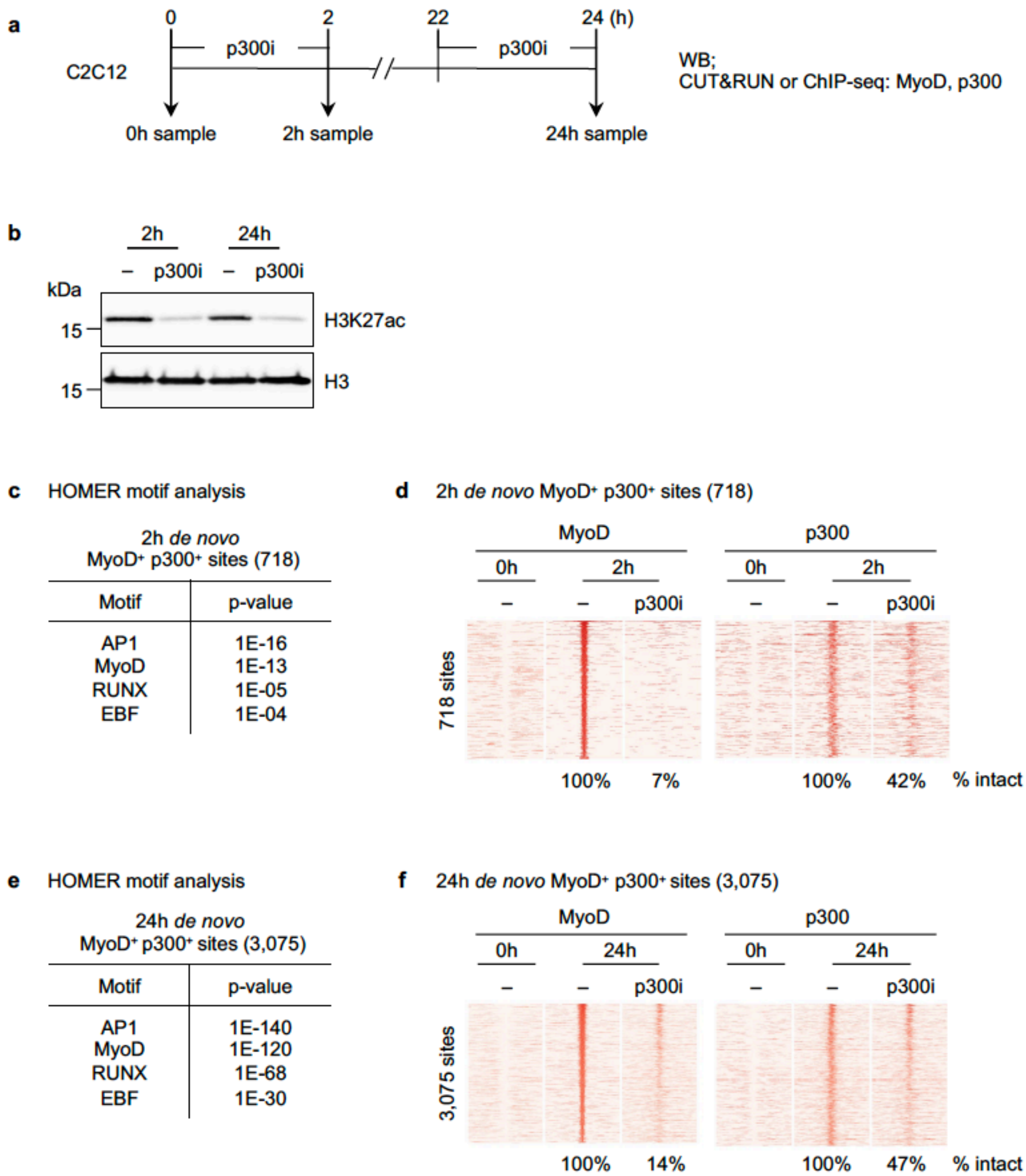

Supplementary Fig. 5. p300 enzymatic activities are required for *de novo* MyoD binding on enhancers in C2C12.

(a) C2C12 myoblasts were subjected to 2h or 24h of myogenesis and 2h of p300 inhibition, with A-485 (p300i) applied for 2h prior to experiments. **(b)** WB of histone extracts for H3K27ac. H3 serves as the loading control. This experiment was repeated independently at least twice with similar results. **(c-d)** Homer motif analysis **(c)** and heat maps for ChIP-seq of MyoD and CUT&RUN of p300 **(d)** on 718 *de novo* MyoD^{+} p300^{+} sites after 2h of differentiation. **(e-f)** Homer motif analysis **(e)** and heat maps for ChIP-seq of MyoD and CUT&RUN of p300 **(f)** on 3,075 *de novo* MyoD^{+} p300^{+} sites after 24h of differentiation.

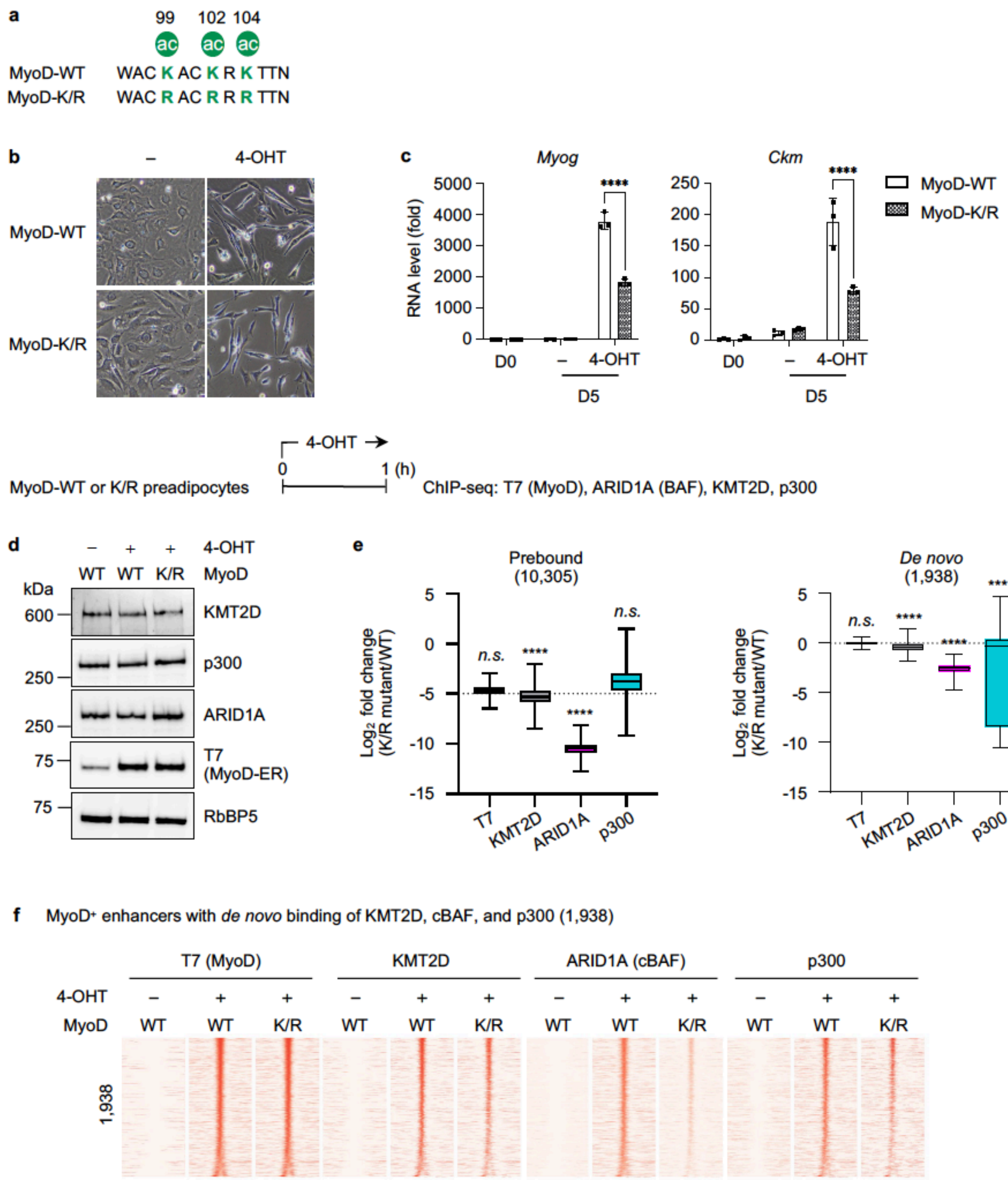

Supplementary Fig. 6. Acetylation of MyoD is dispensable for its binding, but required for chromatin modifier binding on enhancers.

(a) Schematic of MyoD-K/R mutants where K99, K102, K104 were mutated to arginines. **(b-c)** MyoD-WT and MyoD-K/R expressing preadipocytes were treated with 4-OHT and subjected to myogenesis. **(b)** Cell morphology under microscope at D5 of myogenesis. **(c)** qRT-PCR analysis of myogenic markers *Myog* and *Ckm* at D0 and D5 of myogenesis (n = 3 biological replicates). Data represent mean ± SD. Statistical

analysis was performed using a two-way ANOVA with Šídák's multiple comparisons test (**** adjusted p-value ≤ 0.0001). **(d-f)** MyoD-ER-T7 expressing preadipocytes were treated with 4-OHT for 1 hour (h), followed by ChIP-seq analysis. **(d)** WB analysis of nuclear extracts from preadipocytes expressing MyoD-WT or MyoD-K/R and treated with 4-OHT. Antibodies used were indicated on the right. RbBP5 was used as a sample-processing control across all replicate membranes, and representative signals are shown. This experiment was repeated independently at least twice with similar results. **(e)** Box plots showing fold changes of T7 (MyoD), KMT2D, ARID1A (cBAF), and p300 binding intensity on prebound (left) and *de novo* (right) sites between MyoD-K/R and MyoD-WT samples. Statistical significance was determined using a one-sided Wilcoxon signed-rank test. For the significant comparisons shown in the *de novo* group, the exact P values from left to right are 1.26E-241, 2.06E-318, and 1.78E-30. For all box plots, the boxes represent the first and third quartiles, the horizontal line shows the median, and whiskers indicate minimum and maximum. Outliers were not included. **(f)** Heat maps illustrating changes in binding of T7 (MyoD), KMT2D, ARID1A (cBAF), and p300. Heat maps span ± 3kb around MyoD binding sites, and sites are ranked by the intensity of T7 (MyoD) in the MyoD-WT control.

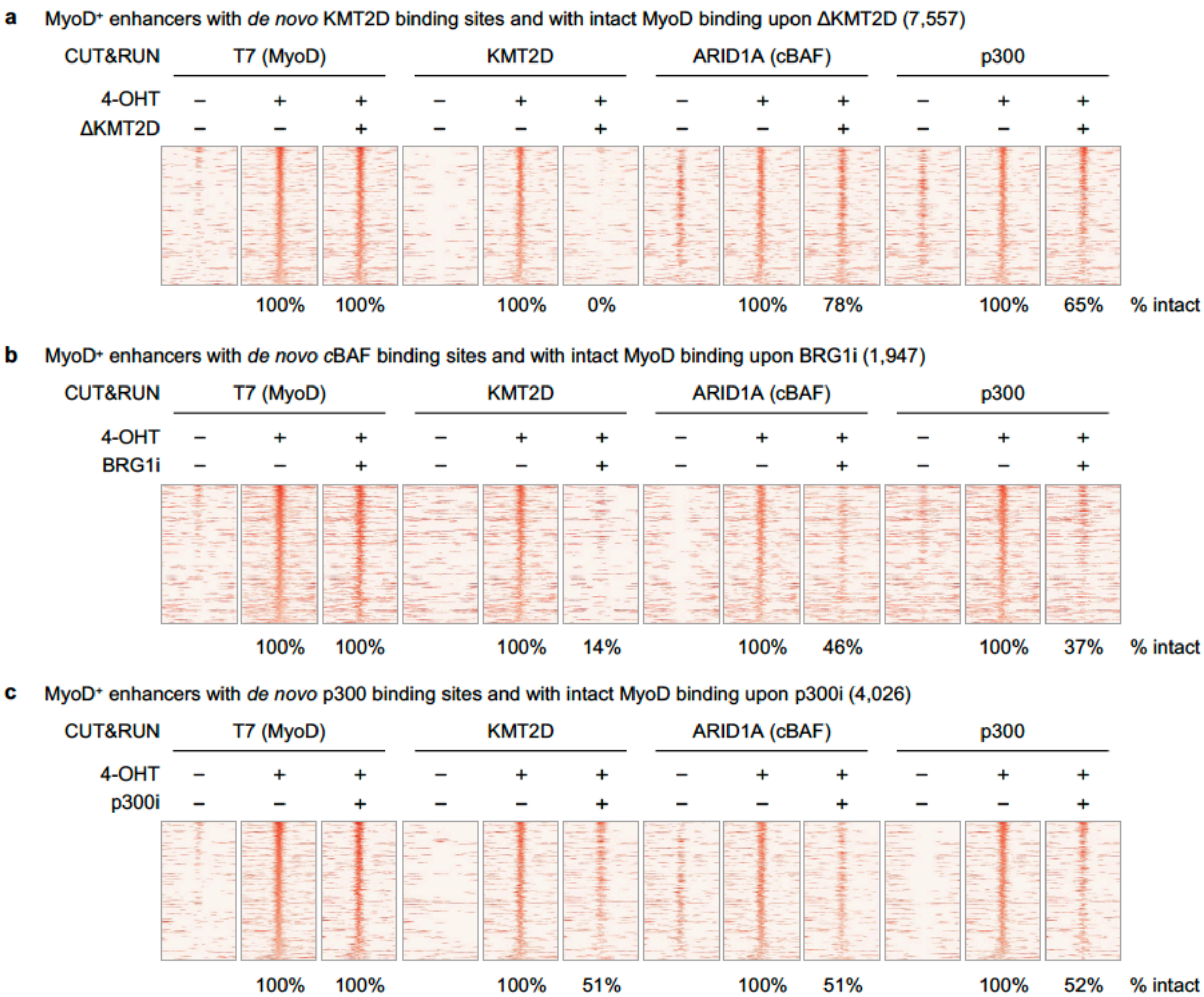

Supplementary Fig. 7. Chromatin modifiers KMT2D, cBAF, and p300 regulate each other's binding on MyoD-intact enhancers.

(a-c) Heat maps for CUT&RUN data on MyoD^{+} enhancers with *de novo* KMT2D binding and with intact MyoD signals upon ΔKMT2D **(a)**, on MyoD^{+} enhancers with *de novo* cBAF binding and with intact MyoD signals upon BRG1i **(b)**, and on MyoD^{+} enhancers with *de novo* p300 binding and with intact MyoD signals upon p300i. All heat maps span ± 3kb around MyoD binding sites, and sites are ranked by the intensity of T7 (MyoD) in the 4OHT-treated control.

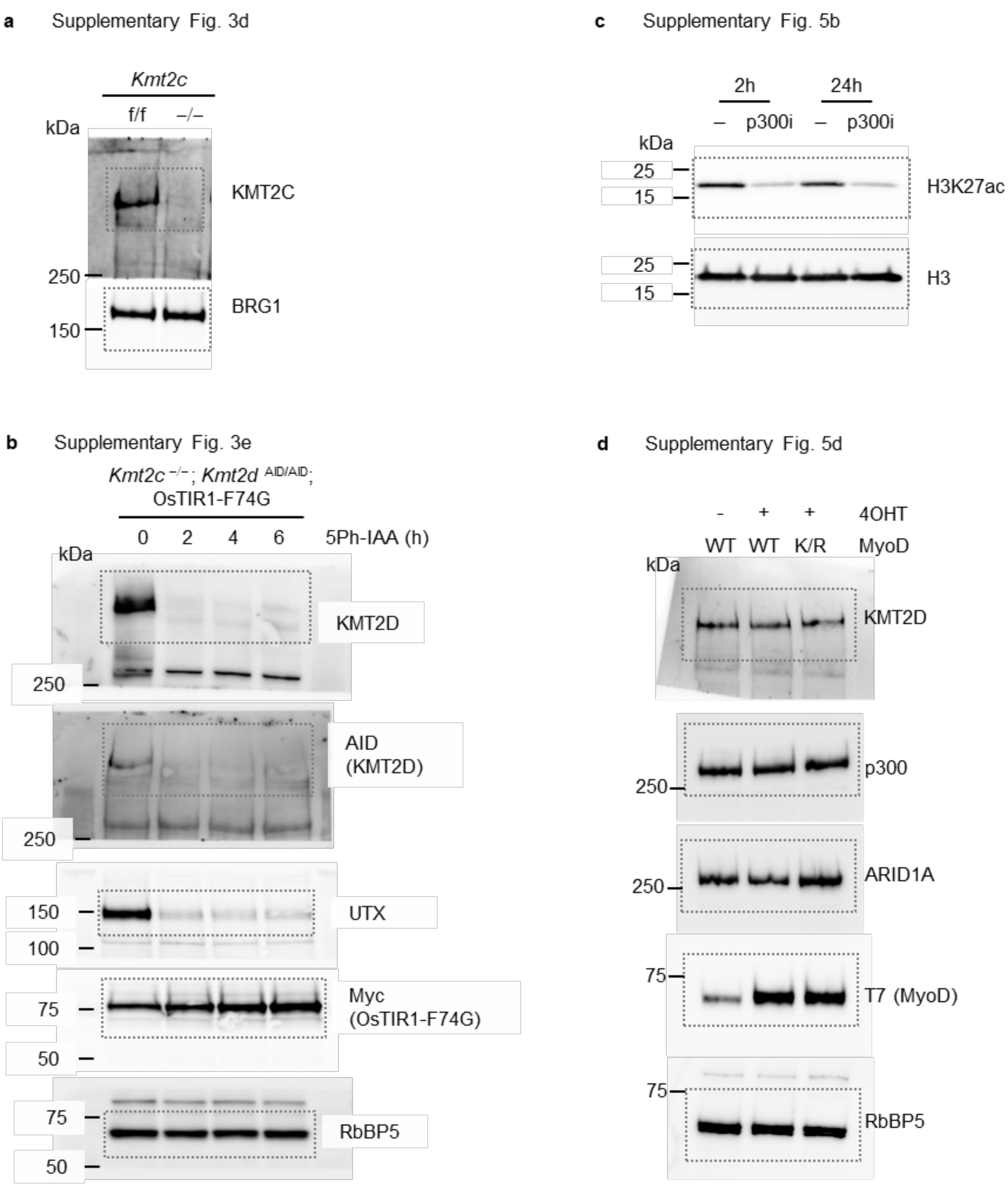

Supplementary Fig. 8. Unprocessed WB images. Uncropped Western blots related to Supplementary Fig. 3d **(a)**, Supplementary Fig. 3e **(b)**, Supplementary Fig. 5b **(c)**, and Supplementary Fig 5d **(c).**
