# Supplementary material for: Chromatin modifiers KMT2D, cBAF, and p300 cooperatively facilitate de novo binding of transcription factors on enhancers
Source: Nat Commun. 2026 Sep 15;17:10816. doi: 10.1038/s41467-026-77757-w (PMC13669397; doi:10.1038/s41467-026-77757-w)
Supplement: Supplementary file 2 [file 41467_2026_77757_MOESM2_ESM.pdf]

nature portfolio

Corresponding author(s): Ji-Eun Lee, Kai Ge

Last updated by author(s): Jul 8, 2026

Reporting Summary

Nature Portfolio wishes to improve the reproducibility of the work that we publish. This form provides structure for consistency and transparency in reporting. For further information on Nature Portfolio policies, see our Editorial Policies and the Editorial Policy Checklist.

Statistics

For all statistical analyses, confirm that the following items are present in the figure legend, table legend, main text, or Methods section.

n/a	Confirmed	
☐	☒	The exact sample size (*n*) for each experimental group/condition, given as a discrete number and unit of measurement
☐	☒	A statement on whether measurements were taken from distinct samples or whether the same sample was measured repeatedly
☐	☒	The statistical test(s) used AND whether they are one- or two-sided<br>*Only common tests should be described solely by name; describe more complex techniques in the Methods section.*
☒	☐	A description of all covariates tested
☒	☐	A description of any assumptions or corrections, such as tests of normality and adjustment for multiple comparisons
☐	☒	A full description of the statistical parameters including central tendency (e.g. means) or other basic estimates (e.g. regression coefficient) AND variation (e.g. standard deviation) or associated estimates of uncertainty (e.g. confidence intervals)
☐	☒	For null hypothesis testing, the test statistic (e.g. *F*, *t*, *r*) with confidence intervals, effect sizes, degrees of freedom and *P* value noted<br>*Give P values as exact values whenever suitable.*
☒	☐	For Bayesian analysis, information on the choice of priors and Markov chain Monte Carlo settings
☒	☐	For hierarchical and complex designs, identification of the appropriate level for tests and full reporting of outcomes
☒	☐	Estimates of effect sizes (e.g. Cohen's *d*, Pearson's *r*), indicating how they were calculated

Our web collection on statistics for biologists contains articles on many of the points above.

Software and code

Policy information about availability of computer code

Data collection	No software was used for data collection.
Data analysis	Software for NGS data processing and analysis included Bowtie2 (v2.3.2), the SICER algorithm (v2), and HOMER (v4.7.2).<br>R packages used for data analysis included gplots (v3.2.0), gglot2 (v3.5.2), DESeq2 (v1.44.0), and Mfuzz (v2.64.0).<br>Software for data visualization and analysis included GraphPad Prism 10 (v10.4.2), the UCSC Genome Browser, and Microsoft Excel (Microsoft 365, v2208).

For manuscripts utilizing custom algorithms or software that are central to the research but not yet described in published literature, software must be made available to editors and reviewers. We strongly encourage code deposition in a community repository (e.g. GitHub). See the Nature Portfolio guidelines for submitting code & software for further information.

Data

Policy information about availability of data

All manuscripts must include a data availability statement. This statement should provide the following information, where applicable:

- Accession codes, unique identifiers, or web links for publicly available datasets
- A description of any restrictions on data availability
- For clinical datasets or third party data, please ensure that the statement adheres to our policy

Data Availability
The ChIP-seq, CUT&RUN, nascent RNA-seq, and ATAC-seq data generated in this study have been deposited in the NCBI Gene Expression Omnibus (GEO) under accession number GSE290849 [https://www.ncbi.nlm.nih.gov/geo/query/acc.cgi?acc=GSE290849] and are available under open public access. Source Data are provided with this paper.

Research involving human participants, their data, or biological material

Policy information about studies with human participants or human data. See also policy information about sex, gender (identity/presentation), and sexual orientation and race, ethnicity and racism.

Reporting on sex and gender	*Use the terms sex (biological attribute) and gender (shaped by social and cultural circumstances) carefully in order to avoid confusing both terms. Indicate if findings apply to only one sex or gender; describe whether sex and gender were considered in study design; whether sex and/or gender was determined based on self-reporting or assigned and methods used.*<br>*Provide in the source data disaggregated sex and gender data, where this information has been collected, and if consent has been obtained for sharing of individual-level data; provide overall numbers in this Reporting Summary. Please state if this information has not been collected.*<br>*Report sex- and gender-based analyses where performed, justify reasons for lack of sex- and gender-based analysis.*
Reporting on race, ethnicity, or other socially relevant groupings	*Please specify the socially constructed or socially relevant categorization variable(s) used in your manuscript and explain why they were used. Please note that such variables should not be used as proxies for other socially constructed/relevant variables (for example, race or ethnicity should not be used as a proxy for socioeconomic status).*<br>*Provide clear definitions of the relevant terms used, how they were provided (by the participants/respondents, the researchers, or third parties), and the method(s) used to classify people into the different categories (e.g. self-report, census or administrative data, social media data, etc.)*<br>*Please provide details about how you controlled for confounding variables in your analyses.*
Population characteristics	*Describe the covariate-relevant population characteristics of the human research participants (e.g. age, genotypic information, past and current diagnosis and treatment categories). If you filled out the behavioural & social sciences study design questions and have nothing to add here, write "See above."*
Recruitment	*Describe how participants were recruited. Outline any potential self-selection bias or other biases that may be present and how these are likely to impact results.*
Ethics oversight	*Identify the organization(s) that approved the study protocol.*

Note that full information on the approval of the study protocol must also be provided in the manuscript.

Field-specific reporting

Please select the one below that is the best fit for your research. If you are not sure, read the appropriate sections before making your selection.

☒ Life sciences ☐ Behavioural & social sciences ☐ Ecological, evolutionary & environmental sciences

For a reference copy of the document with all sections, see nature.com/documents/nr-reporting-summary-flat.pdf

Life sciences study design

All studies must disclose on these points even when the disclosure is negative.

Sample size	No statistical methods were used to predetermine sample size. Sample sizes were determined based on published literature and previous experience.
Data exclusions	No data were excluded.
Replication	All results from representative experiments were obtained from at least two independent biological replicates. Replicates generally showed high correlation.
Randomization	No randomization.
Blinding	Investigators were not blinded during in vitro experiments because these experiments required frequent intervention to maintain cell lines

with different genotypes.

Reporting for specific materials, systems and methods

We require information from authors about some types of materials, experimental systems and methods used in many studies. Here, indicate whether each material, system or method listed is relevant to your study. If you are not sure if a list item applies to your research, read the appropriate section before selecting a response.

Materials & experimental systems

n/a	Involved in the study
	[x] Antibodies
	[x] Eukaryotic cell lines
[x]	Palaeontology and archaeology
	[x] Animals and other organisms
[x]	Clinical data
[x]	Dual use research of concern
[x]	Plants

Methods

n/a	Involved in the study
	[x] ChIP-seq
[x]	Flow cytometry
[x]	MRI-based neuroimaging

Antibodies

Antibodies used

Primary antibodies: Anti-T7 tag (D9E1X; 13246S; 1:1000 for WB and 1 µl per C&R), anti-ARID1A/BAF250A (D2A8U; 12354, 1:1000 for WB and 1 µl per C&R), and anti-p300 (D8Z4E; 86377, 1:1000 for WB and 1 µl per C&R) were obtained from Cell Signaling Technology. Anti-H3K27ac (ab4729; 1:1000 for WB and 0.5 µg per C&R), anti-BRG1 (ab110641; 1:5000 for WB), and anti-H3 (ab1791; 1:8000 for WB) were obtained from Abcam. Anti-H3K4me1 (13-0040; 1:2000 for WB and 0.5 µg per C&R), and rabbit control IgG (13-0042K) were obtained from EpiCypher. Anti-p300 (C-20; sc-585, 1:1000 for WB), anti-MyoD (5.8A; sc-32758, 1:1000 for WB and 4 µg per ChIP), anti-GR (M-20; sc-100041, 1:1000 for WB and 4 µg per ChIP), and anti-Myc (9E10; sc-40, 1:5000 for WB) were obtained from Santa Cruz Biotechnology. Anti-KMT2C (#3; 1:800 for WB), anti-KMT2D (#3; 1:3000 for WB, 2 µg per C&R), and anti-UTX (#2; 1:3000 for WB) were home-made. Anti-RbBP5 (A300-109A; 1:5000 for WB) was obtained from Bethyl Laboratories. Anti-mAID Tag (NBP2-89163, 1:1000 for WB) was obtained from Novus Biologicals.

Secondary antibodies: Anti-rabbit IgG, HRP-linked (7074S; 1:2000 for WB) and anti-mouse IgG, HRP-linked (7076S; 1:2000 for WB) were obtained from Cell Signaling Technology.

Validation

All antibodies were validated by the manufacturer or used in previous studies.
Anti-ARID1A/BAF250A (D2A8U, Cell Signaling Technology): validated by manufacturer, https://www.cellsignal.com/products/primary-antibodies/arid1a-baf250a-d2a8u-rabbit-mab/12354
Anti-BRG1 (ab110641, Abcam): validated by manufacturer, https://www.abcam.com/brg1-antibody-epncir111a-ab110641
Anti-CBP (7389S, Cell Signaling Technology): validated by manufacturer, https://www.cellsignal.com/products/primary-antibodies/cbp-d6c5-rabbit-mab/7389
Anti-GR (M-20, Santa Cruz): https://pubmed.ncbi.nlm.nih.gov/27777311/
Anti-H3 (ab1791, Abcam): validated by manufacturer, https://www.abcam.com/histone-h3-antibody-nuclear-marker-and-chip-grade-ab1791
Anti-H3K27ac (ab4729, Abcam): validated by manufacturer, https://www.abcam.com/histone-h3-acetyl-k27-antibody-chip-grade-ab4729
Anti-H3K4me1 (13-0040, EpiCypher): validated by manufacturer, https://www.epicypher.com/products/antibodies/snap-chip-certified-antibodies/histone-h3k4me1-antibody-snap-chip-certified
Anti-KMT2C (#3, home-made): https://pubmed.ncbi.nlm.nih.gov/17500065/
Anti-KMT2D (#3, home-made): https://pubmed.ncbi.nlm.nih.gov/17500065/
Anti-mAID Tag (NBP2-89163, Novus Biologicals): validated by manufacturer, https://www.novusbio.com/products/maid-tag-antibody_nbp2-89163sc-1004
Anti-MyoD (G-1, Santa Cruz): validated by manufacturer, https://www.scbt.com/p/myod-antibody-g-1
Anti-p300 (D8Z4E, Cell Signaling Technology): validated by manufacturer, https://www.cellsignal.com/products/primary-antibodies/p300-d8z4e-rabbit-mab/86377
Anti-RbBP5 (A300-109A, Bethyl Laboratories): validated by manufacturer, https://www.fortislife.com/products/primary-antibodies/rabbit-anti-rbbp5-antibody/BETHYL-A300-109
Anti-T7 tag (13246S, Cell Signaling Technology): https://pubmed.ncbi.nlm.nih.gov/33888555/
Anti-UTX (#2, home-made): https://pubmed.ncbi.nlm.nih.gov/18003914/
Anti-Rabbit IgG, HRP-linked (7074S, Cell Signaling Technology): validated by manufacturer, https://www.cellsignal.com/products/secondary-antibodies/anti-rabbit-igg-hrp-linked-antibody/7074
Anti-Mouse IgG, HRP-linked (7076S, Cell Signaling Technology): validated by manufacturer, https://www.cellsignal.com/products/secondary-antibodies/anti-mouse-igg-hrp-linked-antibody/7076

Eukaryotic cell lines

Policy information about cell lines and Sex and Gender in Research

Cell line source(s)	The C2C12 myoblast cell line was obtained from ATCC. The Brg1-AID/AID preadipocyte cell line was previously described (Park et al., 2021; https://pubmed.ncbi.nlm.nih.gov/33712604/). The Kmt2c−/−;Kmt2d-AID/AID preadipocyte cell line was generated in this study.
Authentication	None of the cell lines used were authenticated.
Mycoplasma contamination	Cell lines were not tested for mycoplasma contamination but no indication of contamination was observed.
Commonly misidentified lines (See ICLAC register)	No commonly misidentified cell lines were used.

Animals and other research organisms

Policy information about studies involving animals; ARRIVE guidelines recommended for reporting animal research, and Sex and Gender in Research

Laboratory animals	To generate Kmt2d AID/AID mice, a 132bp DNA fragment encoding AID tag was inserted after the start codon located on exon 2 of Kmt2d by CRISPR/Cas9-mediated homology-directed repair. Kmt2d AID/AID mice were crossed with Kmt2c f/f; Cre-ER mice to obtain Kmt2c f/f; Kmt2d AID/AID; Cre-ER mice.
Wild animals	No wild animal was used in this research.
Reporting on sex	Both male and female mice were used in this study.
Field-collected samples	No field-collected samples were used.
Ethics oversight	All mouse work was approved by the Animal Care and Use Committee of NIDDK, NIH.

Note that full information on the approval of the study protocol must also be provided in the manuscript.

Plants

Seed stocks	*Report on the source of all seed stocks or other plant material used. If applicable, state the seed stock centre and catalogue number. If plant specimens were collected from the field, describe the collection location, date and sampling procedures.*
Novel plant genotypes	*Describe the methods by which all novel plant genotypes were produced. This includes those generated by transgenic approaches, gene editing, chemical/radiation-based mutagenesis and hybridization. For transgenic lines, describe the transformation method, the number of independent lines analyzed and the generation upon which experiments were performed. For gene-edited lines, describe the editor used, the endogenous sequence targeted for editing, the targeting guide RNA sequence (if applicable) and how the editor was applied.*
Authentication	*Describe any authentication procedures for each seed stock used or novel genotype generated. Describe any experiments used to assess the effect of a mutation and, where applicable, how potential secondary effects (e.g. second site T-DNA insertions, mosiacism, off-target gene editing) were examined.*

ChIP-seq

Data deposition

☒ Confirm that both raw and final processed data have been deposited in a public database such as GEO.

☒ Confirm that you have deposited or provided access to graph files (e.g. BED files) for the called peaks.

Data access links *May remain private before publication.*	https://www.ncbi.nlm.nih.gov/geo/query/acc.cgi?acc=GSE290849
Files in database submission	Brg1-AID_CnR_MyoD-ER_4OHT_0h_IgG_R1_001.fastq.gz<br>Brg1-AID_CnR_MyoD-ER_4OHT_0h_IgG_R2_001.fastq.gz<br>Brg1-AID_CnR_MyoD-ER_4OHT_0h_K27ac_R1_001.fastq.gz<br>Brg1-AID_CnR_MyoD-ER_4OHT_0h_K27ac_R2_001.fastq.gz<br>Brg1-AID_CnR_MyoD-ER_4OHT_0h_K4me1_R1_001.fastq.gz<br>Brg1-AID_CnR_MyoD-ER_4OHT_0h_K4me1_R2_001.fastq.gz<br>Brg1-AID_CnR_MyoD-ER_4OHT_0h_T7_R1_001.fastq.gz<br>Brg1-AID_CnR_MyoD-ER_4OHT_0h_T7_R2_001.fastq.gz<br>Brg1-AID_CnR_MyoD-ER_4OHT_1h_IgG_R1_001.fastq.gz<br>Brg1-AID_CnR_MyoD-ER_4OHT_1h_IgG_R2_001.fastq.gz<br>Brg1-AID_CnR_MyoD-ER_4OHT_1h_K27ac_R1_001.fastq.gz<br>Brg1-AID_CnR_MyoD-ER_4OHT_1h_K27ac_R2_001.fastq.gz

Brg1-AID_CnR_MyoD-ER_4OHT_1h_K4me1_R1_001.fastq.gz
Brg1-AID_CnR_MyoD-ER_4OHT_1h_K4me1_R2_001.fastq.gz
Brg1-AID_CnR_MyoD-ER_4OHT_1h_T7_R1_001.fastq.gz
Brg1-AID_CnR_MyoD-ER_4OHT_1h_T7_R2_001.fastq.gz
Brg1-AID_CnR_MyoD-ER_4OHT_4h_IgG_R1_001.fastq.gz
Brg1-AID_CnR_MyoD-ER_4OHT_4h_IgG_R2_001.fastq.gz
Brg1-AID_CnR_MyoD-ER_4OHT_4h_K27ac_R1_001.fastq.gz
Brg1-AID_CnR_MyoD-ER_4OHT_4h_K27ac_R2_001.fastq.gz
Brg1-AID_CnR_MyoD-ER_4OHT_4h_K4me1_R1_001.fastq.gz
Brg1-AID_CnR_MyoD-ER_4OHT_4h_K4me1_R2_001.fastq.gz
Brg1-AID_CnR_MyoD-ER_4OHT_4h_T7_R1_001.fastq.gz
Brg1-AID_CnR_MyoD-ER_4OHT_4h_T7_R2_001.fastq.gz
Brg1-AID_CnR_MyoD-ER_4OHT_9h_IgG_R1_001.fastq.gz
Brg1-AID_CnR_MyoD-ER_4OHT_9h_IgG_R2_001.fastq.gz
Brg1-AID_CnR_MyoD-ER_4OHT_9h_K27ac_R1_001.fastq.gz
Brg1-AID_CnR_MyoD-ER_4OHT_9h_K27ac_R2_001.fastq.gz
Brg1-AID_CnR_MyoD-ER_4OHT_9h_K4me1_R1_001.fastq.gz
Brg1-AID_CnR_MyoD-ER_4OHT_9h_K4me1_R2_001.fastq.gz
Brg1-AID_CnR_MyoD-ER_4OHT_9h_T7_R1_001.fastq.gz
Brg1-AID_CnR_MyoD-ER_4OHT_9h_T7_R2_001.fastq.gz
Brg1-AID_CnR_MyoD-ER_4OHT_24h_IgG_R1_001.fastq.gz
Brg1-AID_CnR_MyoD-ER_4OHT_24h_IgG_R2_001.fastq.gz
Brg1-AID_CnR_MyoD-ER_4OHT_24h_K27ac_R1_001.fastq.gz
Brg1-AID_CnR_MyoD-ER_4OHT_24h_K27ac_R2_001.fastq.gz
Brg1-AID_CnR_MyoD-ER_4OHT_24h_K4me1_R1_001.fastq.gz
Brg1-AID_CnR_MyoD-ER_4OHT_24h_K4me1_R2_001.fastq.gz
Brg1-AID_CnR_MyoD-ER_4OHT_24h_T7_R1_001.fastq.gz
Brg1-AID_CnR_MyoD-ER_4OHT_24h_T7_R2_001.fastq.gz
Brg1-AID_CnR_MyoD-ER_4OHT_72h_IgG_R1_001.fastq.gz
Brg1-AID_CnR_MyoD-ER_4OHT_72h_IgG_R2_001.fastq.gz
Brg1-AID_CnR_MyoD-ER_4OHT_72h_K27ac_R1_001.fastq.gz
Brg1-AID_CnR_MyoD-ER_4OHT_72h_K27ac_R2_001.fastq.gz
Brg1-AID_CnR_MyoD-ER_4OHT_72h_K4me1_R1_001.fastq.gz
Brg1-AID_CnR_MyoD-ER_4OHT_72h_K4me1_R2_001.fastq.gz
Brg1-AID_CnR_MyoD-ER_4OHT_72h_T7_R1_001.fastq.gz
Brg1-AID_CnR_MyoD-ER_4OHT_72h_T7_R2_001.fastq.gz
Mll4-AID_MyoD-ER_unt_ARID1A_S32_L002_R1_001.fastq.gz
Mll4-AID_MyoD-ER_unt_ARID1A_S32_L002_R2_001.fastq.gz
Mll4-AID_MyoD-ER_unt_IgG_S17_L002_R1_001.fastq.gz
Mll4-AID_MyoD-ER_unt_IgG_S17_L002_R2_001.fastq.gz
Mll4-AID_MyoD-ER_unt_MLL4_S27_L002_R1_001.fastq.gz
Mll4-AID_MyoD-ER_unt_MLL4_S27_L002_R2_001.fastq.gz
Mll4-AID_MyoD-ER_unt_P300_S37_L002_R1_001.fastq.gz
Mll4-AID_MyoD-ER_unt_P300_S37_L002_R2_001.fastq.gz
Mll4-AID_MyoD-ER_unt_T7_S22_L002_R1_001.fastq.gz
Mll4-AID_MyoD-ER_unt_T7_S22_L002_R2_001.fastq.gz
Mll4-AID_MyoD-ER_4OHT_ARID1A_S33_L002_R1_001.fastq.gz
Mll4-AID_MyoD-ER_4OHT_ARID1A_S33_L002_R2_001.fastq.gz
Mll4-AID_MyoD-ER_4OHT_IgG_S18_L002_R1_001.fastq.gz
Mll4-AID_MyoD-ER_4OHT_IgG_S18_L002_R2_001.fastq.gz
Mll4-AID_MyoD-ER_4OHT_MLL4_S28_L002_R1_001.fastq.gz
Mll4-AID_MyoD-ER_4OHT_MLL4_S28_L002_R2_001.fastq.gz
Mll4-AID_MyoD-ER_4OHT_P300_S38_L002_R1_001.fastq.gz
Mll4-AID_MyoD-ER_4OHT_P300_S38_L002_R2_001.fastq.gz
Mll4-AID_MyoD-ER_4OHT_T7_S23_L002_R1_001.fastq.gz
Mll4-AID_MyoD-ER_4OHT_T7_S23_L002_R2_001.fastq.gz
Mll4-AID_MyoD-ER_IAA_4OHT_ARID1A_S35_L002_R1_001.fastq.gz
Mll4-AID_MyoD-ER_IAA_4OHT_ARID1A_S35_L002_R2_001.fastq.gz
Mll4-AID_MyoD-ER_IAA_4OHT_IgG_S20_L002_R1_001.fastq.gz
Mll4-AID_MyoD-ER_IAA_4OHT_IgG_S20_L002_R2_001.fastq.gz
Mll4-AID_MyoD-ER_IAA_4OHT_MLL4_S30_L002_R1_001.fastq.gz
Mll4-AID_MyoD-ER_IAA_4OHT_MLL4_S30_L002_R2_001.fastq.gz
Mll4-AID_MyoD-ER_IAA_4OHT_P300_S40_L002_R1_001.fastq.gz
Mll4-AID_MyoD-ER_IAA_4OHT_P300_S40_L002_R2_001.fastq.gz
Mll4-AID_MyoD-ER_IAA_4OHT_T7_S25_L002_R1_001.fastq.gz
Mll4-AID_MyoD-ER_IAA_4OHT_T7_S25_L002_R2_001.fastq.gz
Mll4-AID_MyoD-ER_BRM_4OHT_ARID1A_S34_L002_R1_001.fastq.gz
Mll4-AID_MyoD-ER_BRM_4OHT_ARID1A_S34_L002_R2_001.fastq.gz
Mll4-AID_MyoD-ER_BRM_4OHT_IgG_S19_L002_R1_001.fastq.gz

Mll4-AID_MyoD-ER_BRM_4OHT_IgG_S19_L002_R2_001.fastq.gz
Mll4-AID_MyoD-ER_BRM_4OHT_MLL4_S29_L002_R1_001.fastq.gz
Mll4-AID_MyoD-ER_BRM_4OHT_MLL4_S29_L002_R2_001.fastq.gz
Mll4-AID_MyoD-ER_BRM_4OHT_P300_S39_L002_R1_001.fastq.gz
Mll4-AID_MyoD-ER_BRM_4OHT_P300_S39_L002_R2_001.fastq.gz
Mll4-AID_MyoD-ER_BRM_4OHT_T7_S24_L002_R1_001.fastq.gz
Mll4-AID_MyoD-ER_BRM_4OHT_T7_S24_L002_R2_001.fastq.gz
Mll4-AID_MyoD-ER_A485_4OHT_ARID1A_S36_L002_R1_001.fastq.gz
Mll4-AID_MyoD-ER_A485_4OHT_ARID1A_S36_L002_R2_001.fastq.gz
Mll4-AID_MyoD-ER_A485_4OHT_IgG_S21_L002_R1_001.fastq.gz
Mll4-AID_MyoD-ER_A485_4OHT_IgG_S21_L002_R2_001.fastq.gz
Mll4-AID_MyoD-ER_A485_4OHT_MLL4_S31_L002_R1_001.fastq.gz
Mll4-AID_MyoD-ER_A485_4OHT_MLL4_S31_L002_R2_001.fastq.gz
Mll4-AID_MyoD-ER_A485_4OHT_P300_S41_L002_R1_001.fastq.gz
Mll4-AID_MyoD-ER_A485_4OHT_P300_S41_L002_R2_001.fastq.gz
Mll4-AID_MyoD-ER_A485_4OHT_T7_S26_L002_R1_001.fastq.gz
Mll4-AID_MyoD-ER_A485_4OHT_T7_S26_L002_R2_001.fastq.gz
Mll4_AID_MyoD_ER_unt_ARID1A_S45_L008_R1_001.fastq.gz
Mll4_AID_MyoD_ER_unt_ARID1A_S45_L008_R2_001.fastq.gz
Mll4_AID_MyoD_ER_unt_IgG_S37_L008_R1_001.fastq.gz
Mll4_AID_MyoD_ER_unt_IgG_S37_L008_R2_001.fastq.gz
Mll4_AID_MyoD_ER_unt_MLL4_S49_L008_R1_001.fastq.gz
Mll4_AID_MyoD_ER_unt_MLL4_S49_L008_R2_001.fastq.gz
Mll4_AID_MyoD_ER_unt_T7_S41_L008_R1_001.fastq.gz
Mll4_AID_MyoD_ER_unt_T7_S41_L008_R2_001.fastq.gz
Mll4_AID_MyoD_ER_unt_p300_CST_S53_L008_R1_001.fastq.gz
Mll4_AID_MyoD_ER_unt_p300_CST_S53_L008_R2_001.fastq.gz
Mll4_AID_MyoD_ER_4OHT_ARID1A_S46_L008_R1_001.fastq.gz
Mll4_AID_MyoD_ER_4OHT_ARID1A_S46_L008_R2_001.fastq.gz
Mll4_AID_MyoD_ER_4OHT_IgG_S38_L008_R1_001.fastq.gz
Mll4_AID_MyoD_ER_4OHT_IgG_S38_L008_R2_001.fastq.gz
Mll4_AID_MyoD_ER_4OHT_MLL4_S50_L008_R1_001.fastq.gz
Mll4_AID_MyoD_ER_4OHT_MLL4_S50_L008_R2_001.fastq.gz
Mll4_AID_MyoD_ER_4OHT_T7_S42_L008_R1_001.fastq.gz
Mll4_AID_MyoD_ER_4OHT_T7_S42_L008_R2_001.fastq.gz
Mll4_AID_MyoD_ER_4OHT_p300_CST_S54_L008_R1_001.fastq.gz
Mll4_AID_MyoD_ER_4OHT_p300_CST_S54_L008_R2_001.fastq.gz
Mll4_AID_MyoD_ER_p300_inh_4OHT_ARID1A_S47_L008_R1_001.fastq.gz
Mll4_AID_MyoD_ER_p300_inh_4OHT_ARID1A_S47_L008_R2_001.fastq.gz
Mll4_AID_MyoD_ER_p300_inh_4OHT_IgG_S39_L008_R1_001.fastq.gz
Mll4_AID_MyoD_ER_p300_inh_4OHT_IgG_S39_L008_R2_001.fastq.gz
Mll4_AID_MyoD_ER_p300_inh_4OHT_MLL4_S51_L008_R1_001.fastq.gz
Mll4_AID_MyoD_ER_p300_inh_4OHT_MLL4_S51_L008_R2_001.fastq.gz
Mll4_AID_MyoD_ER_p300_inh_4OHT_T7_S43_L008_R1_001.fastq.gz
Mll4_AID_MyoD_ER_p300_inh_4OHT_T7_S43_L008_R2_001.fastq.gz
Mll4_AID_MyoD_ER_p300_inh_4OHT_p300_CST_S55_L008_R1_001.fastq.gz
Mll4_AID_MyoD_ER_p300_inh_4OHT_p300_CST_S55_L008_R2_001.fastq.gz
Mll4_AID_MyoD_ER_p300_deg_4OHT_ARID1A_S48_L008_R1_001.fastq.gz
Mll4_AID_MyoD_ER_p300_deg_4OHT_ARID1A_S48_L008_R2_001.fastq.gz
Mll4_AID_MyoD_ER_p300_deg_4OHT_IgG_S40_L008_R1_001.fastq.gz
Mll4_AID_MyoD_ER_p300_deg_4OHT_IgG_S40_L008_R2_001.fastq.gz
Mll4_AID_MyoD_ER_p300_deg_4OHT_MLL4_S52_L008_R1_001.fastq.gz
Mll4_AID_MyoD_ER_p300_deg_4OHT_MLL4_S52_L008_R2_001.fastq.gz
Mll4_AID_MyoD_ER_p300_deg_4OHT_T7_S44_L008_R1_001.fastq.gz
Mll4_AID_MyoD_ER_p300_deg_4OHT_T7_S44_L008_R2_001.fastq.gz
Mll4_AID_MyoD_ER_p300_deg_4OHT_p300_CST_S56_L008_R1_001.fastq.gz
Mll4_AID_MyoD_ER_p300_deg_4OHT_p300_CST_S56_L008_R2_001.fastq.gz
Mll4-AID_MyoD-ER_unt_ATAC_S1_L001_R1_001.fastq.gz
Mll4-AID_MyoD-ER_unt_ATAC_S1_L001_R2_001.fastq.gz
Mll4-AID_MyoD-ER_4OHT_ATAC_S2_L001_R1_001.fastq.gz
Mll4-AID_MyoD-ER_4OHT_ATAC_S2_L001_R2_001.fastq.gz
Mll4-AID_MyoD-ER_BRM_4OHT_ATAC_S3_L001_R1_001.fastq.gz
Mll4-AID_MyoD-ER_BRM_4OHT_ATAC_S3_L001_R2_001.fastq.gz
Mll4-AID_MyoD-ER_A485_4OHT_S5_L001_R1_001.fastq.gz
Mll4-AID_MyoD-ER_A485_4OHT_S5_L001_R2_001.fastq.gz
Mll4-AID_par_Dex_ARID1A_S84_L002_R1_001.fastq.gz
Mll4-AID_par_Dex_ARID1A_S84_L002_R2_001.fastq.gz
Mll4-AID_par_Dex_GR_M20_S74_L002_R1_001.fastq.gz
Mll4-AID_par_Dex_GR_M20_S74_L002_R2_001.fastq.gz

Mll4-AID_par_Dex_KMT2D_S79_L002_R1_001.fastq.gz
Mll4-AID_par_Dex_KMT2D_S79_L002_R2_001.fastq.gz
Mll4-AID_par_Dex_input_S69_L002_R1_001.fastq.gz
Mll4-AID_par_Dex_input_S69_L002_R2_001.fastq.gz
Mll4-AID_par_Dex_p300_S89_L002_R1_001.fastq.gz
Mll4-AID_par_Dex_p300_S89_L002_R2_001.fastq.gz
Mll4-AID_par_dMLL4-Dex_ARID1A_S85_L002_R1_001.fastq.gz
Mll4-AID_par_dMLL4-Dex_ARID1A_S85_L002_R2_001.fastq.gz
Mll4-AID_par_dMLL4-Dex_GR_M20_S75_L002_R1_001.fastq.gz
Mll4-AID_par_dMLL4-Dex_GR_M20_S75_L002_R2_001.fastq.gz
Mll4-AID_par_dMLL4-Dex_KMT2D_S80_L002_R1_001.fastq.gz
Mll4-AID_par_dMLL4-Dex_KMT2D_S80_L002_R2_001.fastq.gz
Mll4-AID_par_dMLL4-Dex_input_S70_L002_R1_001.fastq.gz
Mll4-AID_par_dMLL4-Dex_input_S70_L002_R2_001.fastq.gz
Mll4-AID_par_dMLL4-Dex_p300_S90_L002_R1_001.fastq.gz
Mll4-AID_par_dMLL4-Dex_p300_S90_L002_R2_001.fastq.gz
Mll4-AID_par_BRG1i-Dex_ARID1A_S86_L002_R1_001.fastq.gz
Mll4-AID_par_BRG1i-Dex_ARID1A_S86_L002_R2_001.fastq.gz
Mll4-AID_par_BRG1i-Dex_GR_M20_S76_L002_R1_001.fastq.gz
Mll4-AID_par_BRG1i-Dex_GR_M20_S76_L002_R2_001.fastq.gz
Mll4-AID_par_BRG1i-Dex_KMT2D_S81_L002_R1_001.fastq.gz
Mll4-AID_par_BRG1i-Dex_KMT2D_S81_L002_R2_001.fastq.gz
Mll4-AID_par_BRG1i-Dex_input_S71_L002_R1_001.fastq.gz
Mll4-AID_par_BRG1i-Dex_input_S71_L002_R2_001.fastq.gz
Mll4-AID_par_BRG1i-Dex_p300_S91_L002_R1_001.fastq.gz
Mll4-AID_par_BRG1i-Dex_p300_S91_L002_R2_001.fastq.gz
Mll4-AID_par_p300i-Dex_ARID1A_S87_L002_R1_001.fastq.gz
Mll4-AID_par_p300i-Dex_ARID1A_S87_L002_R2_001.fastq.gz
Mll4-AID_par_p300i-Dex_GR_M20_S77_L002_R1_001.fastq.gz
Mll4-AID_par_p300i-Dex_GR_M20_S77_L002_R2_001.fastq.gz
Mll4-AID_par_p300i-Dex_KMT2D_S82_L002_R1_001.fastq.gz
Mll4-AID_par_p300i-Dex_KMT2D_S82_L002_R2_001.fastq.gz
Mll4-AID_par_p300i-Dex_input_S72_L002_R1_001.fastq.gz
Mll4-AID_par_p300i-Dex_input_S72_L002_R2_001.fastq.gz
Mll4-AID_par_p300i-Dex_p300_S92_L002_R1_001.fastq.gz
Mll4-AID_par_p300i-Dex_p300_S92_L002_R2_001.fastq.gz
Brg1-AID_CnR_MyoD-ER_4OHT_0h_K27ac_sorted-W200-G200-FDR0.001-islandfiltered-normalized.wig
Brg1-AID_CnR_MyoD-ER_4OHT_0h_K4me1_sorted-W200-G200-FDR0.001-islandfiltered-normalized.wig
Brg1-AID_CnR_MyoD-ER_4OHT_0h_T7_sorted-W50-G50-FDR1e-10-islandfiltered-normalized.wig
Brg1-AID_CnR_MyoD-ER_4OHT_1h_K27ac_sorted-W200-G200-FDR0.001-islandfiltered-normalized.wig
Brg1-AID_CnR_MyoD-ER_4OHT_1h_K4me1_sorted-W200-G200-FDR0.001-islandfiltered-normalized.wig
Brg1-AID_CnR_MyoD-ER_4OHT_1h_T7_sorted-W50-G50-FDR1e-10-islandfiltered-normalized.wig
Brg1-AID_CnR_MyoD-ER_4OHT_4h_K27ac_sorted-W200-G200-FDR0.001-islandfiltered-normalized.wig
Brg1-AID_CnR_MyoD-ER_4OHT_4h_K4me1_sorted-W200-G200-FDR0.001-islandfiltered-normalized.wig
Brg1-AID_CnR_MyoD-ER_4OHT_4h_T7_sorted-W50-G50-FDR1e-10-islandfiltered-normalized.wig
Brg1-AID_CnR_MyoD-ER_4OHT_9h_K27ac_sorted-W200-G200-FDR0.001-islandfiltered-normalized.wig
Brg1-AID_CnR_MyoD-ER_4OHT_9h_K4me1_sorted-W200-G200-FDR0.001-islandfiltered-normalized.wig
Brg1-AID_CnR_MyoD-ER_4OHT_9h_T7_sorted-W50-G50-FDR1e-10-islandfiltered-normalized.wig
Brg1-AID_CnR_MyoD-ER_4OHT_24h_K27ac_sorted-W200-G200-FDR0.001-islandfiltered-normalized.wig
Brg1-AID_CnR_MyoD-ER_4OHT_24h_K4me1_sorted-W200-G200-FDR0.001-islandfiltered-normalized.wig
Brg1-AID_CnR_MyoD-ER_4OHT_24h_T7_sorted-W50-G50-FDR1e-10-islandfiltered-normalized.wig
Brg1-AID_CnR_MyoD-ER_4OHT_72h_K27ac_sorted-W200-G200-FDR0.001-islandfiltered-normalized.wig
Brg1-AID_CnR_MyoD-ER_4OHT_72h_K4me1_sorted-W200-G200-FDR0.001-islandfiltered-normalized.wig
Brg1-AID_CnR_MyoD-ER_4OHT_72h_T7_sorted-W50-G50-FDR1e-10-islandfiltered-normalized.wig
Mll4-AID_MyoD-ER_unt_ARID1A-W50-G50-FDR1e-10-islandfiltered-normalized.wig
Mll4-AID_MyoD-ER_unt_MLL4-W50-G50-FDR1e-10-islandfiltered-normalized.wig
Mll4-AID_MyoD-ER_unt_P300-W50-G50-FDR1e-10-islandfiltered-normalized.wig
Mll4-AID_MyoD-ER_unt_T7-W50-G50-FDR1e-10-islandfiltered-normalized.wig
Mll4-AID_MyoD-ER_4OHT_ARID1A-W50-G50-FDR1e-10-islandfiltered-normalized.wig
Mll4-AID_MyoD-ER_4OHT_MLL4-W50-G50-FDR1e-10-islandfiltered-normalized.wig
Mll4-AID_MyoD-ER_4OHT_P300-W50-G50-FDR1e-10-islandfiltered-normalized.wig
Mll4-AID_MyoD-ER_4OHT_T7-W50-G50-FDR1e-10-islandfiltered-normalized.wig
Mll4-AID_MyoD-ER_IAA_4OHT_ARID1A-W50-G50-FDR1e-10-islandfiltered-normalized.wig
Mll4-AID_MyoD-ER_IAA_4OHT_MLL4-W50-G50-FDR1e-10-islandfiltered-normalized.wig
Mll4-AID_MyoD-ER_IAA_4OHT_P300-W50-G50-FDR1e-10-islandfiltered-normalized.wig
Mll4-AID_MyoD-ER_IAA_4OHT_T7-W50-G50-FDR1e-10-islandfiltered-normalized.wig
Mll4-AID_MyoD-ER_BRM_4OHT_ARID1A-W50-G50-FDR1e-10-islandfiltered-normalized.wig
Mll4-AID_MyoD-ER_BRM_4OHT_MLL4-W50-G50-FDR1e-10-islandfiltered-normalized.wig
Mll4-AID_MyoD-ER_BRM_4OHT_P300-W50-G50-FDR1e-10-islandfiltered-normalized.wig

Mll4-AID_MyoD-ER_BRM_4OHT_T7-W50-G50-FDR1e-10-islandfiltered-normalized.wig
Mll4-AID_MyoD-ER_A485_4OHT_ARID1A-W50-G50-FDR1e-10-islandfiltered-normalized.wig
Mll4-AID_MyoD-ER_A485_4OHT_MLL4-W50-G50-FDR1e-10-islandfiltered-normalized.wig
Mll4-AID_MyoD-ER_A485_4OHT_P300-W50-G50-FDR1e-10-islandfiltered-normalized.wig
Mll4-AID_MyoD-ER_A485_4OHT_T7-W50-G50-FDR1e-10-islandfiltered-normalized.wig
Mll4_AID_MyoD_ER_unt_ARID1A_sorted-W50-G50-FDR1e-10-islandfiltered-normalized.wig
Mll4_AID_MyoD_ER_unt_MLL4_sorted-W50-G50-FDR1e-10-islandfiltered-normalized.wig
Mll4_AID_MyoD_ER_unt_T7_sorted-W50-G50-FDR1e-10-islandfiltered-normalized.wig
Mll4_AID_MyoD_ER_unt_p300_CST_sorted-W50-G50-FDR1e-10-islandfiltered-normalized.wig
Mll4_AID_MyoD_ER_4OHT_ARID1A_sorted-W50-G50-FDR1e-10-islandfiltered-normalized.wig
Mll4_AID_MyoD_ER_4OHT_MLL4_sorted-W50-G50-FDR1e-10-islandfiltered-normalized.wig
Mll4_AID_MyoD_ER_4OHT_T7_sorted-W50-G50-FDR1e-10-islandfiltered-normalized.wig
Mll4_AID_MyoD_ER_4OHT_p300_CST_sorted-W50-G50-FDR1e-10-islandfiltered-normalized.wig
Mll4_AID_MyoD_ER_p300_inh_4OHT_ARID1A_sorted-W50-G50-FDR1e-10-islandfiltered-normalized.wig
Mll4_AID_MyoD_ER_p300_inh_4OHT_MLL4_sorted-W50-G50-FDR1e-10-islandfiltered-normalized.wig
Mll4_AID_MyoD_ER_p300_inh_4OHT_T7_sorted-W50-G50-FDR1e-10-islandfiltered-normalized.wig
Mll4_AID_MyoD_ER_p300_inh_4OHT_p300_CST_sorted-W50-G50-FDR1e-10-islandfiltered-normalized.wig
Mll4_AID_MyoD_ER_p300_deg_4OHT_ARID1A_sorted-W50-G50-FDR1e-10-islandfiltered-normalized.wig
Mll4_AID_MyoD_ER_p300_deg_4OHT_MLL4_sorted-W50-G50-FDR1e-10-islandfiltered-normalized.wig
Mll4_AID_MyoD_ER_p300_deg_4OHT_T7_sorted-W50-G50-FDR1e-10-islandfiltered-normalized.wig
Mll4_AID_MyoD_ER_p300_deg_4OHT_p300_CST_sorted-W50-G50-FDR1e-10-islandfiltered-normalized.wig
Mll4-AID_MyoD-ER_unt_ATAC_S1_L001-normalized.wig
Mll4-AID_MyoD-ER_4OHT_ATAC_S2_L001-normalized.wig
Mll4-AID_MyoD-ER_BRM_4OHT_ATAC_S3_L001-normalized.wig.gz
Mll4-AID_MyoD-ER_A485_4OHT_S5_L001-normalized.wig.gz
Mll4-AID_par_Dex_ARID1A_sorted-W50-G50-FDR1e-10-islandfiltered-normalized.wig
Mll4-AID_par_Dex_GR_M20_sorted-W50-G50-FDR1e-10-islandfiltered-normalized.wig
Mll4-AID_par_Dex_KMT2D_sorted-W50-G50-FDR1e-10-islandfiltered-normalized.wig
Mll4-AID_par_Dex_p300_sorted-W50-G50-FDR0.001-islandfiltered-normalized.wig
Mll4-AID_par_dMLL4-Dex_ARID1A_sorted-W50-G50-FDR1e-10-islandfiltered-normalized.wig
Mll4-AID_par_dMLL4-Dex_GR_M20_sorted-W50-G50-FDR1e-10-islandfiltered-normalized.wig
Mll4-AID_par_dMLL4-Dex_KMT2D_sorted-W50-G50-FDR1e-10-islandfiltered-normalized.wig
Mll4-AID_par_dMLL4-Dex_p300_sorted-W50-G50-FDR0.001-islandfiltered-normalized.wig
Mll4-AID_par_BRG1i-Dex_ARID1A_sorted-W50-G50-FDR1e-10-islandfiltered-normalized.wig
Mll4-AID_par_BRG1i-Dex_GR_M20_sorted-W50-G50-FDR1e-10-islandfiltered-normalized.wig
Mll4-AID_par_BRG1i-Dex_KMT2D_sorted-W50-G50-FDR1e-10-islandfiltered-normalized.wig
Mll4-AID_par_BRG1i-Dex_p300_sorted-W50-G50-FDR0.001-islandfiltered-normalized.wig
Mll4-AID_par_p300i-Dex_ARID1A_sorted-W50-G50-FDR1e-10-islandfiltered-normalized.wig
Mll4-AID_par_p300i-Dex_GR_M20_sorted-W50-G50-FDR1e-10-islandfiltered-normalized.wig
Mll4-AID_par_p300i-Dex_KMT2D_sorted-W50-G50-FDR1e-10-islandfiltered-normalized.wig
Mll4-AID_par_p300i-Dex_p300_sorted-W50-G50-FDR0.001-islandfiltered-normalized.wig
Mll4-AID_MyoD-ER-WT_ETOH_ARID1A_R1.fastq.gz
Mll4-AID_MyoD-ER-WT_ETOH_ARID1A_R2.fastq.gz
Mll4-AID_MyoD-ER-WT_ETOH_input_R1.fastq.gz
Mll4-AID_MyoD-ER-WT_ETOH_input_R2.fastq.gz
Mll4-AID_MyoD-ER-WT_ETOH_KMT2D_R1.fastq.gz
Mll4-AID_MyoD-ER-WT_ETOH_KMT2D_R2.fastq.gz
Mll4-AID_MyoD-ER-WT_ETOH_p300_R1.fastq.gz
Mll4-AID_MyoD-ER-WT_ETOH_p300_R2.fastq.gz
Mll4-AID_MyoD-ER-WT_ETOH_T7_R1.fastq.gz
Mll4-AID_MyoD-ER-WT_ETOH_T7_R2.fastq.gz
Mll4-AID_MyoD-ER-WT_4OHT_1h_ARID1A_R1.fastq.gz
Mll4-AID_MyoD-ER-WT_4OHT_1h_ARID1A_R2.fastq.gz
Mll4-AID_MyoD-ER-WT_4OHT_1h_input_R1.fastq.gz
Mll4-AID_MyoD-ER-WT_4OHT_1h_input_R2.fastq.gz
Mll4-AID_MyoD-ER-WT_4OHT_1h_KMT2D_R1.fastq.gz
Mll4-AID_MyoD-ER-WT_4OHT_1h_KMT2D_R2.fastq.gz
Mll4-AID_MyoD-ER-WT_4OHT_1h_p300_R1.fastq.gz
Mll4-AID_MyoD-ER-WT_4OHT_1h_p300_R2.fastq.gz
Mll4-AID_MyoD-ER-WT_4OHT_1h_T7_R1.fastq.gz
Mll4-AID_MyoD-ER-WT_4OHT_1h_T7_R2.fastq.gz
Mll4-AID_MyoD-ER-KR_4OHT_1h_ARID1A_R1.fastq.gz
Mll4-AID_MyoD-ER-KR_4OHT_1h_ARID1A_R2.fastq.gz
Mll4-AID_MyoD-ER-KR_4OHT_1h_input_R1.fastq.gz
Mll4-AID_MyoD-ER-KR_4OHT_1h_input_R2.fastq.gz
Mll4-AID_MyoD-ER-KR_4OHT_1h_KMT2D_R1.fastq.gz
Mll4-AID_MyoD-ER-KR_4OHT_1h_KMT2D_R2.fastq.gz
Mll4-AID_MyoD-ER-KR_4OHT_1h_p300_R1.fastq.gz
Mll4-AID_MyoD-ER-KR_4OHT_1h_p300_R2.fastq.gz

Mll4-AID_MyoD-ER-KR_4OHT_1h_T7_R1.fastq.gz
Mll4-AID_MyoD-ER-KR_4OHT_1h_T7_R2.fastq.gz
NasRNA_Mll4-AID_MyoD-ER-WT_ETOH_rep1_S17_L002_R1_001.fastq.gz
NasRNA_Mll4-AID_MyoD-ER-WT_ETOH_rep1_S17_L002_R2_001.fastq.gz
NasRNA_Mll4-AID_MyoD-ER-WT_ETOH_rep2_S18_L002_R1_001.fastq.gz
NasRNA_Mll4-AID_MyoD-ER-WT_ETOH_rep2_S18_L002_R2_001.fastq.gz
NasRNA_Mll4-AID_MyoD-ER-WT_4OHT_1h_rep1_S19_L002_R1_001.fastq.gz
NasRNA_Mll4-AID_MyoD-ER-WT_4OHT_1h_rep1_S19_L002_R2_001.fastq.gz
NasRNA_Mll4-AID_MyoD-ER-WT_4OHT_1h_rep2_S20_L002_R1_001.fastq.gz
NasRNA_Mll4-AID_MyoD-ER-WT_4OHT_1h_rep2_S20_L002_R2_001.fastq.gz
NasRNA_Mll4-AID_MyoD-ER-WT_4OHT_4h_rep1_S21_L002_R1_001.fastq.gz
NasRNA_Mll4-AID_MyoD-ER-WT_4OHT_4h_rep1_S21_L002_R2_001.fastq.gz
NasRNA_Mll4-AID_MyoD-ER-WT_4OHT_4h_rep2_S22_L002_R1_001.fastq.gz
NasRNA_Mll4-AID_MyoD-ER-WT_4OHT_4h_rep2_S22_L002_R2_001.fastq.gz
NasRNA_Mll4-AID_MyoD-ER-WT_4OHT_9h_rep1_S23_L002_R1_001.fastq.gz
NasRNA_Mll4-AID_MyoD-ER-WT_4OHT_9h_rep1_S23_L002_R2_001.fastq.gz
NasRNA_Mll4-AID_MyoD-ER-WT_4OHT_9h_rep2_S24_L002_R1_001.fastq.gz
NasRNA_Mll4-AID_MyoD-ER-WT_4OHT_9h_rep2_S24_L002_R2_001.fastq.gz
NasRNA_Mll4-AID_MyoD-ER-WT_4OHT_24h_rep1_S25_L002_R1_001.fastq.gz
NasRNA_Mll4-AID_MyoD-ER-WT_4OHT_24h_rep1_S25_L002_R2_001.fastq.gz
NasRNA_Mll4-AID_MyoD-ER-WT_4OHT_24h_rep2_S26_L002_R1_001.fastq.gz
NasRNA_Mll4-AID_MyoD-ER-WT_4OHT_24h_rep2_S26_L002_R2_001.fastq.gz
Mll4-AID_MyoD-ER-WT_ETOH_ARID1A_sorted-W50-G50-FDR1e-10-islandfiltered-normalized.wig
Mll4-AID_MyoD-ER-WT_ETOH_KMT2D_sorted-W50-G50-FDR1e-10-islandfiltered-normalized.wig
Mll4-AID_MyoD-ER-WT_ETOH_p300_sorted-W50-G50-FDR0.001-islandfiltered-normalized.wig
Mll4-AID_MyoD-ER-WT_ETOH_T7_sorted-W50-G50-FDR1e-50-islandfiltered-normalized.wig
Mll4-AID_MyoD-ER-WT_4OHT_1h_ARID1A_sorted-W50-G50-FDR1e-10-islandfiltered-normalized.wig
Mll4-AID_MyoD-ER-WT_4OHT_1h_KMT2D_sorted-W50-G50-FDR1e-10-islandfiltered-normalized.wig
Mll4-AID_MyoD-ER-WT_4OHT_1h_p300_sorted-W50-G50-FDR0.001-islandfiltered-normalized.wig
Mll4-AID_MyoD-ER-WT_4OHT_1h_T7_sorted-W50-G50-FDR1e-50-islandfiltered-normalized.wig
Mll4-AID_MyoD-ER-KR_4OHT_1h_ARID1A_sorted-W50-G50-FDR1e-10-islandfiltered-normalized.wig
Mll4-AID_MyoD-ER-KR_4OHT_1h_KMT2D_sorted-W50-G50-FDR1e-10-islandfiltered-normalized.wig
Mll4-AID_MyoD-ER-KR_4OHT_1h_p300_sorted-W50-G50-FDR0.001-islandfiltered-normalized.wig
Mll4-AID_MyoD-ER-KR_4OHT_1h_T7_sorted-W50-G50-FDR1e-50-islandfiltered-normalized.wig
NasRNA_ETOH_rep1_STAR_mm10_STAR_on_Genebody.txt
NasRNA_ETOH_rep2_STAR_mm10_STAR_on_Genebody.txt
NasRNA_1h_rep1_STAR_mm10_STAR_on_Genebody.txt
NasRNA_1h_rep2_STAR_mm10_STAR_on_Genebody.txt
NasRNA_4h_rep1_STAR_mm10_STAR_on_Genebody.txt
NasRNA_4h_rep2_STAR_mm10_STAR_on_Genebody.txt
NasRNA_9h_rep1_STAR_mm10_STAR_on_Genebody.txt
NasRNA_9h_rep2_STAR_mm10_STAR_on_Genebody.txt
NasRNA_24h_rep1_STAR_mm10_STAR_on_Genebody.txt
NasRNA_24h_rep2_STAR_mm10_STAR_on_Genebody.txt

Genome browser session (e.g. UCSC)

No longer applicable

Methodology

Replicates

Most CUT&RUN and ChIP–seq experiments were performed in duplicate or triplicate, with representative data from a single experiment shown. ATAC-seq and nascent RNA-seq data are from single experiment.

Sequencing depth

Each library contains at least 20 million reads.

Antibodies

Anti-T7 tag (D9E1X; 13246S; 1 µl per C&R), anti-ARID1A/BAF250A (D2A8U; 12354, 1 µl per C&R), and anti-p300 (D8Z4E; 86377, 1 µl per C&R, 15 µl per ChIP) were obtained from Cell Signaling Technology.
Anti-H3K27ac (ab4729; 0.5 µg per C&R) was obtained from Abcam.
Anti-H3K4me1 (13-0040; 0.5 µg per C&R), and rabbit control IgG (13-0042K) were obtained from EpiCypher.
Anti-MyoD (5.8A; sc-32758, 4 µg per ChIP) and anti-GR (M-20; sc-100041, 4 µg per ChIP) were obtained from Santa Cruz Biotechnology.
Anti-KMT2D (#3; 2 µg per C&R, 8 µg per ChIP) was home-made.

Peak calling parameters

For the identification of ChIP-seq enriched regions, SICER (version 2) was used. For non-histone factors, a window size of 50 bp, gap size of 50 bp, and a false discovery rate of 1E-10 were used. For H3K4me1 and H3K27ac, a window size of 200 bp, gap size of 200 bp, and a false discovery rate of 1E-3 were used. The effective genome fraction of 0.8 was applied to all ChIP-Seq data.

Data quality

For CUT&RUN of histone modifications (H3K4me1 and H3K27ac), peaks with the false discovery rate (FDR) < 1E-3 were included in the data analysis. For ChIP-seq of non-histone factors, peaks with FDR < 1E-10 were included in the data analysis.

Software	Software for CUT&RUN, ChIP-seq, ATAC-seq and RNA-seq data processing and analysis include bowtie2 (v2.3.4.1), STAR(v2.7.11b) and SICER algorithm (v2).
