# Supplementary material for: Chromatin modifiers KMT2D, cBAF, and p300 cooperatively facilitate de novo binding of transcription factors on enhancers
Source: Nat Commun. 2026 Sep 15;17:10816. doi: 10.1038/s41467-026-77757-w (PMC13669397; doi:10.1038/s41467-026-77757-w)
Supplement: Supplementary file 3 [file 41467_2026_77757_MOESM3_ESM.pdf]

nature portfolio

Peer Review File

Chromatin modifiers KMT2D, cBAF, and p300 cooperatively facilitate *de novo* binding of transcription factors on enhancers

Corresponding Author: Dr Kai Ge

This file contains all reviewer reports in order by version, followed by all author rebuttals in order by version.

Version 0:

Reviewer comments:

Reviewer #1

(Remarks to the Author)
SUMMARY:
In the manuscript, "Chromatin modifiers KMT2D, BAF, and p300 are required for de novo binding of transcription factors on enhancers," Van et al. use recent advances in rapid protein induction and depletion methods to challenge the hierarchical model of de novo enhancer activation. They use a trans-differentiation model in which the pioneer TF MyoD is rapidly translocated to the nucleus, which is sufficient to reprogram adipocytes to myocytes. After defining MyoD+ enhancers, the authors compare early binding at enhancers upon MyoD induction compared to induction after perturbation of chromatin regulators to measure the impact of each on the recruitment/stability of the others. The findings of this study largely identify a cooperative role of these factors, which is somewhat more prominently revealed at de novo MyoD sites that lacked pre-binding of the chromatin regulators than at sites where the chromatin regulators were pre-bound. While substantial evidence of cooperative roles have previously been published the present study uses acute induction of MyoD, follows changes temporally and systematically measures the relevant regulators, thereby being notably rigorous and comprehensive. The findings thereby contribute to understanding the biology of early enhancer activation and provide mechanistic insight into the interplay of a lineage regulating TF with chromatin biology that underlies development and, when mutated, constitutes the basis of numerous pathologies.
Strengths: The experiments are well done and the quality of the data is good. The rapid TF induction, temporal monitoring and systemtic evaluation and manipulation of multiple TFs and chromatin regulators provides substantial and robust data. This provides novel insights into the dynamics of enhancer assembly and maintenance.
Weaknesses: The focus on "de novo" binding and the suggestion of novelty in identifying cooperativity between chromatin factors seems overdone since cooperative acitivty exists at both established and de novo sites, it is simply somewhat more prominent at the de novo sites, and since cooperativity in TF – chromatin regulator interactions have been previously demonstrated. Rather the impact here is derivied from the systematic and robust approach, which brings confidence and therefore real value.
With respect to revisions, I find that the experiments are well done and are comprehensive. I don't feel that the pursuit of additional experiments would add substantially to the conclusions.

Suggested revisions
I suggest that the title be modified to "Chromatin modifiers KMT2D, BAF, and p300 are preferentially required for de novo binding of transcription factors on enhancers,"
The manuscript over does the claim of novelty regarding a cooperative model for interaction of chromatin factors. In the introduction the manuscript states "Loss of KMT2D disrupts p300 recruitment and impairs de novo enhancer activation [2, 3, 12], suggesting that p300 functions downstream of KMT2D." The conclusion that p300 functions downstream of KMT2D seems misleading as the loss of p300 recruitment would be expected in both hierarchical and cooperative models. The introduction next states "KMT2D also physically associates with BAF, and loss of BAF's ATPase subunit BRG1 blocks activation of C/EBPβ-bound adipogenic enhancers. BAF and KMT2D reciprocally regulate each other's binding on enhancers [8]. These findings suggest a stepwise model of enhancer activation." This again seems inaccurate as there is nothing about these findings that would seem to distinguish between hierarchical and cooperative models.

Next, near the end of the manuscript is stated "we demonstrate that, in addition to the previously suggested model of

sequential recruitment of TFs, KMT2D, BAF, and p300 (Fig. 7A), TFs and these chromatin modifiers also exhibit interdependent binding relationships on enhancers (Fig. 7B).” This seems to claim that novelty in finding cooperativity. Prior publications have demonstrated complexity in models of binding and have also shown that events can vary by TF and by individual binding site (some examples include PMIDs 31639474, 31350899, 41372234, 36536103. The data in the present manuscript has aspects that are hierarchical and aspects that are cooperative, with different magnitude of contribution from different factors. Because of the systematic approach of analyzing multiple factors at multiple time points with two different TFs, the mansuscripts findings have clear value. However, I would encourage the authors to synthesize the principles that emerge and back off on the claim of novelty of finding cooperative aspects and back off on the heavy focus on “de novo” since the differences occur on a spectrum rather than absolute differences.

Reviewer #2

(Remarks to the Author)
I co-reviewed this manuscript with one of the reviewers who provided the listed reports. This is part of the Nature Communications initiative to facilitate training in peer review and to provide appropriate recognition for Early Career Researchers who co-review manuscripts.

Reviewer #3

(Remarks to the Author)
This is a very technically sophisticated study, in which the authors address whether the chromatin modifiers KMT2D, BAF, and p300 are necessary to maintain the binding of either one another to genomic regulatory elements and/or the binding of lineage-determining or signal-transducing transcription factors to these regulatory elements. The authors have generated a preadipocyte cell line which both lacks Kmt2c and contains a degron-tagged Kmt2d; which allows them to transiently remove all Kmt2c/d activity. The authors have expressed in this cell line an epitope-tagged version of MyoD-ERt2, which translocates into the nucleus following 4-hydroxytamoxifen exposure, and have examined whether chromatin occupancy of MyoD-ERt2 is altered by either removal of Kmt2c/d or by inhibition of either BAF or p300. This novel experimental set up has allowed the authors to identify MyoD-bound regulatory elements which are preoccupied by KMT2D, BAF, and p300 prior to MyoD binding, versus binding sites that recruit these three chromatin modifiers only after MyoD binding; and have determined whether the activity of these chromatin modifiers is necessary to maintain the binding of either one another or MyoD to these sites. The authors also extend this same type of analysis to the glucocorticoid receptor (GR). Most importantly, the authors document that the majority of MyoD and/or GR binding sites require all of these chromatin modifiers to allow/maintain de novo binding of either MyoD-ERt2 or GR to regulatory elements. This is an important finding, as it indicates that both lineage-determining TFs, such as MyoD, and signal-transducing TFs (such as GR) require the recruitment of various chromatin modifiers (particularly BAF and p300) to allow de novo binding of these TFs on a majority of regulatory elements. I think that this analysis merits publication in Nature Communications. That being said, I believe that the addition of the following analyses would further improve this excellent study:

1. The authors display a time course of binding sites that MyoD occupies from 1-72 hours, along with GO analysis of these binding sites in Figure 1. The authors should evaluate the enrichment of TF binding motifs at MyoD-bound regulatory regions for each of these time points. As the latter time points are enriched in skeletal muscle differentiation genes, do they display evidence of additional TF binding sites such as MEF2 along with MyoD?

2. The authors make the very interesting observation that MyoD is recruited at 1 hour to many sites that are already bound by KMT2D, BAF, and p300. If I understand the p-values of the Homer analysis correctly, the majority of these binding sites lack a consensus MyoD binding site (as evidenced by a p value of 1E -47); but these same sites display high MyoD occupancy (based on RPKM). In contrast the majority of MyoD binding sites that display de novo recruitment of KMT2D, BAF, and p300 indeed contain a MyoD binding site (as evidenced by a p value of 1E-259), but these regulatory regions display a lower MyoD occupancy (based on RPKM). I recommend that the authors determine whether the MyoD interaction with these two distinct binding sites requires an intact DNA binding domain in MyoD. The authors could investigate whether mutation of the MyoD basic domain (which have lost the ability to bind DNA) are differentially recruited to these two distinct binding sites.

3. MyoD contains a strong N-terminal activation domain (which contains an intrinsically disordered region), that is included in their MyoD-ERt2 construct. I recommend that the authors determine whether the MyoD N-terminus activation domain is necessary for either MyoD binding or recruitment of chromatin modifiers to the two classes of MyoD binding sites discussed above.

The above mutational analysis of the MyoD domains necessary for either direct DNA interaction or transcriptional activation, would nicely complement the loss of function studies (of the chromatin modifiers) that are recruited by MyoD, which the authors have so carefully documented.

Reviewer #4

(Remarks to the Author)
In this manuscript, the authors used a system of inducible MyoD translocation into the nuclei of mouse pre-adipocytes to investigate the temporal/functional relationship between chromatin recruitment of MyoD, the methyltransferase KMT2D,

components of the SWI/SNF chromatin remodeling complex and the H3K27 acetyltransferase p300, genome-wide. Cut&Run/ChIP-seq and ATACseq analysis was used to monitor chromatin recruitment of ectopically expressed MyoD and accessibility upon acute depletion of KMT2D or short-term inhibition of BAF or p300 enzymatic activity. By these combinatorial experimental manipulations, the authors identified different subsets of MyoD-bound enhancers, and decided to focus on specific subsets of enhancers in which they observed reduced binding of MyoD as well as of KMT2D, BAF, and p300, indicating a cooperative recruitment among chromatin modifiers. Similar interdependent relationships are observed between the signal-dependent TF Glucocorticoid Receptor and KMT2D, BAF, and p300.

While the results of this study might be of potential interest in term of providing mechanistic insights into the functional relationship between transcription factors and chromatin modifiers, there are a number of concerns and issues that I recommend the authors to address – see below.

1) The authors missed considering their results within the context of the existing literature and state of the art of MyoD binding to enhancers and relative regulation of chromatin modifiers, that have been previously published – e.g. works from Blum and colleagues (2012) showing the relationship between MyoD binding to enhancers and monomethylation of H3K4 (H3K4me1) and acetylation of H3K27 (H3K27ac) in a more physiological context (muscle stem cells); works from Cao et al. (2010) showing that MyoD is constitutively pre-bound to chromatin at thousands of distal potential enhancers in myoblasts; works from Serra et al. (2007) that anticipated a functional interdependence between MyoD chromatin binding, acetyltransferases and SWI/SNF; recent works from Dall'Agnese et al. (2019) and Wang et al. (2022) reporting on MyoD genome-wide chromatin binding within the context of 3D genome conformation. These, and possibly other related publications should be cited and discussed them to determine the relative novelty and potential conflicts with previous literature of the findings reported in this manuscript.

2) One important missing piece of evidence is the lack of transcriptional outcome of the changes observed at MyoD bound enhancers. In Fig 1-S1 G the authors show a GO analysis, but it is unclear to what it refers, as there are no RNAseq data of reference and relative analysis that could link enhancers to coding genes.

3) Also, GO analysis shown in Fig 1-S1 G indicates biological processes that are typically repressed during skeletal myogenesis (especially in clusters B and C) and that predict MyoD-bound enhancers that mediated repression, as recently shown in Nicoletti et al. (2025). The authors should consider analyzing the functional interdependence of MyoD, KMT2D, BAF, and p300 within the context of gene repression.

4) Fig 1-S1 G shows an intersection of MyoD bound enhancers and histone modifications - H3K4me1 and H3K27ac. However, it is unclear what data the refer to. Cut&Run datasets of H3K4me1 and H3K27ac are not shown. For proper evaluation of the relationship between MyoD and dynamic changes of H3K4me1 and H3K27ac at these enhancers, it is necessary that the authors perform H3K4me1 and H3K27ac Cut&Run analysis before and after MyoD induction. Again, this should be associated with RNAseq.

5) It is unclear why the authors decided to perform their experiments in pre-adipocytes. No explaination is provided in the text. Moreover, how was immortalization of pre-adipocytes obtained? This needs to be described, as it could be an operation that might alter MyoD function and chromatin binding.

6) The author's conclusions are based on the assumption that ARID1A and BRG1 are representative of muscle SWI/SNF complexes, which is instead highly heterogeneous. Different BAF complexes are distributed genome-wide, with some of them being devoid of Brg1 (replaced by the alternative catalytic sub-unit Brm) and ARID1A, as also previously reported in Albini et al (2015) and Sharma et al. (2021). It is recommended that the authors monitor the chromatin binding of additional SWI/SNF components (e.g. BAF47, Brm and BAF60c).

Version 1:

Reviewer comments:

Reviewer #1

(Remarks to the Author)
The authors have thoughtfully and fully addressed each of my critiques.

Reviewer #2

(Remarks to the Author)
I co-reviewed this manuscript with one of the reviewers who provided the listed reports. This is part of the Nature Communications initiative to facilitate training in peer review and to provide appropriate recognition for Early Career Researchers who co-review manuscripts.

Reviewer #3

(Remarks to the Author)
This revised manuscript now includes new data and a markedly expanded discussion; that have significantly improved this work. I appreciate that the authors made the effort to add nascent RNA transcriptome analysis to this work and also evaluated the ability of a MyoD mutant that lacks acetylation sites; with consequent interesting new insights! In addition, the more encyclopedic nature of the discussion is a significant improvement; as this work can now be better appreciated in the context of a large body of literature on TF-chromatin modifier interactions/synergy. In summary, this revised manuscript merits publication in Nature Communications in its present form. I congratulate the authors on a nice piece of very rigorous

work.

Reviewer #4

(Remarks to the Author)
In the revised version of the manuscript the authors have addressed the most important issues raised by the reviewers and the manuscript is definitely improved and suitable for publication, in the opinion of this reviewer.

Open Access This Peer Review File is licensed under a Creative Commons Attribution 4.0 International License, which permits use, sharing, adaptation, distribution and reproduction in any medium or format, as long as you give appropriate credit to the original author(s) and the source, provide a link to the Creative Commons license, and indicate if changes were made.
In cases where reviewers are anonymous, credit should be given to 'Anonymous Referee' and the source.
The images or other third party material in this Peer Review File are included in the article's Creative Commons license, unless indicated otherwise in a credit line to the material. If material is not included in the article's Creative Commons license and your intended use is not permitted by statutory regulation or exceeds the permitted use, you will need to obtain permission directly from the copyright holder.
To view a copy of this license, visit https://creativecommons.org/licenses/by/4.0/

REVIEWER COMMENTS

Reviewer #1 (Remarks to the Author):

SUMMARY:

In the manuscript, “Chromatin modifiers KMT2D, BAF, and p300 are required for de novo binding of transcription factors on enhancers,” Van et al. use recent advances in rapid protein induction and depletion methods to challenge the hierarchical model of de novo enhancer activation. They use a trans-differentiation model in which the pioneer TF MyoD is rapidly translocated to the nucleus, which is sufficient to reprogram adipocytes to myocytes. After defining MyoD+ enhancers, the authors compare early binding at enhancers upon MyoD induction compared to induction after perturbation of chromatin regulators to measure the impact of each on the recruitment/stability of the others. The findings of this study largely identify a cooperative role of these factors, which is somewhat more prominently revealed at de novo MyoD sites that lacked pre-binding of the chromatin regulators than at sites where the chromatin regulators were pre-bound. While substantial evidence of cooperative roles have previously been published the present study uses acute induction of MyoD, follows changes temporally and systematically measures the relevant regulators, thereby being notably rigorous and comprehensive. The findings thereby contribute to understanding the biology of early enhancer activation and provide mechanistic insight into the interplay of a lineage regulating TF with chromatin biology that underlies development and, when mutated, constitutes the basis of numerous pathologies.

Strengths: The experiments are well done and the quality of the data is good. The rapid TF induction, temporal monitoring and systemtic evaluation and manipulation of multiple TFs and chromatin regulators provides substantial and robust data. This provides novel insights into the dynamics of enhancer assembly and maintenance.

Weaknesses: The focus on “de novo” binding and the suggestion of novelty in identifying cooperativity between chromatin factors seems overdone since cooperative acitivty exists at both established and de novo sites, it is simply somewhat more prominent at the de novo sites, and since cooperativity in TF – chromatin regulator interactions have been previously demonstrated. Rather the impact here is derivied from the systematic and robust approach, which brings confidence and therefore real value.

With respect to revisions, I find that the experiments are well done and are comprehensive. I don’t feel that the pursuit of additional experiments would add substantially to the conclusions.

Suggested revisions

I suggest that the title be modified to “Chromatin modifiers KMT2D, BAF, and p300 are preferentially required for de novo binding of transcription factors on enhancers,”

The manuscript over does the claim of novelty regarding a cooperative model for interaction of chromatin factors. In the introduction the manuscript states “Loss of KMT2D disrupts p300 recruitment and impairs de novo enhancer activation [2, 3, 12], suggesting that p300 functions downstream of KMT2D.” The conclusion that p300 functions downstream of KMT2D seems misleading as the loss of p300 recruitment would be expected in both hierarchical and cooperative models. The introduction next states “KMT2D also physically associates with BAF, and loss of BAF’s ATPase subunit BRG1 blocks activation of C/EBPβ-bound adipogenic enhancers. BAF and KMT2D reciprocally regulate each other’s binding on enhancers [8]. These findings suggest a stepwise model of enhancer activation.” This again seems inaccurate as there is nothing about these findings that would seem to distinguish between hierarchical and cooperative models.

Next, near the end of the manuscript is stated “we demonstrate that, in addition to the previously suggested model of sequential recruitment of TFs, KMT2D, BAF, and p300 (Fig. 7A), TFs and these chromatin modifiers also exhibit interdependent binding relationships on enhancers (Fig. 7B).” This seems to claim that novelty in finding cooperativity. Prior publications have demonstrated complexity in models of binding and have also shown that events can vary by TF and by individual binding site (some examples include PMIDs 31639474, 31350899, 41372234, 36536103. The data in the present manuscript has aspects that are hierarchical and aspects that are cooperative, with different magnitude of contribution from different factors. Because of the systematic approach of analyzing multiple factors at multiple time points with two different TFs, the mansuscripts findings have clear value. However, I would encourage the authors to synthesize the principles that emerge and back off on the claim of novelty of finding cooperative aspects and back off on the heavy focus on “de novo” since the differences occur on a spectrum rather than absolute differences.

We thank the reviewer for thoughtful and constructive comments. We appreciate the recognition of the rigor and comprehensiveness of our experimental approach. We have carefully considered all suggestions and revised the manuscript accordingly.

Reviewer's comment: “I suggest that the title be modified to “Chromatin modifiers KMT2D, BAF, and p300 are preferentially required for de novo binding of transcription factors on enhancers”

Response: We appreciate the reviewer’s thoughtful suggestion. We agree with the underlying point that the requirement for these chromatin modifiers exists on a spectrum and is indeed more prominent on *de novo* sites. To address this, we have revised our title. However, we felt that adding the word "preferentially" might inadvertently downplay the important roles these modifiers also play at pre-established sites. Instead, we have changed the title to "Chromatin modifiers KMT2D, cBAF, and p300 cooperatively facilitate *de novo* binding of transcription factors on enhancers." By shifting the phrasing from "are required for" to "cooperatively facilitate," we remove the implication of a strict, absolute binary. We believe this new title better reflects the cooperative spectrum. Additionally, as detailed in our subsequent responses, we have thoroughly revised the Introduction and Discussion to highlight this spectrum and tone down any claims of absolute exclusivity.

Reviewer's comment: “The manuscript over does the claim of novelty regarding a cooperative model for interaction of chromatin factors. In the introduction the manuscript states “Loss of KMT2D disrupts p300 recruitment and impairs de novo enhancer activation [2, 3, 12], suggesting that p300 functions downstream of KMT2D.” The conclusion that p300 functions downstream of KMT2D seems misleading as the loss of p300 recruitment would be expected in both hierarchical and cooperative models. ... These findings suggest a stepwise model of enhancer activation.” This again seems inaccurate as there is nothing about these findings that would seem to distinguish between hierarchical and cooperative models.”

Response: We thank the reviewer for this important point. We agree that our previous framing overstated the distinction between hierarchical and cooperative models, and that previous observations are equally consistent with both. We have revised the Introduction to address this point. First, we replaced "suggesting that p300 functions downstream of KMT2D" with "supporting a functional relationship between KMT2D and p300". Second, we replaced the sentence "These findings suggest a stepwise model of enhancer activation: (1) LDTFs bind to enhancer regions; (2) BAF and KMT2D are recruited; and (3) p300 is recruited to promote enhancer activation" with "Collectively, these findings have been interpreted as supporting a stepwise model of enhancer activation in which LDTFs bind to enhancer regions, followed by recruitment of cBAF and KMT2D, and subsequently p300 to promote enhancer activation. However, observed interdependencies among these factors are also consistent with a cooperative model of enhancer assembly".

Reviewer's comment: “…This seems to claim that novelty in finding cooperativity. Prior publications have demonstrated complexity in models of binding and have also shown that events can vary by TF and by individual binding site (some examples include PMIDs 31639474, 31350899, 41372234, 36536103…”

Response: We thank the reviewer for raising this point and for providing the relevant references. We agree that our initial manuscript did not clearly explain how our data fit with the existing literature that has shown the complexity and cooperativity of TF-chromatin factor interactions. To address this, we removed claims of novelty, instead emphasizing how our temporal data supports existing cooperative models. We revised the Discussion introduction to frame TF-modifier interactions as a spectrum across enhancers, rather than a strict hierarchy. Additionally, we added a new section, “Hierarchical and cooperative mechanisms shaping enhancer establishment,” to synthesize these principles and contextualize our findings within the recommended literature.

Reviewer #2 (Remarks to the Author):

I co-reviewed this manuscript with one of the reviewers who provided the listed reports. This is part of the Nature Communications initiative to facilitate training in peer review and to provide appropriate recognition for Early Career Researchers who co-review manuscripts.

Reviewer #3 (Remarks to the Author):

This is a very technically sophisticated study, in which the authors address whether the chromatin modifiers KMT2D, BAF, and p300 are necessary to maintain the binding of either one another to genomic regulatory elements and/or the binding of lineage-determining or signal-transducing transcription factors to these regulatory elements. The authors have generated a preadipocyte cell line which both lacks Kmt2c and contains a degron-tagged Kmt2d; which allows them to transiently remove all Kmt2c/d activity. The authors have expressed in this cell line an epitope-tagged version of MyoD-ERt2, which translocates into the nucleus following 4-hydroxytamoxifen exposure, and have examined whether chromatin occupancy of MyoD-ERt2 is altered by either removal of Kmt2c/d or by inhibition of either BAF or p300. This novel experimental set up has allowed the authors to identify MyoD-bound regulatory elements which are preoccupied by KMT2D, BAF, and p300 prior to MyoD binding, versus binding sites that recruit these three chromatin modifiers only after MyoD binding; and have determined whether the activity of these chromatin modifiers is necessary to maintain the binding of either one another or MyoD to these sites. The authors also extend this same type of analysis to the glucocorticoid receptor (GR). Most importantly, the authors document that the majority of MyoD and/or GR binding sites require all of these chromatin modifiers to allow/maintain de novo binding of either MyoD-ERt2 or GR to regulatory elements. This is an important finding, as it indicates that both lineage-determining TFs, such as MyoD, and signal-transducing TFs (such as GR) require the recruitment of various chromatin modifiers (particularly BAF and p300) to allow de novo binding of these TFs on a majority of regulatory elements. I think that this analysis merits publication in Nature Communications. That being said, I believe that the addition of the following analyses would further improve this excellent study:

1. The authors display a time course of binding sites that MyoD occupies from 1-72 hours, along with GO analysis of these binding sites in Figure 1. The authors should evaluate the enrichment of TF binding motifs at MyoD-bound regulatory regions for each of these time points. As the latter time points are enriched in skeletal muscle differentiation genes, do they display evidence of additional TF binding sites such as MEF2 along with MyoD?

We thank the reviewer for the helpful suggestion. The time-course clustering of MyoD-bound regions was originally presented in Supplementary Fig. 1. Given its central relevance to the manuscript and the reviewer's interest in this analysis, we have now moved it to the main Fig. 1.

In response to the reviewer's suggestion, we performed motif analysis for each cluster of MyoD-bound regulatory regions. The canonical MyoD motif is highly enriched across all clusters. Notably, later clusters (D-F), which are associated with muscle differentiation genes, exhibit

additional significant enrichment of MEF2 motifs. These results are consistent with the established cooperation between MyoD and MEF2 during myogenesis and further support the temporal transition in regulatory programs revealed by our clustering analysis. The motif analysis is now included in the revised Supplementary Fig. 1e.

2. The authors make the very interesting observation that MyoD is recruited at 1 hour to many sites that are already bound by KMT2D, BAF, and p300. If I understand the p-values of the Homer analysis correctly, the majority of these binding sites lack a consensus MyoD binding site (as evidenced by a p value of 1E -47); but these same sites display high MyoD occupancy (based on RPKM). In contrast the majority of MyoD binding sites that display de novo recruitment of KMT2D, BAF, and p300 indeed contain a MyoD binding site (as evidenced by a p value of 1E-259), but these regulatory regions display a lower MyoD occupancy (based on RPKM). I recommend that the authors determine whether the MyoD interaction with these two distinct binding sites requires an intact DNA binding domain in MyoD. The authors could investigate whether mutation of the MyoD basic domain (which have lost the ability to bind DNA) are differentially recruited to these two distinct binding sites.

We thank the reviewer for this insightful comment and the suggestion to test whether recruitment of MyoD to the two groups of binding sites (BAF/KMT2D/p300 prebound vs. *de novo*) depends on an intact MyoD DNA-binding domain (DBD).

Regarding the p-values from our motif analysis, we would like to clarify that these values represent enrichment relative to background sequences. A p-value of 1E-47 at prebound sites still represents a high statistical enrichment of the canonical MyoD E-box motif. Therefore, it does not indicate that the majority of these sites lack a consensus motif. Rather, it confirms that the E-box is a central feature of both binding classes. The stronger motif enrichment at *de novo* sites (p = 1E-259) suggests that while MyoD recruitment at *de novo* sites is more strongly associated with canonical E-box sequence recognition, recruitment at prebound sites is likely further stabilized by interactions with the pre-existing BAF/KMT2D/p300.

Because the E-box motif is highly enriched in both contexts, we anticipate that a mutation of the MyoD DBD would globally reduce MyoD occupancy across all sites rather than selectively affecting *de novo* sites. We believe the most definitive test would be genome-wide mutation of E-box elements. However, given the large number of sites involved, this approach is not technically feasible at present and is beyond the scope of this study.

3. MyoD contains a strong N-terminal activation domain (which contains an intrinsically disordered region), that is included in their MyoD-ERt2 construct. I recommend that the authors determine whether the MyoD N-terminus activation domain is necessary for either MyoD binding or recruitment of chromatin modifiers to the two classes of MyoD binding sites discussed above.

The above mutational analysis of the MyoD domains necessary for either direct DNA interaction or transcriptional activation, would nicely complement the loss of function studies (of the

chromatin modifiers) that are recruited by MyoD, which the authors have so carefully documented.

We thank the reviewer for this valuable suggestion and agree that mutational analyses of MyoD domains would nicely complement our loss-of-function studies of chromatin modifiers. However, given that the primary focus of our manuscript is the relationship between MyoD and chromatin modifiers, we propose a more directly relevant approach: mutating the lysine residues (K99, K102, K104) within MyoD that are acetylated by CBP/p300 and PCAF [1,2].

We introduced the MyoD-K/R mutations into our MyoD-ER system and performed differentiation assays and ChIP-Seq analysis. Despite comparable expression levels and nuclear translocation, non-acetylatable MyoD-K/R mutant was less efficient than MyoD-WT in inducing myogenic marker genes and in promoting myogenesis. On enhancers, K/R mutations did not impair MyoD binding. However, loss of these acetylation sites significantly reduced the recruitment and/or maintenance of KMT2D, cBAF, and p300 especially on *de novo* enhancer sites. These new results, now included in Supplementary Fig. 6, show that disruption of MyoD acetylation selectively impairs genomic binding of chromatin modifiers without altering MyoD nuclear translocation or chromatin occupancy. Thus, these experiments provide mechanistic insight into the acetylation-dependent relationship between MyoD and p300, as well as KMT2D and cBAF, and substantially strengthen and complement our loss-of-function studies of the chromatin modifiers.

Reviewer #4 (Remarks to the Author):

In this manuscript, the authors used a system of inducible MyoD translocation into the nuclei of mouse pre-adipocytes to investigate the temporal/functional relationship between chromatin recruitment of MyoD, the methyltransferase KMT2D, components of the SWI/SNF chromatin remodeling complex and the H3K27 acetyltransferase p300, genome-wide. Cut&Run/ChIP-seq and ATACseq analysis was used to monitor chromatin recruitment of ectopically expressed MyoD and accessibility upon acute depletion of KMT2D or short-term inhibition of BAF or p300 enzymatic activity. By these combinatorial experimental manipulations, the authors identified different subsets of MyoD-bound enhancers, and decided to focus on specific subsets of enhancers in which they observed reduced binding of MyoD as well as of KMT2D, BAF, and p300, indicating a cooperative recruitment among chromatin modifiers. Similar interdependent relationships are observed between the signal-dependent TF Glucocorticoid Receptor and KMT2D, BAF, and p300.

While the results of this study might be of potential interest in term of providing mechanistic insights into the functional relationship between transcription factors and chromatin modifiers, there are a number of concerns and issues that I recommend the authors to address – see below.

1) The authors missed considering their results within the context of the existing literature and state of the art of MyoD binding to enhancers and relative regulation of chromatin modifiers, that

have been previously published – e.g. works from Blum and colleagues (2012) showing the relationship between MyoD binding to enhancers and monomethylation of H3K4 (H3K4me1) and acetylation of H3K27 (H3K27ac) in a more physiological context (muscle stem cells); works from Cao et al. (2010) showing that MyoD is constitutively pre-bound to chromatin at thousands of distal potential enhancers in myoblasts; works from Serra et al. (2007) that anticipated a functional interdependence between MyoD chromatin binding, acetyltransferases and SWI/SNF; recent works from Dall'Agnese et al. (2019) and Wang et al. (2022) reporting on MyoD genome-wide chromatin binding within the context of 3D genome conformation. These, and possibly other related publications should be cited and discussed them to determine the relative novelty and potential conflicts with previous literature of the findings reported in this manuscript.

We thank the reviewer for suggestions. We have revised the Discussion to include a new section, "MyoD-dependent enhancer assembly" which connects our data to the previous studies that the reviewer pointed out.

2) One important missing piece of evidence is the lack of transcriptional outcome of the changes observed at MyoD bound enhancers. In Fig 1-S1 G the authors show a GO analysis, but it is unclear to what it refers, as there are no RNAseq data of reference and relative analysis that could link enhancers to coding genes.

We thank the reviewer for pointing out the lack of clarity regarding the GO analysis in the Fig. 1-S1G (now revised Supplementary Fig. 1D). This was done using the Genomic Regions Enrichment of Annotations Tool (GREAT) [3], which assigns biological functions to non-coding genomic regions by associating them with putative target genes based on genomic proximity and curated regulatory domains. To improve transparency, we have added a subsection, "GREAT GO analysis," to the Methods of the revised manuscript.

To link MyoD-bound enhancers to transcriptional outcomes, we performed nascent RNA-seq following MyoD nuclear translocation (0, 1, 4, 9, and 24 h). These data revealed dynamic transcriptional changes in genes associated with MyoD-bound enhancers, which were classified into 5 distinct groups: rapidly, gradually, and late-upregulated genes (G1-G3), and gradually or moderately repressed genes (G4-G5). These data are now included in the revised Fig. 1 and Supplementary Fig 2. In response to the reviewer's comments and given the central importance of these results, we have moved the core data of previous Supplementary Fig. 1 to the main Fig. 1.

3) Also, GO analysis shown in Fig 1-S1 G indicates biological processes that are typically repressed during skeletal myogenesis (especially in clusters B and C) and that predict MyoD-bound enhancers that mediated repression, as recently shown in Nicoletti et al. (2025). The authors should consider analyzing the functional interdependence of MyoD, KMT2D, BAF, and p300 within the context of gene repression.

We thank the reviewer for this insightful comment. The potential role of MyoD in mediating gene repression has recently been shown [4] and was also suggested by our initial GREAT GO analysis of MyoD-bound regions. To directly address this point, we linked MyoD binding to transcriptional outcomes using newly generated, time-course nascent RNA-seq data. Our integrated analysis (revised Fig. 1 and Supplementary Fig. 2) reveals the following:

I. Identification of a transcriptional repression program: Nascent RNA-seq analysis identified distinct clusters of genes (G4-G5) that are repressed following MyoD induction (Supplementary Fig. 2a). GO analysis of this cluster shows enrichment for genes associated with alternative lineage and non-myogenic developmental programs including angiogenesis, ureteric bud development, axon guidance, osteoblast differentiation, and immune responses (Supplementary Fig. 2b).

II. Association of MyoD binding with gene repression: We mapped the repressed gene clusters to MyoD-bound regulatory regions. This analysis revealed that enhancers exhibiting early and transient MyoD binding (clusters A-C) are associated with these repressed genes (Fig. 1g and Supplementary Fig. 2c).

Together, these results demonstrate that MyoD binding is associated not only with transcriptional activation but also with repression, consistent with recent findings [4]. While a detailed mechanistic dissection of how MyoD, KMT2D, cBAF, and p300 coordinate this gene repression is beyond the scope of the present study, we agree with the reviewer's underlying rationale. To address this, we have added a new section to the Discussion titled “MyoD as a dual regulator of gene expression.” This section emphasizes MyoD's dual capacity for gene activation and repression during myogenic reprogramming and highlights the functional interdependence of chromatin modifiers in mediating gene repression as an important direction for future investigation.

4) Fig 1-S1 G shows an intersection of MyoD bound enhancers and histone modifications - H3K4me1 and H3K27ac. However, it is unclear what data the refer to. Cut&Run datasets of H3K4me1 and H3K27ac are not shown. For proper evaluation of the relationship between MyoD and dynamic changes of H3K4me1 and H3K27ac at these enhancers, it is necessary that the authors perform H3K4me1 and H3K27ac Cut&Run analysis before and after MyoD induction. Again, this should be associated with RNAseq.

We thank the reviewer for raising this important point and apologize for the lack of clarity in the original presentation. The H3K4me1 and H3K27ac datasets shown in the original Figure 1-S1E (now revised Figure 1d) were generated by CUT&RUN experiments performed in parallel with MyoD under the same experimental conditions.

To address the reviewer’s request to evaluate dynamic changes in H3K4me1 and H3K27ac at MyoD-bound enhancers and their relationship to transcription, we have performed an integrated temporal analysis, now included in Supplementary Fig. 3. We tracked CUT&RUN signals for

H3K4me1 and H3K27ac at MyoD binding sites associated with differentially regulated genes from 0 h to 24 h. Using unsupervised soft clustering, we classified these temporal patterns into distinct epigenomic trajectories (Supplementary Fig. 3a-b). We then linked these enhancer chromatin states to transcriptional dynamics by mapping the histone mark clusters to gene groups (G1-G5) defined by nascent RNA-seq. This analysis reveals a statistically significant association between enhancer histone marks and transcriptional output. For example, enhancers showing late priming (M3) and/or activation (A3) are enriched near genes with delayed upregulation (G3), whereas repressed gene groups (G4) are associated with decommissioned (M4) and/or deactivated enhancers (A4).

In addition, to provide a representative example, we have included genome browser views of MyoD, H3K4me1, H3K27ac, and nascent RNA signals at the *Myog* locus (Figure 1h). The figure legends and main text have been updated accordingly.

5) It is unclear why the authors decided to perform their experiments in pre-adipocytes. No explaination is provided in the text. Moreover, how was immortalization of pre-adipocytes obtained? This needs to be described, as it could be an operation that might alter MyoD function and chromatin binding.

We have included the rationale for using immortalized preadipocytes in the revised manuscript. We selected preadipocytes as a non-myogenic mesenchymal progenitor model lacking endogenous MyoD and other myogenic factors, thereby providing a system to examine MyoD-dependent chromatin events during trans-differentiation. We have previously demonstrated that ectopic MyoD expression efficiently induces myogenesis of immortalized preadipocytes and associated expression of myogenesis markers such as Myog, Myh and Mck, providing suitability of this model [5].

Immortalization was achieved using SV40 large T antigen, as described in the Methods. All comparisons were done within the same uniformly immortalized population. Moreover, our key findings were reproduced in C2C12 myoblasts as well as in a ligand-induced GR nuclear translocation system.

6) The author’s conclusions are based on the assumption that ARID1A and BRG1 are representative of muscle SWI/SNF complexes, which is instead highly heterogeneous. Different BAF complexes are distributed genome-wide, with some of them being devoid of Brg1 (replaced by the alternative catalytic sub-unit Brm) and ARID1A, as also previously reported in Albini et al (2015) and Sharma et al. (2021). It is recommended that the authors monitor the chromatin binding of additional SWI/SNF components (e.g. BAF47, Brm and BAF60c).

SWI/SNF chromatin remodelers exist in three main subcomplexes with distinct subunit compositions and genomic distributions: canonical BAF (cBAF), PBAF, and ncBAF/GBAF [6]. In

this study, we specifically focused on cBAF, given its well-established role in enhancer regulation and TF-dependent chromatin remodeling [7,8]. Our aim was not to comprehensively characterize various SWI/SNF complexes in muscle, but rather to examine TF-chromatin remodeler interactions within the context of cBAF, using MyoD (and GR) as model systems. We used ARID1A and BRG1 as representatives of cBAF, as ARID1A is the unique/defining subunit of the cBAF, and BRG1 is its primary catalytic ATPase. Thus, our conclusions are intended to reflect ARID1A-associated cBAF recruitment and function. We acknowledge that SWI/SNF complexes in muscle are heterogeneous, including ARID1A-independent and BRM-containing assemblies [9,10]. To avoid overgeneralization, we have revised the manuscript throughout to refer specifically to "cBAF" rather than "BAF". While profiling additional subunits (e.g., BAF47, BRM, BAF60c) would provide further insight, such analyses are beyond the scope of the current study.

References

1 Polesskaya, A. *et al.* CREB-binding protein/p300 activates MyoD by acetylation. *J Biol Chem* **275**, 34359-34364, doi:10.1074/jbc.M003815200 (2000).

2 Sartorelli, V. *et al.* Acetylation of MyoD directed by PCAF is necessary for the execution of the muscle program. *Mol Cell* **4**, 725-734, doi:10.1016/s1097-2765(00)80383-4 (1999).

3 McLean, C. Y. *et al.* GREAT improves functional interpretation of cis-regulatory regions. *Nat Biotechnol* **28**, 495-501, doi:10.1038/nbt.1630 (2010).

4 Nicoletti, C. *et al.* MYOD represses gene expression from non-E-box motifs. *Genes Dev* **39**, 1299-1317, doi:10.1101/gad.352708.125 (2025).

5 Lee, J. E. *et al.* H3K4 mono- and di-methyltransferase MLL4 is required for enhancer activation during cell differentiation. *Elife* **2**, e01503, doi:10.7554/eLife.01503 (2013).

6 Mashtalir, N. *et al.* Modular Organization and Assembly of SWI/SNF Family Chromatin Remodeling Complexes. *Cell* **175**, 1272-1288 e1220, doi:10.1016/j.cell.2018.09.032 (2018).

7 Mathur, R. *et al.* ARID1A loss impairs enhancer-mediated gene regulation and drives colon cancer in mice. *Nat Genet* **49**, 296-302, doi:10.1038/ng.3744 (2017).

8 Park, Y. K. *et al.* Interplay of BAF and MLL4 promotes cell type-specific enhancer activation. *Nat Commun* **12**, 1630, doi:10.1038/s41467-021-21893-y (2021).

9 Albini, S. *et al.* Brahma is required for cell cycle arrest and late muscle gene expression during skeletal myogenesis. *EMBO Rep* **16**, 1037-1050, doi:10.15252/embr.201540159 (2015).

10 Sharma, T., Robinson, D. C. L., Witwicka, H., Dilworth, F. J. & Imbalzano, A. N. The Bromodomains of the mammalian SWI/SNF (mSWI/SNF) ATPases Brahma (BRM) and Brahma Related Gene 1 (BRG1) promote chromatin interaction and are critical for skeletal muscle differentiation. *Nucleic Acids Res* **49**, 8060-8077, doi:10.1093/nar/gkab617 (2021).

REVIEWERS' COMMENTS

We are grateful to the reviewers for their highly supportive evaluation of our revised manuscript and their recommendation for publication in *Nature Communications*.

Reviewer #1 (Remarks to the Author):

The authors have thoughtfully and fully addressed each of my critiques.

We sincerely thank the reviewer for their time, valuable feedback, and supportive endorsement of our manuscript.

Reviewer #2 (Remarks to the Author):

I co-reviewed this manuscript with one of the reviewers who provided the listed reports. This is part of the Nature Communications initiative to facilitate training in peer review and to provide appropriate recognition for Early Career Researchers who co-review manuscripts.

Reviewer #3 (Remarks to the Author):

This revised manuscript now includes new data and a markedly expanded discussion; that have significantly improved this work. I appreciate that the authors made the effort to add nascent RNA transcriptome analysis to this work and also evaluated the ability of a MyoD mutant that lacks acetylation sites; with consequent interesting new insights! In addition, the more encyclopedic nature of the discussion is a significant improvement; as this work can now be better appreciated in the context of a large body of literature on TF-chromatin modifier interactions/synergy. In summary, this revised manuscript merits publication in Nature Communications in its present form. I congratulate the authors on a nice piece of very rigorous work.

We are deeply grateful for the enthusiastic feedback and are pleased that the new experimental data and expanded discussion successfully strengthened the manuscript.

Reviewer #4 (Remarks to the Author):

In the revised version of the manuscript the authors have addressed the most important

issues raised by the reviewers and the manuscript is definitely improved and suitable for publication, in the opinion of this reviewer.

We sincerely thank the reviewer for their careful evaluation of our revisions and for recommending our manuscript for publication.
